# Eliciting risk preferences that predict risky health behavior: A comparison of two approaches

**DOI:** 10.1002/hec.4486

**Published:** 2022-02-22

**Authors:** Murong Yang, Laurence S. J. Roope, James Buchanan, Arthur E. Attema, Philip M. Clarke, A. Sarah Walker, Sarah Wordsworth

**Affiliations:** ^1^ Nuffield Department of Population Health Health Economics Research Centre University of Oxford Oxford UK; ^2^ NIHR Oxford Biomedical Research Centre John Radcliffe Hospital University of Oxford Oxford UK; ^3^ NIHR Health Protection Research Unit (HPRU) in Healthcare Associated Infections and Antimicrobial Resistance University of Oxford Public Health England (PHE) Oxford UK; ^4^ Erasmus School of Health Policy & Management Rotterdam The Netherlands; ^5^ Nuffield Department of Medicine John Radcliffe Hospital University of Oxford Oxford UK

**Keywords:** framing effects, lottery elicitation approach, risk preference, risky health behavior

## Abstract

Information on attitudes to risk could increase understanding of and explain risky health behaviors. We investigate two approaches to eliciting risk preferences in the health domain, a novel “indirect” lottery elicitation approach with health states as outcomes and a “direct” approach where respondents are asked directly about their willingness to take risks. We compare the ability of the two approaches to predict health‐related risky behaviors in a general adult population. We also investigate a potential framing effect in the indirect lottery elicitation approach. We find that risk preferences elicited using the direct approach can better predict health‐related risky behavior than those elicited using the indirect approach. Moreover, a seemingly innocuous change to the framing of the lottery question results in significantly different risk preference estimates, and conflicting conclusions about the ability of the indicators to predict risky health behaviors.

## 1 INTRODUCTION

An individual's attitude toward risk affects their behavior under conditions of uncertainty. Those inclined to be risk seeking are more likely to engage in risky behaviors, such as purchasing portfolios with higher rates of return and greater volatility (Guiso & Paiella, 2004; Pålsson, 1996) or choosing jobs with less certain financial rewards (Diaz‐Serrano & O'Neill, 2004). Risk preferences are also associated with health‐related behavior such as smoking, heavy consumption of alcohol (Beauchamp et al., 2017), demand for healthcare utilization (Lutter et al., 2019), and compliance with social distancing during the COVID‐19 pandemic (Müller & Rau, 2020). Having better information on risk preferences is not only useful in understanding individual behavior, but also in designing interventions to prevent risk‐taking behaviors that have negative effects on individuals or society (Mata et al., 2018).

To date, various approaches have been taken to elicit individuals' attitudes toward risks, such as assessing observed behaviors, lottery tasks, and self‐reported scales (Lutter et al., 2019). Among these approaches, self‐reported scale measures have gained attention. Self‐reported measures of risk preference can be embedded into questionnaires asking respondents risk‐related questions on a scale indicating the extent to which the respondent is willing to take risks. A simple self‐reported approach, which originates from the German Socio‐Economic Panel (SOEP) survey, directly asks a single question about people's attitudes toward risk; we refer to this hereafter as the “direct approach” to estimating attitudes toward risks. Dohmen et al. (2011) showed that this direct question provides estimates of risk preference that are useful for making predictions about individuals' actual willingness to take risks. Moreover, they found that answers to a direct question about willingness to take risks in the health domain were particularly useful for predicting risky health behaviors.^1^ While the direct approach is non‐incentivized, it is simple to implement (Charness et al., 2013) and has been shown to be an effective instrument in eliciting attitudes toward risks, in both a general and a health domain, that are predictive of actual risky behavior (Daly et al., 2010; Lutter et al., 2019; Nebout et al., 2018).

An alternative approach, traditionally used in economics, including health economics, is to elicit attitudes toward risks indirectly via lottery questions with financial outcomes. Lottery questions can take different forms, such as a single lottery question (Gneezy & Potters, 1997), a single choice among multiple gambles (Eckel & Grossman, 2002, 2008), and a price list approach with a series of gambles (Holt & Laury, 2002). This approach elicits information on risk aversion when respondents transfer from one option to another. Risk preference estimates elicited from lottery approaches with monetary outcomes have been used to explain various phenomena including violence (Callen et al., 2014), market behavior (Fellner & Maciejovsky, 2007) and farmers' production decisions (Ward & Singh, 2015). A number of subsequent health economic studies have also used income lottery questions to elicit risk preferences (Bessey, 2018; Dave & Saffer, 2007; Szrek et al., 2012).

However, there are some concerns with eliciting risk preferences using lottery approaches. One concern is whether risk preferences elicited from lottery approaches with financial outcomes have strong predictive validity in non‐economic domains. Anderson and Mellor (2008) conducted an experiment using the lottery approach designed by Holt and Laury (2002) and found only weak evidence that people's attitudes toward risk were associated with cigarette smoking, heavy drinking, and not wearing seat belts. Szrek et al. (2012) used the same lottery approach and found that risk preferences elicited from monetary gambles fail to predict risky health behaviors. Moreover, risk preferences elicited across different domains have been found to vary (Dohmen et al., 2011; Weber et al., 2002). Thus, it is conceivable that lottery elicitation methods using health outcomes, rather than monetary outcomes, might deliver reliable risk preferences well suited to predict health‐related behavior. Some studies have used health outcomes in preference elicitation exercises (Arrieta et al., 2017; Attema et al., 2013, 2019; Galizzi et al., 2016; Krieger & Mayrhofer, 2012; Rouyard et al., 2018; van der Pol & Ruggeri, 2008); however, there is little evidence on the validity of risk preferences elicited from lottery questions about health outcomes.

Another concern relates to potential framing effects in gamble questions. A framing effect may arise if people appear to be more or less risk averse simply due to the way lottery questions are presented (Slovic & Lichtenstein, 1983; Wang, 1996). Decision‐making processes may be affected by visual factors such as the position and emotional states of items, leading to biased estimates of real preference (Orquin et al., 2018). There is a substantial body of evidence that framing effects in lottery questions can have a major impact on choices and risk preference estimates (Gonzalez et al., 2005; Kahneman & Tversky, 1986; Kwak & Huettel, 2018; Lévy‐Garboua et al., 2012). Framing effects in the elicitation of risk preferences could also potentially affect their predictive power in regression models and bias study results. Kjellsson et al. (2014) found that using different length of recall periods in health survey questions affects the size and significance of estimates of the association between education and hospitalization. However, there is little evidence on whether framing risk elicitation lottery questions differently would affect their ability to predict risky behaviors.

Previous studies have compared risk preference estimates elicited from direct and lottery approaches in other (non‐health) domains, and the estimates have been found to differ across the two approaches (Crosetto & Filippin, 2013). Risk preferences elicited from the direct method had high stability and strong predictive power in predicting risky behaviors such as choice of portfolio and the amount of money that respondents would like to transfer in a trust game, while the risk preferences elicited from monetary lottery approaches had little or no correlation with risky behaviors (Coppola, 2014; Kapteyn & Teppa, 2011; Lönnqvist et al., 2015). Similar results were also found when eliciting risk attitudes to predict health‐related behaviors, using the direct approach and the lottery approach with monetary outcomes (Beauchamp et al., 2017; Szrek et al., 2012). However, it remains unclear whether risk preferences elicited using lottery approaches with health outcomes can better predict broad health‐related behaviors than the direct scale approach. In a study of General Practitioners (GPs) in France, Massin et al. (2018) compared the predictive power of a health lottery elicitation method with that of a direct health scale approach. The health lottery approach used involved a series of gambles where GPs were asked to choose between two therapies with different health outcomes, assuming respondents aged 70 years; the direct scale approach asked GPs their risk attitudes toward their own health and patients' health, using the same format as the approach used in Dohmen et al. (2011). The two approaches were found to perform similarly well. However, to the best of our knowledge, this has not yet been confirmed in a general population sample.

The evidence on the direct scale and lottery approaches motivates a question about the validity of lottery approaches in the health domain, compared with the direct scale approach, for eliciting risk preference estimates in a general population. In this paper, we contribute to this line of inquiry in several respects. First, we used a novel indirect lottery elicitation method with health states as outcomes to elicit attitudes toward risks (we refer to this hereafter as the “indirect approach”),^2^ and aimed to test the predictive ability of the derived risk preferences on risky health behaviors.^3^ Second, instead of using a sub‐population (e.g., as in Massin et al., 2018), we used a general adult population to examine whether this lottery approach can predict risky health behaviors more or less well than a direct scale approach. To achieve this, we conducted a survey of a representative sample of the adult general population in the United Kingdom, asking respondents about a variety of risky health behaviors and eliciting their attitudes toward risks using these two approaches. Third, we tested for a potential framing effect in the indirect lottery approach by presenting two versions of the lottery questions. Respondents were allocated randomly to receive the two versions, which differed only in the framing of questions. As well as testing whether the different framing led to different risk preference estimates, we investigated whether the resulting estimates from the two versions differed to predict risky health behaviors.

We have three main results. First, risk preferences elicited using the direct scale approach are stronger predictors of health‐related risky behavior than those elicited using the indirect approach. Second, consistent with previous studies, the risk indicators specific to the health domain are better than the more general risk indicators for predicting risky health behaviors (Dohmen et al., 2011; Massin et al., 2018). Third, there is a significant framing effect in the indirect lottery approach, which results not only in significantly different risk preference estimates, but also in conflicting conclusions about the ability of the indicators to predict risky health behaviors. This framing effect relates to the apparent unavoidability in lottery questions of presenting one option before the other in some way, for example, by labeling it as “Option 1”, or by placing it above or to the left of the second option. The effect demonstrated could therefore have broader significance for other lottery‐based methods.

This article is organized as follows. Section 2 outlines methods and Section 3 provides the main results. Section 4 presents a sensitivity analysis to assess the generalizability of the results. Section 5 discusses the results and provides a conclusion.

## 2 DATA AND METHODS

### 2.1 Data collection

We conducted a web‐based survey, which asked people about their attitudes toward risk using the indirect and the direct approach.^4^ The survey was conducted with a panel of respondents provided by Survey Sampling International (SSI, now called Dynata), a data collection and market research company. SSI was commissioned to collect a sample that was representative of the UK adult population in terms of sex, age, ethnicity, and geographic region. Survey invitations were sent to 8317 UK residents who were SSI panel members and data collection was completed over 22 days in March 2017. It was also possible for SSI panel members to access the survey via SSI's website, without receiving an invitation by email.

In the survey, respondents were asked to provide a range of personal information that might be associated with risk preferences, including socio‐demographic characteristics such as age, gender, ethnicity, own self‐rated health score, and geographic region, and the so‐called “Big Five” personality traits—“Extraversion,” “Agreeableness,” “Conscientiousness,” “Neuroticism,” and “Openness.”^5^ Respondents also answered questions regarding several health‐related behaviors. These related to smoking status, alcohol consumption, and adherence to completing a course of antibiotics when ill.

### 2.2 Approaches to eliciting risk preferences in the study

#### 2.2.1 The direct approach

The direct approach questions were adapted from those in the German SOEP survey, where respondents were asked questions about their willingness to take risks in both the health domain and in a more general domain. In the health domain, the question was: “*How would you rate your willingness to take risks with your health? Please write a number between 0 and 10 in the box below, where 0 means ‘Not at all prepared to take risks’ and 10 means ‘Fully prepared to take risks’*.” In the more general domain, the question was: “*How do you see yourself? Are you generally a person who is fully willing to take risks or do you try to avoid taking risks? Please write a number between 0 and 10 in the box below, where 0 means ‘Not at all prepared to take risks’ and 10 means ‘Fully prepared to take risks’*.” If a respondent provided a higher score in this question, this indicates that the respondent was more risk seeking.

#### 2.2.2 The indirect approach with health states

The indirect lottery approach developed by Roope et al. (2018) and applied in our survey is based on the price list approach using standard gamble. The price list approach is a commonly applied risk measure and has several derivations (Csermely & Rabas, 2016). Popularized by Holt and Laury (2002), it is favored among lottery approaches because of its transparency to subjects (Andersen et al., 2006) and its finer categorization of individuals’ risk preferences (Dave et al., 2010). The price list format is a series of gambles shown in multiple rows in a table, with each row including a gamble with two options. Holt and Laury (2002) used paired gambles where the two options were both lotteries and the probabilities in these lotteries changed across rows. However, this approach generates noisy estimates of subjects' risk preferences because it involves probabilities and monetary payoffs that are harder for subjects with lower numerical skills to understand (Dave et al., 2010). Thus, in the indirect approach, rather than using paired lotteries, we simplified the approach by using a standard gamble in each row, in which one option is a lottery and another option is a certain outcome. A similar approach was implemented by Bruner (2009), and was found to have high predictive power in investment settings among nine monetary lottery tasks based on the price list format (Csermely & Rabas, 2016). We also used health outcomes instead of financial outcomes in the indirect approach to examine the predictive power of a lottery approach grounded in the health domain. In the indirect approach, respondents answered a series of hypothetical questions about their risk preferences, in which they were asked to choose between an option with a certain outcome and an option with an uncertain outcome. Outcomes in the novel indirect approach were three health states: Full health, Health State A and Health State B. The health states were designed in consultation with clinical experts (*n* = 3; primary care physician and two junior doctors) and a public and patient involvement panel (*n* = 7).^6^

In the survey, Health State A was described as having a temperature, aching muscles, a headache, a dry chesty cough, a sore throat, and feeling weak; Health State B was described as having a temperature, chest pain, night sweats, a cough that brings up phlegm, loss of appetite (not wanting to eat), feeling drained, and having lost some weight. Health State A signifies better health than Health State B, but was intended to be worse than Full Health. Respondents were asked to rate these health states on a scale from 0 (the worst possible health state) to 10 (the best possible health state, also described as “Full Health”).

After rating the health states, respondents answered questions about attitudes toward risks, framed as “standard gambles” with two options. In one option, there was a chance of Full Health but also a chance of Health State B, while the other option had the certain outcome of Health State A. The indirect approach involved asking a series of questions where the probability of getting Full Health in the lottery option varied in each question.

For example, respondents made a choice between either Option 1, with a 50% chance of Full Health and a 50% chance of Health State B, or Option 2 with a 100% chance of Health State A. If respondents chose Option 2, they were given another question which offered 90% chance of Full Health in Option 1. Respondents who still chose the alternative with a certain outcome in this scenario were asked to write a number in a box indicating how low the probability of Health State B would need to be in order for them to choose the gamble. The question was as follows: “*I would prefer Option 1 to Option 2, if the chance of Health State B in Option 1 was less than 1 out of [].”* Respondents who chose Option 1 in the first question, with a 50% chance of Full Health, were then asked to choose between either a gamble with a 10% chance of Full Health and a 90% chance of Health State B, or a 100% chance of Health State A. After this, respondents continued to answer a series of similar questions, where the probability of getting Full Health varied in each question. See survey question number 6 in Appendix A for full details of the series of questions.

**FIGURE 1:**
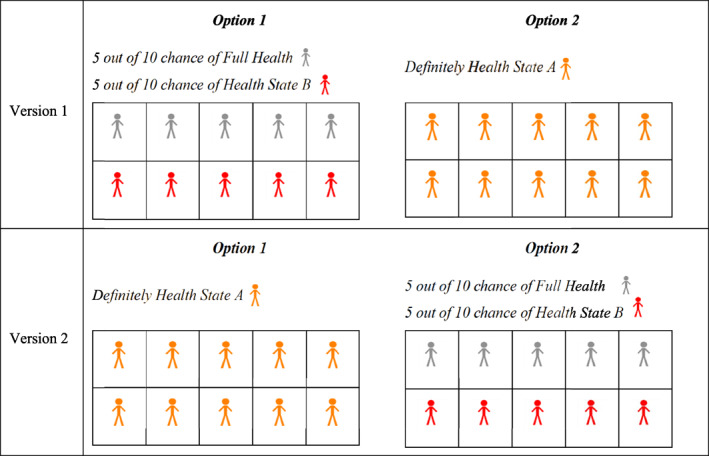
The two versions of the first of the series of lottery elicitation questions

Additionally, to test for a potential framing effect in our indirect approach, respondents were randomized to receive two versions of these lottery questions. As illustrated in Figure 1, in Version 1, the uncertain alternative with two possible outcomes was placed on the left‐hand side of the screen and labeled “Option 1”; the other alternative, with a certain outcome, was placed on the right‐hand side and labeled “Option 2”. In Version 2, the labeling and placement on screen of the options were reversed. That is, the option with a certain outcome was placed on the left‐hand side and labeled “Option 1”; the other option, with two possible outcomes, was placed on the right‐hand side and labeled “Option 2”.

### 2.3 Constructing risk indicators from survey responses

Risk indicators were constructed from the answers to the survey, with the aim of comparing the predictive power of risk indicators elicited via the direct and indirect approaches, and testing whether framing the indirect lottery questions differently—as Version 1 versus Version 2—affected the risk preference estimates and/or their ability to predict risky health behaviors.

In the direct approach, attitudes toward risks were measured on a scale from 0 to 10, with higher scores indicating greater risk seeking. Two scores for each respondent were obtained using direct questions about willingness to take risks—with health and in general.

To construct risk indicators using the indirect approach, any respondents who evaluated Health State A equal to or worse than B, or Health State A equal to Full Health, were dropped, restricting the available sample. This was because the lottery questions were designed in a way that required respondents to recognize that Health State A is better than Health State B but worse than Full Health. In other words, risk indicators from the indirect approach could only be estimated among the subsample of respondents who recognized that Health State A was better than Health State B and worse than Full Health (see Figure A1 for more details). Thus, the comparison between the direct and indirect approaches was conducted using this subsample, which is referred to hereafter as our “restricted” sample, rather than the full sample.^7^

Risk indicators from the indirect approach were constructed based on the concept of a “risk premium.” The “risk premium” variable used is analogous to the conventional risk premium in economics, which signifies the additional amount of money an individual requires from a risky asset in order to choose this rather than a riskless asset with the same expected value. People who are more risk averse would pay more to avoid choosing an option with uncertainty. That is, the more risk averse, the higher the risk premium. Thus, the risk premium may be used as an indicator of attitudes toward risks. Risk premiums are usually measured with regard to utility from money, but in our study we measured the risk premium in terms of health states. The risk premium in our study can be defined as the difference between the expected utility from a gamble and the utility from the certain option. Formally,

Riskpremium=E[U(thegambleoption)]−U(thecertainoption)

(1) $$=\left[{p_{B}}^{*}\times U_{B}+\left(1-{p_{B}}^{*}\right)\times U_{F}\right]-U_{A}$$

where $\;{p_{B}}^{*}$ is the probability of getting Health State B for which the respondent is indifferent between taking the gamble and choosing the certain option, and $U_{A}$, $U_{B}$, and $U_{F}$ are the utilities^8^ of Health State A, Health State B, and Full Health (i.e., $U_{F}=10$), respectively. See Figure A2 for an example of the calculation process.

For respondents who were extremely risk averse, ${p_{B}}^{*}$ was obtained from a number that they provided indicating how low the probability of Health State B would need to be for them to be indifferent between the gamble and the certain option. For respondents who completed the series of lottery questions, ${p_{B}}^{*}$ can be estimated as the midpoint of the probabilities of the questions where the respondent switched between choosing Option 1 and Option 2. For example, consider a respondent who chose the certain option under a 40% chance of Health State B (i.e., 60% of Full Health) in the gamble option, but chose the gamble option under a 30% chance of Health State B. This indicates that ${p_{B}}^{*}$ is between 0.3 and 0.4. The survey responses only enable us to elicit the range that ${p_{B}}^{*}$ lies in, rather than an exact number. For the convenience of estimation, ${p_{B}}^{*}$ was estimated as the midpoint of the range, which is 0.35 in the example above.

To compare the predictive power of the indirect approach to that of the direct approach in the restricted sample, we created a consistent overall indicator from the indirect approach across the two versions of the restricted sample, allowing for the possibility that, due to a framing effect, the mean risk premium could be different in the two versions. First, we constructed the risk premiums for each of Versions 1 and 2. We then created an overall risk premium variable across the two versions as follows:

(2) $$\;R_{1}=r_{1}+0.5\times d$$

(3) $$R_{2}=r_{2}-0.5\times d$$

where $R_{i}$ and $r_{i}$ are adjusted and unadjusted risk premiums in version *i*, respectively; and *d* is the difference between means of the two risk premiums. The unadjusted risk indicator r and adjusted risk indicator R can be defined as

(4) $$r=\left\{r_{1},r_{2}\right\}$$

(5) $$R=\left\{R_{1},R_{2}\right\}$$

### 2.4 Analysis of risk preferences and risky health behavior

A framing effect may arise in the elicitation of risk preferences using the indirect approach. To test for this, we first compared the means of the risk indicators elicited from Version 1 (gamble on the left‐hand side) with those elicited from Version 2 (gamble on the right‐hand side) using a *t*‐test. Differences between the two versions in terms of other respondent characteristics were compared with *t*‐tests and chi‐squared tests. We further tested for framing effects in the regression framework. To achieve this, a binary variable, “Version 1,” was constructed as the independent variable, where 1 indicates respondents were randomized to answer lottery questions in Version 1 and 0 indicates Version 2. The dependent variable is the unadjusted risk indicator r in Equation (4). We also controlled for a set of variables to assess whether any difference in risk indicator estimates between Versions 1 and 2 is caused by the framing of questions per se rather than any imbalances of other factors between the two versions. The regression model can be written as follows:

(6) $$r=\alpha_{0}+\alpha_{1}Respondent\;in\;Version\;1+\delta X+\varepsilon$$

where X is a vector of covariates, including socio‐demographic indicators and personality traits; *δ* is a vector of coefficients of covariates; and *ε* is the disturbance term. The empirical analysis started with the basic regression Model 1 where age, age squared/100, gender, ethnicity (white/black and ethnic minority), and whether respondents were born in the United Kingdom were included as covariates. Model 2 repeated the same estimation adding socio‐economic variables as controls with the “Big Five” personality traits added in Model 3. The regression models used Ordinary Least Squares (OLS) estimations. The relative quality of Models 1–3 was examined using the Akaike information criterion (AIC) and Bayesian information criterion (BIC) as well as adjusted *R* ^2^.^9^ All estimation regressions report robust standard errors. A type of RESET test was performed as a test of model specification.^10^ As respondents in Versions 1 and 2 were allocated randomly, if the risk indicators of respondents in Version 1 are significantly different from those in Version 2, even after controlling for covariates, this indicates a potential framing effect in the indirect approach.

**FIGURE 2:**
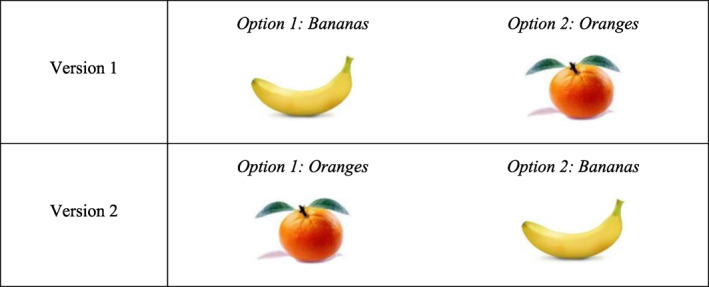
Two versions of the preferred fruit question

If any framing effects were to be detected, it would be valuable to investigate whether this is likely to be a feature of our relatively complex risk questions, or whether an analogous framing effect would arise in a less cognitively demanding question. In the survey, respondents were asked whether they preferred bananas or oranges. Respondents were randomly assigned one of two versions of this question, where the framing of the question differed in a very similar way as in the indirect lottery questions (shown in Figure 2).

We then implemented regression models to estimate the ability of the risk indicators elicited from the direct and indirect approaches to predict risky health behaviors, where the dependent variables are risky health behaviors and the key independent variables are risk indicators from the direct and indirect approach, respectively. The dependent variables are risky health behaviors, which include “smoking status,” being a “regular smoker,” “frequency of drinking five or more alcoholic drinks,” and “non‐adherence to taking a full course of antibiotics” prescribed by a doctor, where being a “regular smoker” is a binary variable and the others are ordinal variables.^11^ We also constructed two summary measures of risky health behaviors. The first was a binary “any risky behavior” variable equal to 1 if respondents reported any of the three risky behaviors, and equal to 0 otherwise. The second was the number (out of three) of the risky health behaviors reported. Respondents were treated as having at least one risky health behavior if they were a regular smoker, regular drinker or probably not/definitely not adherent to taking a full course of antibiotics. In the indirect approach, the risk indicator is represented by the risk premium: the higher the risk premium, the more risk averse people are. However, in the direct approach, the higher the score on the scale from 0 to 10, the more people prefer taking risks. To make the signs of the risk indicators comparable in the two approaches, signs of risk indicators in the indirect approach were reversed in the regressions so a higher score indicates that people are more risk seeking. All risk indicators were standardized (setting mean equal to zero and standard deviation equal to (1) in the empirical models in order to make the association between the measures and risky health behavior comparable.

The regression model can be specified as

(7) $$Risky\;health\;behaviour=\beta_{0}+\beta_{1}risk\;indicator+\theta X+u$$

where **X** is a vector of covariates which are the same as those in Equation (6); θ is a vector of coefficients of covariates; and u is the disturbance term. The regressions used probit and ordered probit models. Model specifications were assessed as described above for the regression models in Equation (6). Pseudo *R* ^2^ rather than adjusted *R* ^2^ was reported in Equation (7).

There is a potential source of unobserved heterogeneity with respect to subjective elements, such as respondents' understanding of health and their health histories, which might be correlated with both the risk indicators (the independent variables) and the risky health behaviors (the dependent variables). This could bias estimates of the association between risk indicators and risky health behaviors. In addition to controlling for respondents' own self‐rated health and health status (i.e., whether sick or disabled) in the regression models above, as a robustness check, we also controlled for respondents' subjective view of Health State A and Health State B.

## 3 RESULTS

### 3.1 Summary statistics

Table 1 describes the characteristics of respondents in the full and restricted (with risk preferences) samples. Comparing the full and restricted sample, variables were similar in terms of age, income, employment, sick/disabled, married/partner, and geographic region, except for London (*p* < 0.0001) and the South west (*p* = 0.07). Risky health behaviors, such as smoking and non‐adherence to taking a full course of antibiotics when prescribed, differed by modest degrees, while alcohol consumption was more similar across the full and restricted sample. Respondents in the restricted sample had slightly lower “Extraversion” (*p* = 0.0001) and more “Neuroticism” (*p* = 0.01) and “Openness” (*p* = 0.02). As any imbalances between the two samples could potentially affect the validity of the results, this is explored in a sensitivity analysis in Section 4.

**TABLE 1:** Respondent characteristics in the full and restricted sample^a^

Variable	Full sample			Restricted sample with risk preference			*p* Value^b^
	*N* with data	*n*	%	*N* with data	*n*	%	
Smoking status							
Non‐smoker	4000	2839	70.9	2612	1975	75.6	<0.0001
Occasional smoker	4000	282	7.1	2612	167	6.4	0.03
Regular smoker	4000	879	22.0	2612	470	18.0	<0.0001
Adherence to taking the full course of antibiotics							
Definitely	4000	2913	72.8	2612	2003	76.7	<0.0001
Probably	4000	749	18.7	2612	436	16.7	<0.0001
Probably not	4000	205	5.1	2612	108	4.1	<0.0001
Definitely not	4000	60	1.5	2612	28	1.1	0.002
Drinking status							
Non‐drinker	4000	589	14.7	2612	354	13.6	0.004
Occasional drinker	4000	1557	38.9	2612	1048	40.1	0.03
Regular drinker	4000	1854	46.4	2612	1210	46.3	0.97

	*N* with data	Mean	SD	*N* with data	Mean	SD	
Frequent drinker^c^	3411	2.2	1.2	2258	2.1	1.1	<0.0001
Risk indicators in the direct approach							
Indicators in general domain	4000	5.2	2.4	2612	5.0	2.4	<0.0001
Indicators in health domain	4000	3.8	2.6	2612	3.6	2.5	<0.0001
Socio‐demographics							
Age	4000	46.5	16.8	2612	46.5	16.6	0.80
Household equivalent income	3689	20,269	15,039	2415	20,473	14,770	0.26
Own self‐rated health (0–10)	4000	7.3	2.0	2612	7.5	1.8	<0.0001

	*N* with data	*n*	%	*N* with data	*n*	%	
Male	3996	1941	48.6	2611	1192	45.7	<0.0001
White	3978	3606	90.6	2598	2382	91.7	0.002
Christian	3907	1858	47.6	2571	1192	46.4	0.04
Higher education	4000	1756	43.9	2612	1213	46.4	<0.0001
Unemployed	4000	184	4.6	2612	113	4.3	0.26
Sick or disabled	4000	162	4.1	2612	108	4.1	0.71
Married/civil partnership/live with a partner	4000	2668	66.7	2612	1720	65.8	0.12
Born in UK	4000	3604	90.1	2612	2381	91.2	0.002
Geographic region							
East Anglia	4000	372	9.3	2612	263	10.1	0.02
East Midlands	4000	290	7.3	2612	197	7.5	0.33
West Midlands	4000	354	8.9	2612	238	9.1	0.42
London	4000	516	12.9	2612	286	10.9	<0.0001
North East	4000	168	4.2	2612	119	4.6	0.12
North West	4000	450	11.3	2612	282	10.8	0.21
South East	4000	551	13.8	2612	353	13.5	0.51
South West	4000	343	8.6	2612	234	9.0	0.24
Yorkshire & Humberside	4000	337	8.4	2612	235	9.0	0.07
Wales	4000	197	4.9	2612	129	4.9	0.96
Scotland	4000	318	8.0	2612	207	7.9	0.94
Northern Ireland	4000	104	2.6	2612	69	2.6	0.82
Personality traits^d^							

	*N* with data	Mean	SD	*N* with data	Mean	SD	
Extraversion	4000	5.8	2.0	2612	5.7	2.0	0.0001
Agreeableness	4000	7.0	1.7	2612	7.0	1.7	0.85
Conscientiousness	4000	7.7	1.7	2612	7.7	1.7	0.07
Neuroticism	4000	5.7	2.2	2612	5.8	2.2	0.01
Openness	4000	6.8	1.7	2612	6.9	1.7	0.02

*Note*: SD represents standard deviation.

^a^ Full sample contains the whole *N* = 4000 respondents and the restricted sample contains *N* = 2612 respondents who recognized that Health State A is better than Health State B but worse than Full Health.

^b^ *p* Values from *t*‐tests for continuous and chi‐squared test for categorical factors compare those with and without risk preference indicators.

^c^ “Frequent drinker” is an indicator to represent the frequency of drinking five or more alcoholic drinks on one occasion, which was measured on a scale from 1 to 5, where 5 indicates the highest frequency.

^d^ Extraversion, Agreeableness, Conscientiousness, Neuroticism and Openness were measured on a scale from 2 to 10.

### 3.2 Risk attitudes elicited from the direct and indirect approach

**FIGURE 3:**
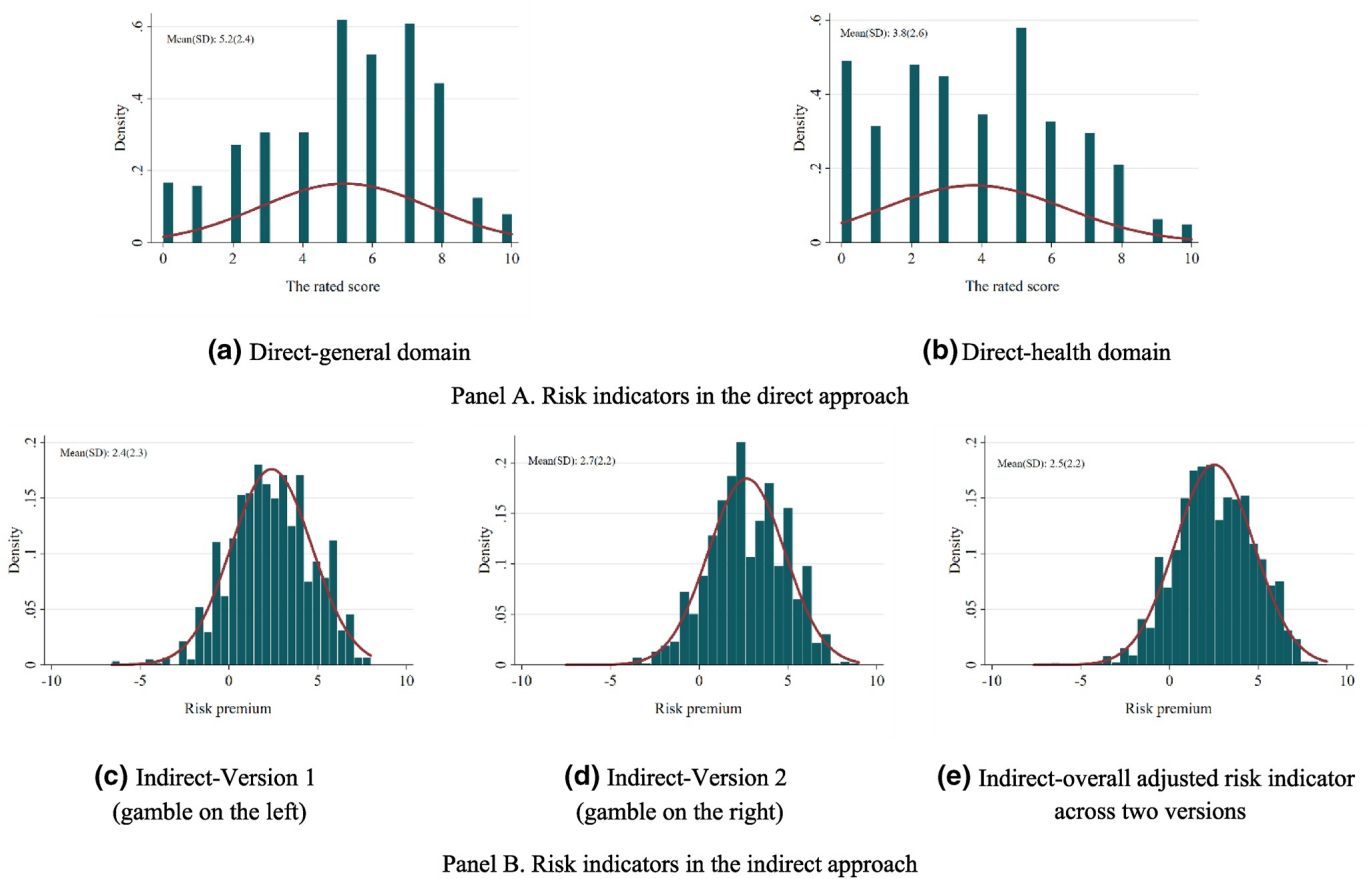
Distributions of risk indicators in the direct and indirect approaches

Figure 3 shows the distributions of risk indicators from the direct and indirect approaches.^12^ By way of comparison, normal distributions with the same mean and standard deviation as the sample data are superimposed. Panel A shows heterogeneity among attitudes toward risks in different domains in the direct approach. As a higher direct risk indicator indicates a higher willingness to take risks, people were less willing to take risks with their health than in general. Panel B indicates approximately normal distributions for the three risk indicators in the indirect approach (see Table A2 for details). In contrast to direct risk indicators, a higher indirect risk indicator indicates a lower willingness to take risks.

Table 2 examines the correlations of different risk measures in the restricted sample. As the signs of direct and indirect risk indicators are reversed, the correlation of risk measures between these two approaches being negative indicated consistency. Comparing the magnitude of coefficients in different risk measures, the direct indicators in the general and health domains are more highly correlated than the direct and indirect indicators. The three indirect indicators are all strongly correlated with the two direct indicators at least at the 5% significance level, while they have a higher correlation with the direct indicator in the health domain than the general domain. Among the three indirect indicators, the indicator in Version 1 has a higher correlation with direct indicators than the other two indirect indicators, indicating differences in risk preference estimates in the two versions.

**TABLE 2:** Correlations between risk attitudes in different elicitation approaches

	Direct‐general	Direct‐health	Indirect‐overall	Indirect‐Version 1	Indirect‐Version 2
Direct‐general	1.000				
	*N* = 2612				
Direct‐health	0.327***	1.000			
	*N* = 2612	*N* = 2612			
Indirect‐overall	−0.075***	−0.093***	1.000		
	*N* = 2612	*N* = 2612	*N* = 2612		
Indirect‐Version 1	−0.104***	−0.108***	1.000***	1.000	‐
	*N* = 1307	*N* = 1307	*N* = 1307	*N* = 1307	‐
Indirect‐Version 2	−0.045**	−0.079***	1.000***	‐	1.000
	*N* = 1305	*N* = 1305	*N* = 1305	‐	*N* = 1305

*Note*: Coefficients of Kendall's tau‐*b* correlation were reported. ***, ** represent significance at the 1% and 5% significance level, respectively.

### 3.3 Tests for framing effects in the indirect approach

Table 3 compares variables in Version 1 (gamble on left‐hand side) and Version 2 (gamble on right‐hand side) using the restricted sample with risk preference information. The distributions of most variables were very similar in both versions; “unemployed,” “married/civil partnership/live with a partner,” and “Openness” had small imbalances (one characteristic with *p* < 0.05 out of 28 tested, two with *p* < 0.10), consistent with the randomization. However, the risk indicator in Version 1 differed significantly from that in Version 2 (*p* = 0.002), with the mean lower in Version 1 (2.4) than in Version 2 (2.7). In the indirect approach, the higher the risk indicator, the more people are deemed risk averse, indicating that respondents in Version 2 (gamble on right‐hand side) were judged more risk averse than those in Version 1 (gamble on left‐hand side).

**TABLE 3:** Respondents' characteristics in two versions of survey questions

Variable	Version 1 (gamble on left)			Version 2 (gamble on right)			*p* Value^a^
	*N* with data	Mean	SD	*N* with data	Mean	SD	
The indirect risk indicator	1307	2.4	2.3	1305	2.7	2.2	0.002
Socio‐demographics							
Age	1307	46.5	16.6	1305	46.6	17	0.86
Household equivalent income	1195	20,189	13,634	1220	20,750	15,804	0.35
Own self‐rated health (0–10)	1307	7.5	1.7	1305	7.5	2	0.52

	*N* with data	*N*	%	*N* with data	*n*	%	
Male	1307	615	47.1	1304	577	44.1	0.15
White	1302	1195	91.8	1296	1187	91.6	0.86
Christian	1284	602	46.9	1287	590	46.0	0.60
Higher education	1307	613	46.9	1305	600	45.8	0.64
Unemployed	1307	69	5.3	1305	44	3.3	0.017
Sick or disabled	1307	60	4.6	1305	48	3.7	0.24
Married/civil partnership/live with a partner	1307	881	67.4	1305	839	64.6	0.09
Born in UK	1307	1194	91.4	1305	1187	90.8	0.72
Geographic region							
East Anglia	1307	135	10.3	1305	128	9.9	0.66
East Midlands	1307	98	7.5	1305	99	7.6	0.93
West Midlands	1307	118	9.0	1305	120	9.2	0.88
London	1307	134	10.3	1305	152	11.9	0.25
North East	1307	61	4.7	1305	58	4.4	0.79
North West	1307	140	10.7	1305	142	10.8	0.89
South East	1307	177	13.5	1305	176	13.5	0.97
South West	1307	113	8.6	1305	121	9.2	0.58
Yorkshire & Humberside	1307	118	9.0	1305	117	8.9	0.96
Wales	1307	68	5.2	1305	61	4.6	0.53
Scotland	1307	108	8.3	1305	99	7.5	0.52
Northern Ireland	1307	37	2.8	1305	32	2.4	0.55
Personality traits^b^							

	*N* with data	Mean	SD	*N* with data	Mean	SD	
Extraversion	1307	5.8	2.0	1305	5.7	2.1	0.37
Agreeableness	1307	7.0	1.7	1305	7.0	1.7	0.66
Conscientiousness	1307	7.7	1.7	1305	7.7	1.7	0.87
Neuroticism	1307	5.8	2.2	1305	5.8	2.2	0.79
Openness	1307	6.9	1.7	1305	6.8	1.8	0.05

^a^ *p* Values from *t*‐tests for continuous and chi‐squared test for categorical factors compare those with and without risk preference indicators.

^b^ Extraversion, Agreeableness, Conscientiousness, Neuroticism and openness were measured on a scale from 2 to 10.

In order to explore if it is the framing of lottery questions, rather than other imbalanced factors, which leads to different risk preferences in the two versions, three OLS regression models were estimated. As shown in Table 4, the basic regression in column [1] includes age, age squared/100, male, white, and born in the United Kingdom as covariates. Column [2] repeats the same estimation adding socio‐economic variables as controls. In column [3], “big five” personality traits were added. Table 4 shows that coefficients of “Respondent in Version 1” were positively associated with risk indicators at the 1% significance level in all model specifications. This means that, after controlling for variables that could potentially lead to different risk attitudes in the two versions, the version that respondents were randomized to has a significant association with the degree of risk aversion, which indicates a potential framing effect in the indirect approach. Moreover, as mentioned in the methods section, the signs of the indirect risk indicators were reversed in the regressions so a higher score indicates that people are more risk seeking. Thus, the positive coefficients of “Respondent in Version 1” in all models suggest that respondents who were randomized to Version 1 were more risk seeking than those in Version 2, which is consistent with the result from Table 3.

**TABLE 4:** The impact of indirect lottery version on overall indirect risk indicators‐OLS models

[Model]	Dependent variable: Unadjusted indirect risk indicators in the restricted sample		
	[1]	[2]	[3]
Version			
Respondent in Version 1	0.260***	0.289***	0.292***
	(0.002)	(0.001)	(0.001)
Socio‐demographics			
Age (years)	−0.049***	−0.044***	−0.041**
	(0.001)	(0.007)	(0.014)
Age squared/100	0.025	0.021	0.018
	(0.111)	(0.216)	(0.303)
Male	0.106	0.121	0.078
	(0.234)	(0.194)	(0.414)
White	0.115	0.090	0.106
	(0.507)	(0.623)	(0.566)
Born in UK	−0.104	−0.091	−0.065
	(0.525)	(0.599)	(0.708)
Christian		0.043	0.069
		(0.643)	(0.464)
Higher education		0.137	0.129
		(0.141)	(0.171)
Unemployed		−0.166	−0.181
		(0.444)	(0.407)
Household income		0.033	0.035
		(0.303)	(0.282)
Sick or disabled		0.369	0.376
		(0.164)	(0.160)
Married/partnered		−0.098	−0.087
		(0.339)	(0.399)
Own self‐related health (0–10)		0.013	0.013
		(0.662)	(0.666)
Personality traits			
Extraversion			−0.050**
			(0.035)
Agreeableness			0.017
			(0.540)
Conscientiousness			−0.054*
			(0.058)
Neuroticism			−0.033
			(0.174)
Openness			0.018
			(0.487)
Estimators			
Adjusted *R* ^2^	0.037	0.035	0.036
AIC	11,421	10,468	10,469
BIC	11,462	10,549	10,579
Observations	2597	2387	2387

*Note*: ***, **, * represent significance at the 1%, 5%, and 10% significance level, respectively; robust standard errors are shown in parentheses.

However, a simple question that asked whether respondents prefer bananas or oranges showed a different result compared with the complex lottery question. As illustrated in Table 5, the *t*‐test failed to reject the null hypothesis (Pr (*T* > *t*) = 0.32). This means that the proportion of respondents who chose bananas was not significantly different in the two versions, indicating no evidence of a significant framing effect between Versions 1 and 2.

**TABLE 5:** Comparison of choices in two versions using a simple question

	Option 1	N	%	Option 2	N	%
Version 1	Banana	784	59.3	Orange	539	40.7
Version 2	Orange	564	42.6	Banana	759	57.4
Pr (*T* > t)^a^	0.32					

^a^ Pr (*t* > *t*) comes from a two‐sample *t*‐test in estimating the difference in proportions who chose “bananas” in two versions.

### 3.4 Empirical results in predicting risky health behaviors

Table 6 displays the regressions of “drinking five or more alcoholic drinks on one occasion” on our various risk indicators. Three regression models are presented for each of the three approaches (indirect, general direct approach, health specific direct approach). The results of analogous regressions for the other risky health behaviors analyzed (any risky behavior, smoking status, regular smoking, and non‐adherence to taking a full course of antibiotics) are qualitatively similar and presented in the Appendix (Tables A3–A7).

**TABLE 6:** Associations of heavy consumption of alcohol with the direct and indirect risk indicators‐ordered probit models

[Model]	Dependent variable: frequency of drinking five or more alcoholic drinks								
	Indirect approach			Direct approach (general)			Direct approach (health)		
	[1]	[2]	[3]	[1]	[2]	[3]	[1]	[2]	[3]
Risk preferences									
The risk indicator	0.043*	0.047*	0.045*	0.156***	0.157***	0.120***	0.190***	0.183***	0.143***
	(0.025)	(0.026)	(0.026)	(0.026)	(0.027)	(0.030)	(0.026)	(0.027)	(0.028)
Socio‐demographics									
Age (years)	0.024***	0.030***	0.040***	0.027***	0.033***	0.042***	0.021***	0.027***	0.036***
	(0.008)	(0.009)	(0.009)	(0.008)	(0.009)	(0.009)	(0.008)	(0.009)	(0.009)
Age squared/100	−0.043***	−0.048***	−0.058***	−0.045***	−0.050***	−0.059***	−0.037***	−0.043***	−0.053***
	(0.009)	(0.010)	(0.010)	(0.009)	(0.010)	(0.010)	(0.009)	(0.010)	(0.010)
Male	0.433***	0.382***	0.368***	0.383***	0.336***	0.338***	0.386***	0.338***	0.341***
	(0.048)	(0.050)	(0.052)	(0.049)	(0.051)	(0.053)	(0.048)	(0.051)	(0.053)
White	−0.073	−0.058	−0.076	−0.040	−0.024	−0.051	−0.059	−0.042	−0.062
	(0.099)	(0.107)	(0.107)	(0.098)	(0.106)	(0.106)	(0.099)	(0.107)	(0.107)
Born in UK	0.328***	0.349***	0.376***	0.353***	0.370***	0.391***	0.313***	0.332***	0.356***
	(0.094)	(0.100)	(0.098)	(0.093)	(0.099)	(0.098)	(0.093)	(0.099)	(0.098)
Christian		−0.052	−0.069		−0.048	−0.061		−0.045	−0.064
		(0.051)	(0.051)		(0.051)	(0.051)		(0.050)	(0.051)
Higher education		−0.070	−0.077		−0.085*	−0.085*		−0.080	−0.083*
		(0.049)	(0.050)		(0.050)	(0.050)		(0.050)	(0.050)
Unemployed		−0.024	−0.079		−0.019	−0.077		−0.026	−0.074
		(0.117)	(0.115)		(0.119)	(0.116)		(0.117)	(0.115)
Household income		0.051***	0.036**		0.042**	0.033**		0.049***	0.037**
		(0.017)	(0.017)		(0.017)	(0.017)		(0.016)	(0.016)
Sick or disabled		−0.206	−0.237		−0.159	−0.205		−0.160	−0.198
		(0.162)	(0.167)		(0.163)	(0.168)		(0.162)	(0.167)
Married/partnered		−0.105*	−0.081		−0.105*	−0.084		−0.106**	−0.085
		(0.054)	(0.054)		(0.054)	(0.054)		(0.054)	(0.054)
Own self‐related health (0–10)		−0.042***	−0.040***		−0.045***	−0.039**		−0.035**	−0.034**
		(0.015)	(0.015)		(0.015)	(0.015)		(0.015)	(0.015)
Personality traits									
Extraversion			0.104***			0.091***			0.097***
			(0.014)			(0.015)			(0.014)
Agreeableness			−0.013			−0.013			−0.009
			(0.016)			(0.016)			(0.016)
Conscientiousness			−0.087***			−0.089***			−0.073***
			(0.016)			(0.016)			(0.017)
Neuroticism			−0.003			0.008			0.001
			(0.014)			(0.014)			(0.014)
Openness			0.006			−0.001			0.006
			(0.014)			(0.014)			(0.014)
Estimators									
Pseudo *R* ^2^	0.033	0.034	0.050	0.038	0.040	0.053	0.041	0.042	0.054
Log pseudo‐likelihood	−2998	−2783	−2737	−2980	−2766	−2729	−2972	−2760	−2725
AIC	6015	5599	5518	5980	5566	5503	5963	5555	5493
BIC	6073	5695	5642	6037	5662	5627	6020	5650	5617
Observations	2248	2078	2078	2248	2078	2078	2248	2078	2078

*Note*: There are five degrees for the frequency of drinking five or more alcoholic drinks: never, less than once a month, 1–3 times a month, once or twice a week and three or more times a week. These are measured on a scale from 1 to 5. Coefficients of standardized risk indicators were reported. ***, **, * represent significance at the 1%, 5%, and 10% significance level, respectively; robust standard errors are shown in parentheses.

Regression models were constructed the same way as the regressions in which we tested for framing effects in Section 3.3. Model 3 was preferred according to the value of AIC and BIC in all three cases (i.e., indirect, direct [general], and direct [health] regressions). Coefficients and significance levels suggest that the results of associations of risk indicators with risky behaviors are robust to different model specifications.

**FIGURE 4:**
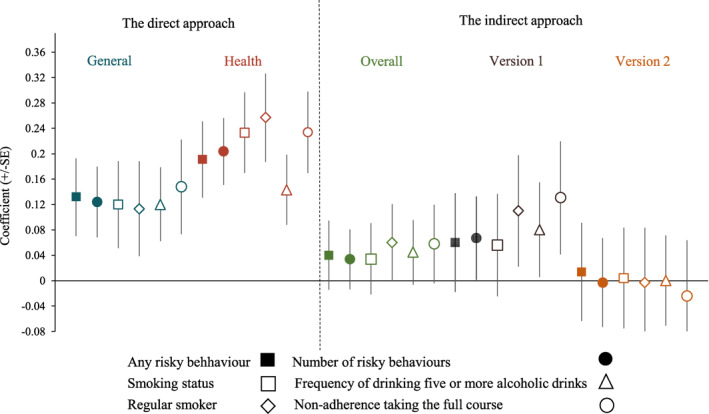
Comparison between different risk indicators using regression models for different health behaviors

Figure 4 compares the ability of risk measures elicited using the indirect versus direct approaches, to predict each of the risky health behaviors, using Model 3 (which is preferred according to AIC and BIC). All risk measures were standardized. The direction of coefficients indicates that people who are deemed more risk seeking by the risk measures are more likely to have risky health behaviors, such as being a regular smoker. The coefficient estimates from the direct approach are positively statistically significant at the 1% level in all regression models, whereas the coefficient estimates of adjusted risk indicators from the indirect approach are significant at most at the 10% level (both using the preferred Model 3). The robustness check which further controlled for Health State A and B in Model 3 showed similar results (Figure A3).

In the direct approach, coefficients of risk indicators had a stronger association with risky health behaviors when elicited in the health domain than in the more general domain. Thus, direct risk questions based on the health domain more strongly suggest that being risk seeking is associated with risky health behaviors. Moreover, models in the health domain have higher pseudo *R* ^2^ and lower values of AIC and BIC than those in the general domain in all cases (Tables 6 and A3–A7). This indicates that the direct risk question based on the health domain can capture more information about risky health behavior, and can predict risky health behaviors better, than more general direct risk questions.

In the indirect approach, coefficient estimates from Version 1 performed better than the coefficient estimates of adjusted risk indicators across two versions and those from Version 2. Importantly, the coefficient estimates in Version 1 are similar to those from the direct approach in the general domain when the dependent variables are regular smoker, frequency of drinking five or more alcoholic drinks and non‐adherence taking the full course, indicating that risk indicators elicited from Version 1 are stronger in predicting risky health behaviors compared with other risk indicators in the indirect approach.

Thus, we analyzed whether framing the gambles differently leads to significant differences among risk indicators elicited from the two versions in the indirect approach. The same three regression models were used in which the dependent variables are the six indicators derived from three risky health behaviors, and the independent variables include risk indicators in two versions and other control variables.

As illustrated in Table 7, it is noteworthy that the coefficient estimates of risk indicators in the two versions are very different across the six dependent variables. The coefficients of risk indicators from Version 1 (gamble on the left‐hand side) are statistically significant at the 5% level when the dependent variables are number of risky behaviors, being a regular smoker and frequency of drinking five or more alcoholic drinks. The predictive power of risk indicators is especially strong in Version 1 where the dependent variable is non‐adherence to taking the full course of antibiotics prescribed. In contrast, the coefficients of risk indicators in Version 2 (gamble on the right‐hand side) are not significant regardless of what the dependent variable is or what the regression model is, indicating a difference in the predictive power of the risk indicators from the two versions. Although the risk indicators do not have predictive power in Version 1 where the dependent variables are any risky behavior and smoking status, the point estimates are consistent with the evidence from the other four dependent variables that strongly suggests that risk indicators from Version 1 can better predict risky behaviors that those from Version 2.

**TABLE 7:** Predictive power of the indirect risk indicators in two versions

Dependent variable	Coefficients in Version 1 (gamble on the left) [model]			Coefficients in Version 2 (gamble on the right) [model]		
	[1]	[2]	[3]	[1]	[2]	[3]
Summary measures of risky health behaviors						
Any risky behavior	0.051	0.059	0.060	0.006	0.003	0.014
	(0.037)	(0.039)	(0.040)	(0.037)	(0.039)	(0.040)
Number of risky behaviors	0.063**	0.067**	0.067**	−0.012	−0.010	−0.003
	(0.032)	(0.033)	(0.034)	(0.033)	(0.035)	(0.036)
Specific risky health behaviors						
Smoking status	0.060	0.059	0.056	−0.001	−0.001	0.004
	(0.038)	(0.041)	(0.041)	(0.038)	(0.040)	(0.040)
Regular smoker	0.106**	0.108**	0.110**	−0.008	−0.010	−0.003
	(0.041)	(0.044)	(0.045)	(0.041)	(0.043)	(0.044)
Frequency of drinking five or more alcoholic drinks	0.080**	0.090**	0.080**	0.004	−0.004	0.000
	(0.035)	(0.037)	(0.038)	(0.035)	(0.036)	(0.036)
Non‐adherence to taking a full course of antibiotics	0.130***	0.138***	0.131***	−0.008	−0.020	−0.024
	(0.042)	(0.045)	(0.045)	(0.041)	(0.044)	(0.045)

*Note*: Coefficients of standardized risk indicators are reported; probit regression for any risky behavior and regular smoker, ordered probit regressions for other risky health behaviors. ***, **, * represent significance at the 1%, 5%, and 10% significance level, respectively; robust standard errors are shown in parentheses.

Additionally, most covariates had similar predictive power in the two versions, such as being “male”, “Christian,” “unemployment,” and “higher education” (shown in Tables A8–A13).

## 4 SENSITIVITY ANALYSIS

As discussed above, the indirect approach can only be analyzed using the restricted sample. However, the robustness of analyses may be affected by sample selection bias. To check this, regression models were estimated in both the full sample and restricted sample using the direct approach, using the preferred Model 3, so that results could be compared in both samples. As illustrated in Table 8, the coefficients of risk indicators are significant at the 1% level in the full sample and are consistent in the full and restricted samples. Control variables such as being male, having higher education and extraversion are also consistent in the two samples, indicating that use of the restricted sample should not substantially affect the generalizability of the results. The analogous results for any risky behavior, number of risk behaviors, smoking, and non‐adherence to antibiotics are broadly similar and deferred to Appendices A14–A18.

**TABLE 8:** Association between heavy consumption of alcohol and direct risk indicators in the full and restricted samples‐ordered probit models

	Dependent variable: frequency of drinking five or more alcoholic drinks			
	General		Health	
	Full sample	Restricted sample	Full sample	Restricted sample
Risk preferences				
The risk indicator	0.125***	0.120***	0.172***	0.143***
	(0.024)	(0.030)	(0.022)	(0.028)
Socio‐demographics				
Age (years)	0.043***	0.042***	0.039***	0.036***
	(0.007)	(0.009)	(0.007)	(0.009)
Age squared/100	−0.062***	−0.059***	−0.056***	−0.053***
	(0.008)	(0.010)	(0.008)	(0.010)
Male	0.366***	0.338***	0.359***	0.341***
	(0.043)	(0.053)	(0.043)	(0.053)
White	−0.103	−0.051	−0.112	−0.062
	(0.079)	(0.106)	(0.079)	(0.107)
Born in UK	0.396***	0.391***	0.368***	0.356***
	(0.073)	(0.098)	(0.073)	(0.098)
Christian	−0.021	−0.061	−0.023	−0.064
	(0.042)	(0.051)	(0.042)	(0.051)
Higher education	−0.069*	−0.085*	−0.071*	−0.083*
	(0.041)	(0.050)	(0.041)	(0.050)
Unemployed	−0.020	−0.077	−0.021	−0.074
	(0.093)	(0.116)	(0.095)	(0.115)
Household income	0.021*	0.033**	0.024*	0.037**
	(0.013)	(0.017)	(0.013)	(0.016)
Sick or disabled	−0.079	−0.205	−0.064	−0.198
	(0.133)	(0.168)	(0.131)	(0.167)
Married/partnered	−0.040	−0.084	−0.048	−0.085
	(0.045)	(0.054)	(0.045)	(0.054)
Own self‐rated health (0–10)	−0.030***	−0.039**	−0.028**	−0.034**
	(0.011)	(0.015)	(0.011)	(0.015)
Personality traits				
Extraversion	0.097***	0.091***	0.103***	0.097***
	(0.012)	(0.015)	(0.012)	(0.014)
Agreeableness	−0.017	−0.013	−0.014	−0.009
	(0.013)	(0.016)	(0.013)	(0.016)
Conscientiousness	−0.087***	−0.089***	−0.069***	−0.073***
	(0.013)	(0.016)	(0.014)	(0.017)
Neuroticism	0.001	0.008	−0.007	0.001
	(0.012)	(0.014)	(0.012)	(0.014)
Openness	0.002	−0.001	0.010	0.006
	(0.012)	(0.014)	(0.012)	(0.014)
Observations	3118	2078	3118	2078

*Note*: Coefficients of standardized risk indicators were reported; ***, **, * represent significance at the 1%, 5% and 10% significance level, respectively; robust standard errors are shown in parentheses.

## 5 DISCUSSION

This study contributes evidence on eliciting risk attitudes using a direct approach and a novel lottery‐based indirect approach. In contrast to Massin et al. (2018) which found that a direct scale approach and health lottery approach performed similarly, our results show that risk indicators from the direct approach are significantly associated with all risky health behaviors, while those in our indirect approach are weakly associated with some of the risky behaviors, indicating that the direct approach may elicit risk indicators that are better predictors of risky health behaviors.^13^

We found that the direct risk question based on the health domain may predict risky health behaviors better than more general direct risk questions. This is consistent with evidence that asking people “willingness to take risks” questions in a specific domain could better predict corresponding behaviors (Dohmen et al., 2011; Massin et al., 2018).

An important finding in our study is the presence and, especially, the consequence of framing effects in the indirect approach. We found consistent results in all models that risk indicators elicited using Version 1 (gamble on the left‐hand side) had stronger predictive power for predicting risky health behaviors than those in Version 2 (gamble on the right‐hand side). This novel result suggests that seemingly innocuous differences in the way lottery elicitation questions are framed may have serious consequences for the ability of the resulting risk indicators to predict risky behaviors.

As shown in Tables 3 and 4, respondents randomized to Version 1 appeared to be more risk seeking, as the risk indicators in Version 1 were lower than those in Version 2. This indicates that they preferred choosing an option with uncertainty that was labeled Option 1. In contrast, risk indicators were higher in Version 2, which indicates that respondents in Version 2 appeared to be more risk averse. This also means that respondents were more likely to choose a certain option labeled Option 1. Thus, framing led to a bias in respondents' decisions. Although it is difficult to unravel which version yields risk preference estimates closer to “true” preferences, the fact that Option 1 was consistent with the direct method in terms of prediction of risky health behavior suggests it may be preferable.

Respondents may be biased toward choosing Option 1 no matter what outcome is presented in the options. This is consistent with the evidence of primacy effects (Anderson & Barrios, 1961; Asch, 1946) and left‐side selection bias observed in surveys (Maeda, 2015; Nicholls et al., 2006), which emphasizes that people may be biased toward choosing options that are shown earlier in a list or shown on the left‐hand side. This may be explained by *satisficing*, a possible behavior in cognitively demanding survey questions in which people may seek a satisfactory option rather than an optimal option (Krosnick, 1991). Respondents may put less effort into understanding questions and may choose an option carelessly, leading to weak satisficing in the decision‐making questions (Simon, 1957), which is likely to bias the process for choosing an option. Respondents, especially weak satisfiers, would choose the first option as a proper and safe answer (Krosnick & Alwin, 1987; Marsden & Wright, 2010). Comparing questions of eliciting risk preferences with choosing a preferred fruit in the survey, the latter question is less cognitively demanding and is therefore easier for people to answer, while the former question is more complex. People may seek a safe answer in more cognitively demanding lottery questions, leading to framing effects in our indirect lottery approach. In contrast, choices from a simple question are less cognitively demanding. People make choices under their intrinsic preference rather than overthinking the question, hence the framing of questions is less likely to lead to biased choices. This is consistent with the findings that simplification can reduce cognitive demands (Reyna & Brainerd, 1991).

The results of our study may have implications for approaches to eliciting risk attitudes. The direct approach, in which people are asked for their willingness to take risks, generated risk indicators that were better at predicting risky health behaviors than those elicited in the indirect approach. Compared with the indirect approach, the better performance of the direct approach might be explained both by the relative simplicity of the scale‐based design (with relatively low numeracy or health literacy requirements needed by respondents), and the much lower scope for framing effects (Borghans et al., 2008).

However, the weaker predictive power of risk indicators in the indirect approach was partly caused by combining the risk indicators from the two versions, rather than the approach per se. Risk indicators from Version 1 (gamble on the left‐hand side) had much more power in predicting risky behaviors than those from Version 2 (gamble on the right‐hand side). Risk indicators from Version 2 may have diluted the predictive power of the adjusted indirect risk indicators, contributing to the result that the indirect approach performed less well than the direct approach at predicting risky health behaviors.^14^ Indeed the indirect approach using Version 1 performed similarly to the direct approach in the general domain in predicting risky health behaviors. The marginal effects of the indirect risk indicators from Version 1 had similar magnitudes as those from the direct approach in the general domain on regular smoking, frequency of taking five or more alcoholic drinks, and non‐adherence to taking a full course of antibiotics. In contrast, the corresponding marginal effects of the overall indirect risk indicators and the risk indicators from Version 2 are relatively small.^15^ Moreover, an advantage of the indirect lottery approach over the direct approach, is that it elicits attitudes toward risks directly from individuals' choices, based on a standard model of decisions under uncertainty^16^ (Massin et al., 2018). Compared with direct scale tasks, the lottery approach is preferable when information about the curvature of utility functions is needed (Andersen et al., 2008). While the lottery approach in monetary terms has been widely used in elicitation of risk preferences, using health outcomes as payoffs is still more novel, and there is evidence that domain‐specific direct questions about risks have the most predictive power (Dohmen et al., 2011). This suggests that an indirect approach with health outcomes may also better elicit attitudes toward risks with health than more traditional indirect approaches with financial outcomes. This indirect approach is especially useful when estimating the parameters of utility functions in the health context.

Reducing the potential for framing effects in the indirect lottery method may improve measures of attitudes toward risk. For instance, a gamble option may be put on the left‐hand side or in the first order when using lottery elicitation methods, as our evidence shows that risk indicators elicited from Version 1 (gamble on the left‐hand side) could predict risky health behaviors better than those in Version 2 (gamble on the right‐hand side). Moreover, the results of the question about choosing the preferred fruit indicates that there was no framing effect between the two versions, which suggests that developing lottery questions in a less complex way might reduce framing effects. Because of the potential for improvements to indirect approaches, and their lottery‐based advantages, future research could further develop indirect approaches.

The strong associations between risk preferences and risky health behaviors may have important implications for the development of public health interventions. Our results indicate that people who are more risk seeking are more likely to have risky health behaviors.^17^ Single risk questions, with high predictive power across a range of health behaviors, could potentially be incorporated into household surveys in place of multiple health behavior questions. Health interventions to modify risky behaviors, such as smoking and drinking alcohol, could then be targeted simply toward people who are more risk seeking with their health in general. For example, public information campaigns to discourage excessive drinking could be displayed on websites or on television channels that are more likely to be watched by people who are risk takers. Audience profiles for media are often well known with respect to age, gender, and other socio‐demographic characteristics. Inclusion of questions that better capture risk preferences in, for example, household surveys could lead to improved understanding of the media consumption of people who are likely to be risk‐seekers in multiple health domains.

The study has several limitations. A key limitation is that risk preferences could only be constructed for the restricted sample (65% of the full sample), due to the inability of 1388 respondents (35% of the full sample) to recognize that Health State A is better than Health State B, yet worse than Full Health. However, it is possible that this may reflect an inherent limitation in health state based risk preference elicitation methods. This feature of our indirect method suggests that, even if framed in way that results in high predictive ability, such indirect approaches may not be a preferred tool if a substantial proportion of respondents are unable to provide valid answers to the lottery questions. For the 35% of respondents who did not understand the lottery question, fewer had higher education than those in the restricted sample.^18^ Hence, without amendments, our indirect approach is not simple enough to elicit risk preferences for a fully representative general population. Future research could explore using lottery approaches with health outcomes in a simpler way, or using lottery approaches for a targeted population with a higher educational level. Another limitation is that, as a survey study, we were only able to assess self‐reported behavior in a hypothetical context, not actual behavior. Risk attitudes elicited from hypothetical contexts have sometimes been found to differ from those elicited using real rewards (Arrieta et al., 2017; Sanou et al., 2018), but several other studies have not found any differences (Abdellaoui et al., 2013; Bardsley et al., 2009; Cohen et al., 2020). Furthermore, the self‐reported questions about risk attitudes have been found to predict actual behavior well (Dohmen et al., 2011). In addition, there are potentially social desirability biases in the survey, where respondents give a socially desirable answer, rather than an accurate response, to sensitive questions such as risky health behaviors (Davis et al., 2010). However, the web‐based nature of the survey design, with no interviewer present, is likely to reduce the risk of any such bias (Krumpal, 2013). Finally, only respondents with access to computers who were willing to finish the questionnaire could be included in the sample. However, the sample is broadly representative of the UK population, providing some confidence for the generalizability of the results.^19^

Despite these limitations, this study identifies a potential framing effect in the indirect approach in the health domain. We found that an apparently small change to the way questions are framed may lead to large differences in results, particularly in regression analyses with risk indicators as explanatory variables. However, little research has focused on evaluating attitudes to risks using the indirect approach with health outcomes. Future research could try to find ways to design approaches that are less vulnerable to framing effects.

## CONFLICT OF INTEREST

The authors declare that they have no conflict of interest.

## Supporting information

Supporting Information S1

## Data Availability

The data that support the findings of this study are available from the corresponding author upon reasonable request.

## ENDNOTES

^1^
This finding extended to other domains. For example, a direct question about willingness to take financial risks was the best predictor of investment in stocks, and a direct question about willingness to take risks in one's career was the best predictor of self‐employment.

^2^
Roope et al. (2018) initially developed the method to create an indicator of risk‐aversion in an exploration of attitudes toward antibiotic use. To the best of our knowledge, this study is the first use of the measure in the economics literature.

^3^
Compared with Massin et al. (2018), which elicited both time preference and risk preferences using the lottery approach with health outcomes, we focused only on how people's risk preference elicited from the indirect approach predict risky health behaviors.

^4^
The survey was developed to also support several studies. Thus, it also contained questions not directly related to this study. The full survey is provided in the Supplementary materials.

^5^
Big Five personality traits is a well‐established personality taxonomy in which illustrates that personality is described in five dimensions: Neuroticism, Extraversion, Openness, Agreeableness and Conscientiousness. See Goldberg (1990) for details.

^6^
For further details on the development of this lottery approach, including its piloting and application in the context of attitudes toward antibiotic use see Roope et al. (2018).

^7^
2612 (65.3%) respondents were included in the restricted sample used to construct risk preference data; 34.7% respondents were excluded as they did not recognize that Health State A was better than Health State B but worse than Full Health.

^8^
Utilities here are interpreted as the scores that respondents assigned to the health states on a scale from 0 to 10 in the survey.

^9^
AIC and BIC measure the quality of a statistical model based on the goodness of fit and the parsimony of the model. A key difference between AIC and BIC is that BIC gives more penalty for additional parameters than AIC. In other words, BIC puts more emphasis on the parsimony of a model. See Akaike (1974) and Schwarz (1978).

^10^
We used a link test, developed by Pregibon (1979), based on work by Tukey (1949).

^11^
There are three smoking statuses in the survey, “non‐smoker,” “occasional smoker” and “regular smoker.” “Regular smoker” is a binary variable which equals 1 if the respondent smokes every day and equals zero otherwise. There are five categories of “frequency of drinking five or more alcoholic drinks”: “never,” “less than once a month,” “1–3 times a month,” “once or twice a week” and “three or more times a week.”. Regular drinker is defined as respondents drinking once or twice a week, or three or more times a week. Occasional drinker is defined as respondents drinking less than once a month, or 1–3 times a month. Non‐drinker is defined as respondents who never drink alcohol. There are four categories under ‘non‐adherence to taking a full course of antibiotics’: “definitely,” “probably,” “probably not” and “definitely not.”

^12^
To create an overall risk indicator across two versions in the restricted sample using Equations (3), (4), (5) in Section 2, we performed a two‐sample *t*‐test to compare the difference between the indirect risk indicators from the two versions. *d* in Equations (3) and (4) equals 0.27 (Table A1).

^13^
One plausible explanation of these contrasting results is the different samples used in the studies. There are a variety of reasons why results for such a select population as GPs (sample in Massin et al., 2018) are likely to differ from those of a more general population (sample in this study). For example, GPs are more educated than a general population and more health literate. For these and other reasons, they may also potentially be affected to a lesser extent by framing effects in elicitation methods.

^14^
In the pooled regression estimates across Versions 1 and 2 (Tables 6 and Tables A8‐A13), the combined risk indicators had less predictive power for risky health behaviors than in the Version 1 only regressions (Table 7), despite the sample being twice the size; but greater predictive power than in the Version 2 only regression (Table 7). It is possible that risk indicators from the indirect approach might have predicted risky health behaviors as well as the direct risk indicators if the risk estimates in the full sample had been elicited using the Version 1 method (gamble on the left).

^15^
The marginal effects of all risky health behaviors using preferred model [3] are available in Table A19.

^16^
We have based it on expected utility theory, the most standard model, but the approach could potentially be extended to other models, for example, by incorporating a reference health level.

^17^
While not the focus of this study, we also note in passing some interesting independent associations between Big Five personality traits and risky health behaviors. Higher levels of Extraversion and lower levels of Conscientiousness were associated with heavy consumption of alcoholic drinks, while higher levels of Extraversion and Agreeableness were associated with smoking behavior. Extraversion has previously been shown to be important in explaining the role for both smoking and drinking behavior (Abe et al., 2019; Adan et al., 2017), while Agreeableness has been found to better predict drinking than smoking (Bessey, 2018). There is also evidence that low Conscientiousness (Abe et al., 2019; Cheng & Furnham, 2016) and high levels of Openness (Zvolensky et al., 2015) are predictors of smoking behavior. Interestingly, low Agreeableness and Conscientiousness were the traits most predictive of non‐adherence to taking a full course of antibiotics.

^18^
Among 1388 respondents who were excluded, 221 (15.9%) respondents did not realize that Health State A and B were worse than full health, so they rated at least one of Health States A and B as being equal to 10. 1167 (84.1%) respondents realized that A and B are not full health, but did not realize that Health State A is better than B. That is, they rated Health State A as equal to B, or worse than B. The most plausible explanation for the inability of respondents to correctly perceive Health State A as better than B is that they simply did not have sufficient health literacy to do so. These results suggest that future studies that plan to employ health outcomes in indirect elicitation approaches similar to those here should take considerable care when designing the health states.

^19^
An exception is that the level of higher education was relatively high, though being an online study, this is perhaps not unexpected.
