# Supplementary material for: Eliciting risk preferences that predict risky health behavior: A comparison of two approaches
Source: Health Econ. 2022 Feb 22;31(5):836–58. doi: 10.1002/hec.4486 (PMC9305924; doi:10.1002/hec.4486)
Supplement: Supplementary file 1 — Supporting Information S1 [file HEC-31-836-s001.docx]

**SUPPLEMENTARY MATERIALS: FOR ONLINE PUBLICATION ONLY**

# Appendix 1. Survey Instrument

*A questionnaire to better understand your views on different health conditions*

Researchers and doctors at the University of Oxford are carrying out a survey of 4,000 adults in the United Kingdom to learn more about attitudes to some common health conditions, and to the use of antibiotics. We are very interested in learning more about your views. During the survey we will provide you with some information about how best to treat some common health conditions, and about antibiotics. We will then ask you some questions in response to this information.

Health can affect, and be affected by, many different aspects of life. The questionnaire also, therefore, asks a variety of questions about yourself – for example, your background, your income, and even your personality. Some of these questions may not seem to have obvious links to health, but it is important that we ask them, as your answers might help us to uncover some surprising and useful insights that could lead to improvements in healthcare.

The data you provide will be anonymised by Survey Sampling International (SSI) before sending it to researchers at the University of Oxford for academic research use. SSI will not share any of your personal data with the University of Oxford. The anonymised data will be stored on secure networks at the University of Oxford, and archived securely at the end of the project.

If you have any concerns arising from any of the health-related issues raised in the survey, please don’t hesitate to ask to discuss them either with your GP or with another health care professional.

The questionnaire is divided into three sections. In total, it should take around 15 minutes to complete. Some of the questions may require a little thought, but please try to answer them all if you can. There are no right or wrong answers - we are simply interested in your views.

Participation in the survey is voluntary. If you do participate, you may withdraw without giving a reason and without penalty. You can withdraw at any time during the survey by simply closing your browser window. This study has been reviewed by, and received ethics clearance through, the University of Oxford Central University Research Ethics Committee.

*What if there is a problem?*

If you have a concern about any aspect of this project, please speak to Dr. Laurence Roope (01865 617 913) [or Dr. Sarah Wordsworth (01865 289 268)] who will do their best to answer your query. The researcher should acknowledge your concern within 10 working days and give you an indication of how he/she intends to deal with it. If you remain unhappy or wish to make a formal complaint, please contact the chair of the Research Ethics Committee at the University of Oxford (Chair, Medical Sciences Inter-Divisional Research Ethics Committee; Email: [ethics@medsci.ox.ac.uk](https://owa.nexus.ox.ac.uk/owa/redir.aspx?SURL=jKy8rZL7Ge6U2tbkLs_TBesF87q0J185mBLEO-lvlXw7fJN5qz_UCG0AYQBpAGwAdABvADoAZQB0AGgAaQBjAHMAQABtAGUAZABzAGMAaQAuAG8AeAAuAGEAYwAuAHUAawA.&URL=mailto%3aethics%40medsci.ox.ac.uk); Address: Research Services, University of Oxford, Wellington Square, Oxford OX1 2JD). The chair will seek to resolve the matter in a reasonably expeditious manner.

If you would like to take the survey, and are over 18 years old, please click on the box below marked ‘Yes’. If you no longer wish to take the survey, or are under 18 years old, please click on the box marked ‘No’.

Yes No


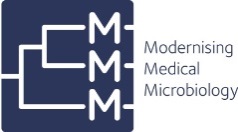

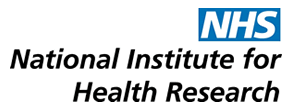

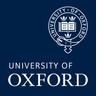


**This information is displayed at the start of the survey as a stand-alone webpage.**

Throughout the following questions, except where instructed otherwise, please indicate your answer by entering an ‘X’ in the one box which you feel best describes your answer. Certain questions will need to be answered in a slightly different way, such as by entering a number in a box. In these cases, the instructions will be clearly indicated in the question. Although we would like you to answer all questions, it is not compulsory to do so.

Section 1

We would like to begin the questionnaire by asking you a few questions about yourself.

1. Are you

Male Female Prefer not to say

2. How old were you on your last birthday? *Please indicate your answer by entering a number in the box below:*

years

3. Which of the following apply to your current situation? *Please enter ‘X’ in the boxes beside all which apply.*

|  | Employed full-time |
| --- | --- |
|  | Employed part-time |
|  | Self-employed full-time |
|  | Self-employed part-time |
|  | Unemployed |
|  | Retired |
|  | Permanently sick or disabled |
|  | Looking after home or family |
|  | In full-time education |
|  | Other |

4. What is the highest level of education or training you have attained?

|  | GCSEs or ‘O’ Levels or CSEs or Scottish Standard Grade |
| --- | --- |
|  | ‘A’ Levels or ‘AS’ Levels or Scottish Higher Grade |
|  | International Baccalaureate |
|  | Technical or Vocational Qualification |
|  | Transition Year Programme |
|  | Higher Education Certificate or Diploma |
|  | Undergraduate Degree |
|  | Professional Qualification (comparable to undergraduate degree) |
|  | Postgraduate Certificate or Diploma |
|  | Postgraduate Degree |
|  | Doctorate |
|  | Other |
|  | None |

5. Are you currently married, in a civil partnership, or living with a partner?

Yes No

6. Gross HOUSEHOLD income combines your income with that of your partner or any other household members with whom you share financial responsibilities BEFORE any taxes or deductions.

What is your gross annual household income?

|  | Up to £10,000 |
| --- | --- |
|  | £10,000 to £19,999 |
|  | £20,000 to £29,999 |
|  | £30,000 to £39,999 |
|  | £40,000 to £49,999 |
|  | £50,000 to £74,999 |
|  | £75,000 to £99,999 |
|  | £100,000 or more |
|  | Prefer not to say |

7a. Do you have any dependent children who live with you? (By ‘dependent’ children, we mean those who are not yet financially independent).

*Yes No*

***[If no, go to question 8].***

7b. How many dependent children do you have who live with you? *Please indicate your answer by entering a number in the box below:*

7c. How old is your youngest dependent child who lives with you?* *Please indicate your answer by entering a number in one of the boxes below. If your child is less than 2 years old, please answer in months; otherwise answer in years.*

*Years Months*

*** *[If respondent indicates that they have only one dependent child who lives with them, amend text to “And how old is this child”?]***

8. Including yourself, how many adults live in your household? (This refers to *all* adults, including any children aged 18 or over, who live with you. Please also include any children aged 16 or 17 who live with you, if they are financially independent). *Please indicate your answer by entering a number in the box below:*

9. Were you born in the UK?

*Yes No*

10. How would you describe your ethnicity or background?

|  | White |
| --- | --- |
|  | Mixed / multiple ethnic groups |
|  | Black / African / Caribbean / Black British |
|  | Asian / Asian British |
|  | Other ethnic group |
|  | Prefer not to say |

11. What, if any, is your religion?

|  | No religion |
| --- | --- |
|  | Christianity (Protestant) |
|  | Christianity (Catholic) |
|  | Christianity (Other) |
|  | Islam |
|  | Hinduism |
|  | Sikhism |
|  | Judaism |
|  | Buddhism |
|  | Other religion |
|  | Prefer not to say |

12. Which of the following best describes where you live?

|  | South East |
| --- | --- |
|  | London |
|  | North West |
|  | East of England |
|  | West Midlands |
|  | South West |
|  | Yorkshire and the Humber |
|  | East Midlands |
|  | North East |
|  | Scotland |
|  | Wales |
|  | Northern Ireland |

We would like to conclude this section by asking you a few questions about your lifestyle.

13. Do you smoke tobacco?

*Yes - every day*

*Yes - but not every day*

*No*

***[If respondent chooses one of the first two options, go to question 15].***

14. Have you ever smoked tobacco?

*Yes – I used to smoke every day*

*Yes – I used to smoke, but not every day*

*Yes – but I tried it only once or a few times*

*No*

15. Have you ever tried electronic cigarettes?

*Yes No*

16. How often do you drink alcohol?

*Never*

*Less than once a month*

*1 to 3 times a month*

*Once or twice a week*

*Three or more times a week*

***[If respondent chooses “Never” go to Section 2]***

17. How often do you drink five or more alcoholic drinks on one occasion?

*Never*

*Less than once a month*

*1 to 3 times a month*

*Once or twice a week*

*Three or more times a week*

Section 2

We would like to begin this section by asking you to imagine two health states, which we will call Health State A and Health State B.

***Health State A****: You have*

- *a temperature,*
- *aching muscles,*
- *a headache,*
- *a dry chesty cough,*
- *a sore throat,*
- *and you feel weak*

***Health State B****: You have*

- *a temperature,*
- *chest pain,*
- *night sweats,*
- *a cough that brings up phlegm,*
- *loss of appetite (not wanting to eat),*
- *you feel drained,*
- *and you have lost some weight.*

***[NOTE: Health State A and Health State B to remain on screen for all questions in which they are referred to]***

Drawn below is a scale, where the **best** health state you can imagine is marked **10** (we will call this ‘Full Health’), and the **worst** health state you can imagine is marked **0**.


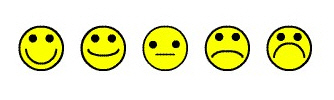

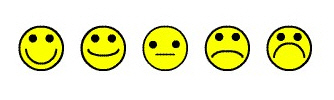

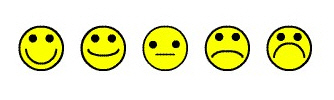

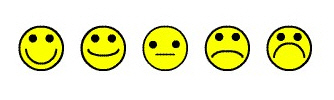

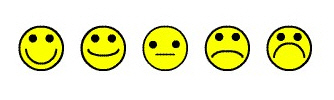


Worst imaginable

health state

Best imaginable

health state

(Full Health)


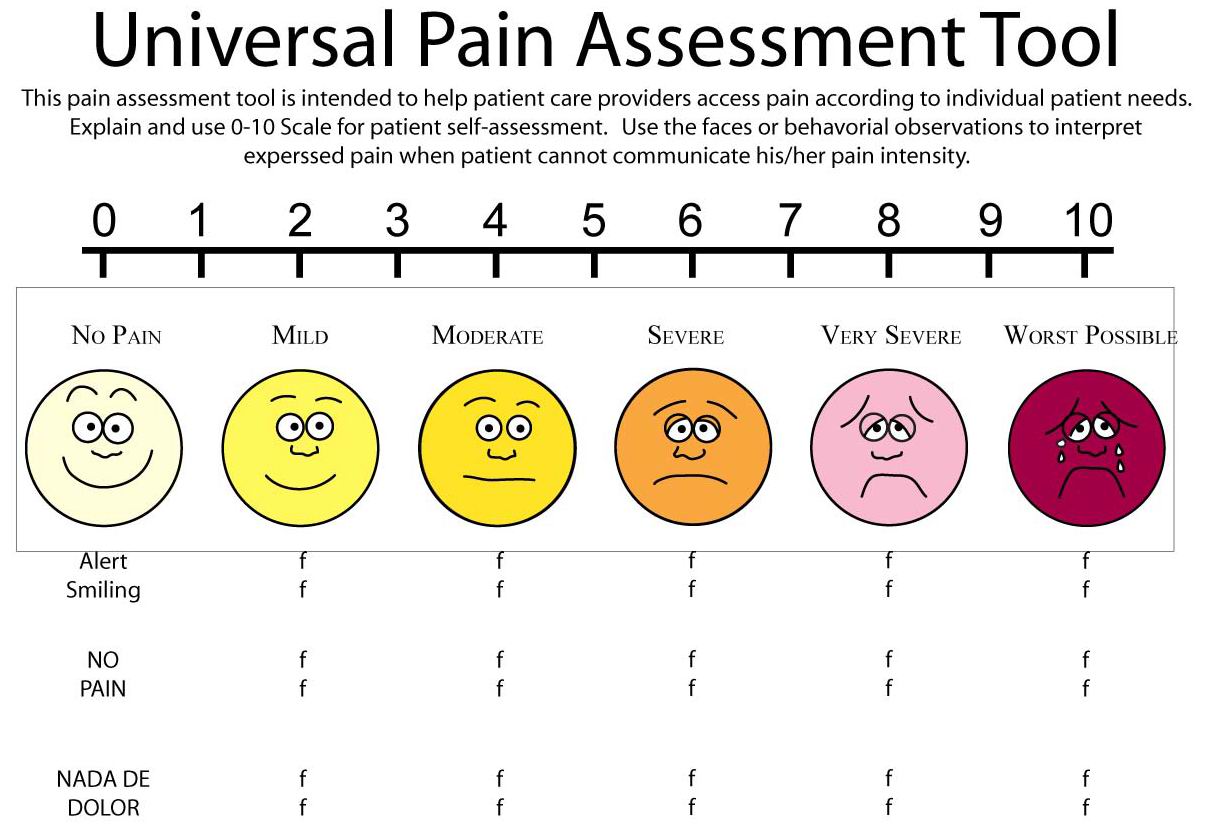


1. How would you rate Health State A on this scale? *Please do so by writing a number between 0 and 10 in the box below.*

|  |
| --- |

2. How would you rate Health State B on this scale? *Please do so by writing a number between 0 and 10 in the box below.*

|  |
| --- |

*3.* How would you rate your own health today on this scale? *Please do this by writing a number between 0 and 10 in the box below.*

|  |
| --- |

*[If the number given to answer Q1 is less than the number given to answer Q2, go to Q7].*

**Stand-alone page to be displayed between Q3 and Q4.**

*The following questions are about the timing of different health states. You have told us that you regard Health State B as being worse than Health State A. However, some people might accept being in severely poor health at some point in the future, if it meant that they could avoid being in moderately poor health at the present time.*

*4. In this question, assume that if either Health State A or B occurs it will last for two weeks. Apart from those two weeks, you will be in Full Health.*

*(a) Which of the following options would you prefer?*

*Option 1: Health State A now (2016)*

| ***Health State A*** | ***Full Health*** | ***Full Health*** | ***Full Health*** | ***Full Health*** | ***Full Health*** | ***Full Health*** | ***Full Health*** | ***Full Health*** | ***Full Health*** |
| --- | --- | --- | --- | --- | --- | --- | --- | --- | --- |
| *2016* | *2018* | *2020* | *2022* | *2024* | *2026* | *2028* | *2030* | *2032* | *2034* |

*or*

*Option 2: Health State B in 10 years’ time (2026)*

| ***Full Health*** | ***Full Health*** | ***Full Health*** | ***Full Health*** | ***Full Health*** | ***Health State B*** | ***Full Health*** | ***Full Health*** | ***Full Health*** | ***Full Health*** |
| --- | --- | --- | --- | --- | --- | --- | --- | --- | --- |
| *2016* | *2018* | *2020* | *2022* | *2024* | *2026* | *2028* | *2030* | *2032* | *2034* |

***Option 1:*** *Health State A now (2016)*

***Option 2:*** *Health State B in 10 years’ time (2026)*

***[If Option 2, go to part (f)]***

*(b) Which of the following options would you prefer?*

*Option 1: Health State A now (2016)*

| ***Health State A*** | ***Full Health*** | ***Full Health*** | ***Full Health*** | ***Full Health*** | ***Full Health*** | ***Full Health*** | ***Full Health*** | ***Full Health*** | ***Full Health*** |
| --- | --- | --- | --- | --- | --- | --- | --- | --- | --- |
| *2016* | *2018* | *2020* | *2022* | *2024* | *2026* | *2028* | *2030* | *2032* | *2034* |

*or*

*Option 2: Health State B in 18 years’ time (2034)*

| ***Full Health*** | ***Full Health*** | ***Full Health*** | ***Full Health*** | ***Full Health*** | ***Full Health*** | ***Full Health*** | ***Full Health*** | ***Full Health*** | ***Health State B*** |
| --- | --- | --- | --- | --- | --- | --- | --- | --- | --- |
| *2016* | *2018* | *2020* | *2022* | *2024* | *2026* | *2028* | *2030* | *2032* | *2034* |

***Option 1:*** *Health State A now (2016)*

***Option 2:*** *Health State B in 18 years’ time (2034)*

***[If Option 1, go to part (j)]***

*(c) Which of the following options would you prefer?*

*Option 1: Health State A now (2016)*

| ***Health State A*** | ***Full Health*** | ***Full Health*** | ***Full Health*** | ***Full Health*** | ***Full Health*** | ***Full Health*** | ***Full Health*** | ***Full Health*** | ***Full Health*** |
| --- | --- | --- | --- | --- | --- | --- | --- | --- | --- |
| *2016* | *2018* | *2020* | *2022* | *2024* | *2026* | *2028* | *2030* | *2032* | *2034* |

*or*

*Option 2: Health State B in 14 years’ time (2030)*

| ***Full Health*** | ***Full Health*** | ***Full Health*** | ***Full Health*** | ***Full Health*** | ***Full Health*** | ***Full Health*** | ***Health State B*** | ***Full Health*** | ***Full Health*** |
| --- | --- | --- | --- | --- | --- | --- | --- | --- | --- |
| *2016* | *2018* | *2020* | *2022* | *2024* | *2026* | *2028* | *2030* | *2032* | *2034* |

***Option 1:*** *Health State A now (2016)*

***Option 2:*** *Health State B in 14 years’ time (2030)*

***[If Option 2, go to part (e)]***

*(d) Which of the following options would you prefer?*

*Option 1: Health State A now (2016)*

| ***Health State A*** | ***Full Health*** | ***Full Health*** | ***Full Health*** | ***Full Health*** | ***Full Health*** | ***Full Health*** | ***Full Health*** | ***Full Health*** | ***Full Health*** |
| --- | --- | --- | --- | --- | --- | --- | --- | --- | --- |
| *2016* | *2018* | *2020* | *2022* | *2024* | *2026* | *2028* | *2030* | *2032* | *2034* |

*or*

*Option 2: Health State B in 16 years’ time (2032)*

| ***Full Health*** | ***Full Health*** | ***Full Health*** | ***Full Health*** | ***Full Health*** | ***Full Health*** | ***Full Health*** | ***Full Health*** | ***Health State B*** | ***Full Health*** |
| --- | --- | --- | --- | --- | --- | --- | --- | --- | --- |
| *2016* | *2018* | *2020* | *2022* | *2024* | *2026* | *2028* | *2030* | *2032* | *2034* |

***Option 1:*** *Health State A now (2016)*

***Option 2:*** *Health State B in 16 years’ time (2032)*

***[Now go to question 5]***

*(e) Which of the following options would you prefer?*

*Option 1: Health State A now (2016)*

| ***Health State A*** | ***Full Health*** | ***Full Health*** | ***Full Health*** | ***Full Health*** | ***Full Health*** | ***Full Health*** | ***Full Health*** | ***Full Health*** | ***Full Health*** |
| --- | --- | --- | --- | --- | --- | --- | --- | --- | --- |
| *2016* | *2018* | *2020* | *2022* | *2024* | *2026* | *2028* | *2030* | *2032* | *2034* |

*or*

*Option 2: Health State B in 12 years’ time (2028)*

| ***Full Health*** | ***Full Health*** | ***Full Health*** | ***Full Health*** | ***Full Health*** | ***Full Health*** | ***Health State B*** | ***Full Health*** | ***Full Health*** | ***Full Health*** |
| --- | --- | --- | --- | --- | --- | --- | --- | --- | --- |
| *2016* | *2018* | *2020* | *2022* | *2024* | *2026* | *2028* | *2030* | *2032* | *2034* |

***Option 1:*** *Health State A now (2016)*

***Option 2:*** *Health State B in 12 years’ time (2028)*

***[Now go to question 5]***

*(f) Which of the following options would you prefer?*

*Option 1: Health State A now (2016)*

| ***Health State A*** | ***Full Health*** | ***Full Health*** | ***Full Health*** | ***Full Health*** | ***Full Health*** | ***Full Health*** | ***Full Health*** | ***Full Health*** | ***Full Health*** |
| --- | --- | --- | --- | --- | --- | --- | --- | --- | --- |
| *2016* | *2018* | *2020* | *2022* | *2024* | *2026* | *2028* | *2030* | *2032* | *2034* |

*or*

*Option 2: Health State B in 2 years’ time (2018)*

| ***Full Health*** | ***Health State B*** | ***Full Health*** | ***Full Health*** | ***Full Health*** | ***Full Health*** | ***Full Health*** | ***Full Health*** | ***Full Health*** | ***Full Health*** |
| --- | --- | --- | --- | --- | --- | --- | --- | --- | --- |
| *2016* | *2018* | *2020* | *2022* | *2024* | *2026* | *2028* | *2030* | *2032* | *2034* |

***Option 1:*** *Health State A now (2016)*

***Option 2:*** *Health State B in 2 years’ time (2018)*

***[If Option 2, go to question 5]***

*(g) Which of the following options would you prefer?*

*Option 1: Health State A now (2016)*

| ***Health State A*** | ***Full Health*** | ***Full Health*** | ***Full Health*** | ***Full Health*** | ***Full Health*** | ***Full Health*** | ***Full Health*** | ***Full Health*** | ***Full Health*** |
| --- | --- | --- | --- | --- | --- | --- | --- | --- | --- |
| *2016* | *2018* | *2020* | *2022* | *2024* | *2026* | *2028* | *2030* | *2032* | *2034* |

*or*

*Option 2: Health State B in 6 years’ time (2022)*

| ***Full Health*** | ***Full Health*** | ***Full Health*** | ***Health State B*** | ***Full Health*** | ***Full Health*** | ***Full Health*** | ***Full Health*** | ***Full Health*** | ***Full Health*** |
| --- | --- | --- | --- | --- | --- | --- | --- | --- | --- |
| *2016* | *2018* | *2020* | *2022* | *2024* | *2026* | *2028* | *2030* | *2032* | *2034* |

***Option 1:*** *Health State A now (2016)*

***Option 2:*** *Health State B in 6 years’ time (2022)*

***[If Option 1, go to part (i)]***

*(h) Which of the following options would you prefer?*

*Option 1: Health State A now (2016)*

| ***Health State A*** | ***Full Health*** | ***Full Health*** | ***Full Health*** | ***Full Health*** | ***Full Health*** | ***Full Health*** | ***Full Health*** | ***Full Health*** | ***Full Health*** |
| --- | --- | --- | --- | --- | --- | --- | --- | --- | --- |
| *2016* | *2018* | *2020* | *2022* | *2024* | *2026* | *2028* | *2030* | *2032* | *2034* |

*or*

*Option 2: Health State B in 4 years’ time (2020)*

| ***Full Health*** | ***Full Health*** | ***Health State B*** | ***Full Health*** | ***Full Health*** | ***Full Health*** | ***Full Health*** | ***Full Health*** | ***Full Health*** | ***Full Health*** |
| --- | --- | --- | --- | --- | --- | --- | --- | --- | --- |
| *2016* | *2018* | *2020* | *2022* | *2024* | *2026* | *2028* | *2030* | *2032* | *2034* |

***Option 1:*** *Health State A now (2016)*

***Option 2:*** *Health State B in 4 years’ time (2020)*

***[Now go to question 5]***

*(i) Which of the following options would you prefer?*

*Option 1: Health State A now (2016)*

| ***Health State A*** | ***Full Health*** | ***Full Health*** | ***Full Health*** | ***Full Health*** | ***Full Health*** | ***Full Health*** | ***Full Health*** | ***Full Health*** | ***Full Health*** |
| --- | --- | --- | --- | --- | --- | --- | --- | --- | --- |
| *2016* | *2018* | *2020* | *2022* | *2024* | *2026* | *2028* | *2030* | *2032* | *2034* |

*or*

*Option 2: Health State B in 8 years’ time (2024)*

| ***Full Health*** | ***Full Health*** | ***Full Health*** | ***Full Health*** | ***Health State B*** | ***Full Health*** | ***Full Health*** | ***Full Health*** | ***Full Health*** | ***Full Health*** |
| --- | --- | --- | --- | --- | --- | --- | --- | --- | --- |
| *2016* | *2018* | *2020* | *2022* | *2024* | *2026* | *2028* | *2030* | *2032* | *2034* |

***Option 1:*** *Health State A now (2016)*

***Option 2:*** *Health State B in 8 years’ time (2024)*

***[Now go to question 5]***

*(j) Is there any far off time in the future at which being in Health State B would be preferable to being in Health State A today?*

*Please indicate your answer by writing a number in the box below. (If you would never prefer Health State B at any point in the future, to Health State A today, please write the letter “N” in the box below, instead of a number).*

*“Rather than Health State A today, I would prefer Health State B, providing it were at least*

*years from now.”*

***[If “N”, go to Q6.]***

*5. In this question, again assume that if either Health State A or B occurs it will last for two weeks. Apart from those two weeks, you will be in Full Health.*

*(a) Which of the following options would you prefer?*

*Option 1: Health State A in 2 years’ time (2018)*

| ***Full Health*** | ***Health State A*** | ***Full Health*** | ***Full Health*** | ***Full Health*** | ***Full Health*** | ***Full Health*** | ***Full Health*** | ***Full Health*** | ***Full Health*** | ***Full Health*** |
| --- | --- | --- | --- | --- | --- | --- | --- | --- | --- | --- |
| *2016* | *2018* | *2020* | *2022* | *2024* | *2026* | *2028* | *2030* | *2032* | *2034* | *2036* |

*or*

*Option 2: Health State B in 12 years’ time (2028)*

| ***Full Health*** | ***Full Health*** | ***Full Health*** | ***Full Health*** | ***Full Health*** | ***Full Health*** | ***Health State B*** | ***Full Health*** | ***Full Health*** | ***Full Health*** | ***Full Health*** |
| --- | --- | --- | --- | --- | --- | --- | --- | --- | --- | --- |
| *2016* | *2018* | *2020* | *2022* | *2024* | *2026* | *2028* | *2030* | *2032* | *2034* | *2036* |

***Option 1:*** *Health State A in 2 years’ time (2018)*

***Option 2:*** *Health State B in 12 years’ time (2028)*

***[If Option 2, go to part (f)]***

*(b) Which of the following options would you prefer?*

*Option 1: Health State A in 2 years’ time (2018)*

| ***Full Health*** | ***Health State A*** | ***Full Health*** | ***Full Health*** | ***Full Health*** | ***Full Health*** | ***Full Health*** | ***Full Health*** | ***Full Health*** | ***Full Health*** | ***Full Health*** |
| --- | --- | --- | --- | --- | --- | --- | --- | --- | --- | --- |
| *2016* | *2018* | *2020* | *2022* | *2024* | *2026* | *2028* | *2030* | *2032* | *2034* | *2036* |

*or*

*Option 2: Health State B in 20 years’ time (2036)*

| ***Full Health*** | ***Full Health*** | ***Full Health*** | ***Full Health*** | ***Full Health*** | ***Full Health*** | ***Full Health*** | ***Full Health*** | ***Full Health*** | ***Full Health*** | ***Health State B*** |
| --- | --- | --- | --- | --- | --- | --- | --- | --- | --- | --- |
| *2016* | *2018* | *2020* | *2022* | *2024* | *2026* | *2028* | *2030* | *2032* | *2034* | *2036* |

***Option 1:*** *Health State A in 2 years’ time (2018)*

***Option 2:*** *Health State B in 20 years’ time (2036)*

***[If Option 1, go to part (j)***

*(c) Which of the following options would you prefer?*

*Option 1: Health State A in 2 years’ time (2018)*

| ***Full Health*** | ***Health State A*** | ***Full Health*** | ***Full Health*** | ***Full Health*** | ***Full Health*** | ***Full Health*** | ***Full Health*** | ***Full Health*** | ***Full Health*** | ***Full Health*** |
| --- | --- | --- | --- | --- | --- | --- | --- | --- | --- | --- |
| *2016* | *2018* | *2020* | *2022* | *2024* | *2026* | *2028* | *2030* | *2032* | *2034* | *2036* |

*or*

*Option 2: Health State B in 16 years’ time (2032)*

| ***Full Health*** | ***Full Health*** | ***Full Health*** | ***Full Health*** | ***Full Health*** | ***Full Health*** | ***Full Health*** | ***Full Health*** | ***Health State B*** | ***Full Health*** | ***Full Health*** |
| --- | --- | --- | --- | --- | --- | --- | --- | --- | --- | --- |
| *2016* | *2018* | *2020* | *2022* | *2024* | *2026* | *2028* | *2030* | *2032* | *2034* | *2036* |

***Option 1:*** *Health State A in 2 years’ time (2018)*

***Option 2:*** *Health State B in 16 years’ time (2032)*

***[If Option 2, go to part (e)]***

*(d) Which of the following options would you prefer?*

*Option 1: Health State A in 2 years’ time (2018)*

| ***Full Health*** | ***Health State A*** | ***Full Health*** | ***Full Health*** | ***Full Health*** | ***Full Health*** | ***Full Health*** | ***Full Health*** | ***Full Health*** | ***Full Health*** | ***Full Health*** |
| --- | --- | --- | --- | --- | --- | --- | --- | --- | --- | --- |
| *2016* | *2018* | *2020* | *2022* | *2024* | *2026* | *2028* | *2030* | *2032* | *2034* | *2036* |

*or*

*Option 2: Health State B in 18 years’ time (2034)*

| ***Full Health*** | ***Full Health*** | ***Full Health*** | ***Full Health*** | ***Full Health*** | ***Full Health*** | ***Full Health*** | ***Full Health*** | ***Full Health*** | ***Health State B*** | ***Full Health*** |
| --- | --- | --- | --- | --- | --- | --- | --- | --- | --- | --- |
| *2016* | *2018* | *2020* | *2022* | *2024* | *2026* | *2028* | *2030* | *2032* | *2034* | *2036* |

***Option 1:*** *Health State A in 2 years’ time (2018)*

***Option 2:*** *Health State B in 18 years’ time (2034)*

***[Now go to question 6]***

*(e) Which of the following options would you prefer?*

*Option 1: Health State A in 2 years’ time (2018)*

| ***Full Health*** | ***Health State A*** | ***Full Health*** | ***Full Health*** | ***Full Health*** | ***Full Health*** | ***Full Health*** | ***Full Health*** | ***Full Health*** | ***Full Health*** | ***Full Health*** |
| --- | --- | --- | --- | --- | --- | --- | --- | --- | --- | --- |
| *2016* | *2018* | *2020* | *2022* | *2024* | *2026* | *2028* | *2030* | *2032* | *2034* | *2036* |

*or*

*Option 2: Health State B in 14 years’ time (2030)*

| ***Full Health*** | ***Full Health*** | ***Full Health*** | ***Full Health*** | ***Full Health*** | ***Full Health*** | ***Full Health*** | ***Health State B*** | ***Full Health*** | ***Full Health*** | ***Full Health*** |
| --- | --- | --- | --- | --- | --- | --- | --- | --- | --- | --- |
| *2016* | *2018* | *2020* | *2022* | *2024* | *2026* | *2028* | *2030* | *2032* | *2034* | *2036* |

***Option 1:*** *Health State A in 2 years’ time (2018)*

***Option 2:*** *Health State B in 14 years’ time (2030)*

***[Now go to question 6]***

*(f) Which of the following options would you prefer?*

*Option 1: Health State A in 2 years’ time (2018)*

| ***Full Health*** | ***Health State A*** | ***Full Health*** | ***Full Health*** | ***Full Health*** | ***Full Health*** | ***Full Health*** | ***Full Health*** | ***Full Health*** | ***Full Health*** | ***Full Health*** |
| --- | --- | --- | --- | --- | --- | --- | --- | --- | --- | --- |
| *2016* | *2018* | *2020* | *2022* | *2024* | *2026* | *2028* | *2030* | *2032* | *2034* | *2036* |

*or*

*Option 2: Health State B in 4 years’ time (2020)*

| ***Full Health*** | ***Full Health*** | ***Health State B*** | ***Full Health*** | ***Full Health*** | ***Full Health*** | ***Full Health*** | ***Full Health*** | ***Full Health*** | ***Full Health*** | ***Full Health*** |
| --- | --- | --- | --- | --- | --- | --- | --- | --- | --- | --- |
| *2016* | *2018* | *2020* | *2022* | *2024* | *2026* | *2028* | *2030* | *2032* | *2034* | *2036* |

***Option 1:*** *Health State A in 2 years’ time (2018)*

***Option 2:*** *Health State B in 4 years’ time (2020)*

***[If Option 2, go to question 6]***

*(g) Which of the following options would you prefer?*

*Option 1: Health State A in 2 years’ time (2018)*

| ***Full Health*** | ***Health State A*** | ***Full Health*** | ***Full Health*** | ***Full Health*** | ***Full Health*** | ***Full Health*** | ***Full Health*** | ***Full Health*** | ***Full Health*** | ***Full Health*** |
| --- | --- | --- | --- | --- | --- | --- | --- | --- | --- | --- |
| *2016* | *2018* | *2020* | *2022* | *2024* | *2026* | *2028* | *2030* | *2032* | *2034* | *2036* |

*or*

*Option 2: Health State B in 8 years’ time (2024)*

| ***Full Health*** | ***Full Health*** | ***Full Health*** | ***Full Health*** | ***Health State B*** | ***Full Health*** | ***Full Health*** | ***Full Health*** | ***Full Health*** | ***Full Health*** | ***Full Health*** |
| --- | --- | --- | --- | --- | --- | --- | --- | --- | --- | --- |
| *2016* | *2018* | *2020* | *2022* | *2024* | *2026* | *2028* | *2030* | *2032* | *2034* | *2036* |

***Option 1:*** *Health State A in 2 years’ time (2018)*

***Option 2:*** *Health State B in 8 years’ time (2024)*

***[If Option 1, go to part (i)]***

*(h) Which of the following options would you prefer?*

*Option 1: Health State A in 2 years’ time (2018)*

| ***Full Health*** | ***Health State A*** | ***Full Health*** | ***Full Health*** | ***Full Health*** | ***Full Health*** | ***Full Health*** | ***Full Health*** | ***Full Health*** | ***Full Health*** | ***Full Health*** |
| --- | --- | --- | --- | --- | --- | --- | --- | --- | --- | --- |
| *2016* | *2018* | *2020* | *2022* | *2024* | *2026* | *2028* | *2030* | *2032* | *2034* | *2036* |

*or*

*Option 2: Health State B in 6 years’ time (2022)*

| ***Full Health*** | ***Full Health*** | ***Full Health*** | ***Health State B*** | ***Full Health*** | ***Full Health*** | ***Full Health*** | ***Full Health*** | ***Full Health*** | ***Full Health*** | ***Full Health*** |
| --- | --- | --- | --- | --- | --- | --- | --- | --- | --- | --- |
| *2016* | *2018* | *2020* | *2022* | *2024* | *2026* | *2028* | *2030* | *2032* | *2034* | *2036* |

***Option 1:*** *Health State A in 2 years’ time (2018)*

***Option 2:*** *Health State B in 6 years’ time (2022)*

***[Now go to question 6]***

*(i) Which of the following options would you prefer?*

*Option 1: Health State A in 2 years’ time (2018)*

| ***Full Health*** | ***Health State A*** | ***Full Health*** | ***Full Health*** | ***Full Health*** | ***Full Health*** | ***Full Health*** | ***Full Health*** | ***Full Health*** | ***Full Health*** | ***Full Health*** |
| --- | --- | --- | --- | --- | --- | --- | --- | --- | --- | --- |
| *2016* | *2018* | *2020* | *2022* | *2024* | *2026* | *2028* | *2030* | *2032* | *2034* | *2036* |

*or*

*Option 2: Health State B in 10 years’ time (2026)*

| ***Full Health*** | ***Full Health*** | ***Full Health*** | ***Full Health*** | ***Full Health*** | ***Health State B*** | ***Full Health*** | ***Full Health*** | ***Full Health*** | ***Full Health*** | ***Full Health*** |
| --- | --- | --- | --- | --- | --- | --- | --- | --- | --- | --- |
| *2016* | *2018* | *2020* | *2022* | *2024* | *2026* | *2028* | *2030* | *2032* | *2034* | *2036* |

***Option 1:*** *Health State A in 2 years’ time (2018)*

***Option 2:*** *Health State B in 10 years’ time (2026)*

***[Now go to question 6]***

*(j) Is there any far off time in the future at which being in Health State B would be preferable to being in Health State A two years from now (ie. in 2018)?*

*Please indicate your answer by writing a number in the box below. (If you would never prefer Health State B at any point in the future, to Health State A two years from now, please write the letter “N” in the box below, instead of a number).*

*“Rather than Health State A two years’ from now, I would prefer Health State B, providing it*

*were at least years from now.”*

*Randomised into two versions:*

*6: VERSION 1: This question is about views on risks to health. You have two options, 1 and 2. The options vary in terms of your health state over the next two weeks.*

*In option 1, there is a chance of being in Full Health, but also a chance of being in Health State B. So option 1 is like a lottery: you might be in Full Health, but you may also be in Health State B. You do not know beforehand which of these health states you will experience: Full Health or Health State B.*

*In option 2, you will definitely be in Health State A.*

*In the following questions, we will vary the chance of Full Health in Option 1 and, in each case, ask you to say whether you prefer Option 1 or Option 2 by marking an ‘X’ in the appropriate box.*

*(a) Which option do you prefer:*

| ***Option 1***  *5 out of 10 chance of Full Health* 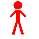  5 out of 10 chance of Health State B 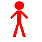   \| 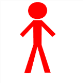 \| 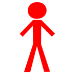 \| 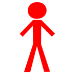 \| 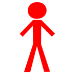 \| 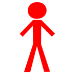 \| \| --- \| --- \| --- \| --- \| --- \| \| 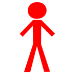 \| 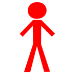 \| 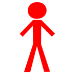 \| 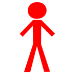 \| 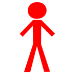 \| | ***Option 2***  *Definitely Health State A* 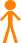   \| 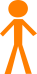 \| 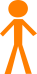 \| 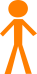 \| 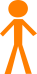 \| 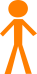 \| \| --- \| --- \| --- \| --- \| --- \| \| 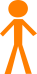 \| 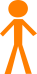 \| 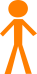 \| 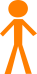 \| 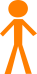 \| |
| --- | --- | --- | --- | --- | --- | --- | --- | --- | --- | --- | --- | --- | --- | --- | --- | --- | --- | --- | --- | --- | --- |

***Option 1: 5 out of 10*** *chance of Full Health, and* ***5 out of 10*** *chance of Health State B*

*or*

***Option 2:*** *Definitely Health State A*

***[If Option 2, go to part (f)]***

*(b) Which option do you prefer:*

| ***Option 1***  *1 out of 10 chance of Full Health* 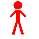  9 out of 10 chance of Health State B 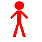   \| 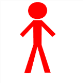 \| 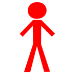 \| 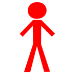 \| 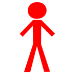 \| 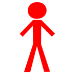 \| \| --- \| --- \| --- \| --- \| --- \| \| 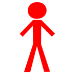 \| 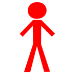 \| 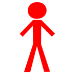 \| 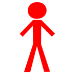 \| 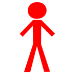 \| | ***Option 2***  *Definitely Health State A* 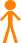   \| 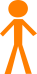 \| 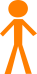 \| 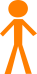 \| 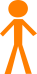 \| 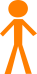 \| \| --- \| --- \| --- \| --- \| --- \| \| 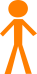 \| 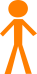 \| 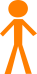 \| 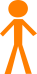 \| 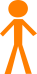 \| |
| --- | --- | --- | --- | --- | --- | --- | --- | --- | --- | --- | --- | --- | --- | --- | --- | --- | --- | --- | --- | --- | --- |

***Option 1: 1 out of 10*** *chance of Full Health, and* ***9 out of 10*** *chance of Health State B*

*or*

***Option 2:*** *Definitely Health State A*

***[If Option 1, go to question 6.5]***

*(c) Which option do you prefer:*

| ***Option 1***  *3 out of 10 chance of Full Health* 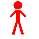  7 out of 10 chance of Health State B 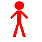   \| 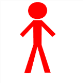 \| 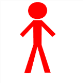 \| 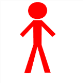 \| 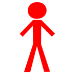 \| 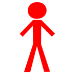 \| \| --- \| --- \| --- \| --- \| --- \| \| 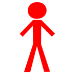 \| 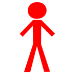 \| 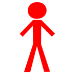 \| 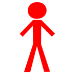 \| 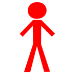 \| | ***Option 2***  *Definitely Health State A* 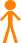   \| 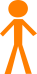 \| 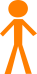 \| 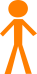 \| 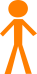 \| 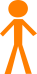 \| \| --- \| --- \| --- \| --- \| --- \| \| 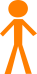 \| 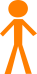 \| 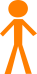 \| 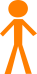 \| 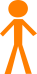 \| |
| --- | --- | --- | --- | --- | --- | --- | --- | --- | --- | --- | --- | --- | --- | --- | --- | --- | --- | --- | --- | --- | --- |

***Option 1: 3 out of 10*** *chance of Full Health, and* ***7 out of 10*** *chance of Health State B*

*or*

***Option 2:*** *Definitely Health State A*

***[If Option 2, go to part (e)]***

*(d) Which option do you prefer:*

| ***Option 1***  *2 out of 10 chance of Full Health* 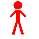  8 out of 10 chance of Health State B 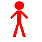   \| 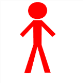 \| 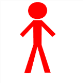 \| 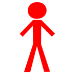 \| 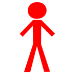 \| 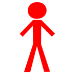 \| \| --- \| --- \| --- \| --- \| --- \| \| 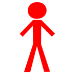 \| 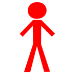 \| 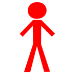 \| 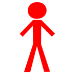 \| 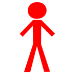 \| | ***Option 2***  *Definitely Health State A* 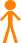   \| 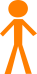 \| 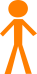 \| 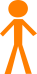 \| 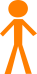 \| 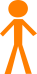 \| \| --- \| --- \| --- \| --- \| --- \| \| 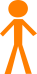 \| 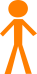 \| 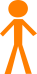 \| 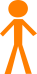 \|  \| |
| --- | --- | --- | --- | --- | --- | --- | --- | --- | --- | --- | --- | --- | --- | --- | --- | --- | --- | --- | --- | --- | --- |

***Option 1: 2 out of 10*** *chance of Full Health, and* ***8 out of 10*** *chance of Health State B*

*or*

***Option 2:*** *Definitely Health State A*

***[Now go to question 6.5]***

*(e) Which option do you prefer:*

| ***Option 1***  *4 out of 10 chance of Full Health*  6 out of 10 chance of Health State B   \|  \|  \|  \|  \|  \| \| --- \| --- \| --- \| --- \| --- \| \|  \|  \|  \|  \|  \| | ***Option 2***  *Definitely Health State A*   \|  \|  \|  \|  \|  \| \| --- \| --- \| --- \| --- \| --- \| \|  \|  \|  \|  \|  \| |
| --- | --- | --- | --- | --- | --- | --- | --- | --- | --- | --- | --- | --- | --- | --- | --- | --- | --- | --- | --- | --- | --- |

***Option 1: 4 out of 10*** *chance of Full Health, and* ***6 out of 10*** *chance of Health State B*

*or*

***Option 2:*** *Definitely Health State A*

***[Now go to question 6.5]***

*(f) Which option do you prefer:*

| ***Option 1***  *9 out of 10 chance of Full Health*  1 out of 10 chance of Health State B   \|  \|  \|  \|  \|  \| \| --- \| --- \| --- \| --- \| --- \| \|  \|  \|  \|  \|  \| | ***Option 2***  *Definitely Health State A*   \|  \|  \|  \|  \|  \| \| --- \| --- \| --- \| --- \| --- \| \|  \|  \|  \|  \|  \| |
| --- | --- | --- | --- | --- | --- | --- | --- | --- | --- | --- | --- | --- | --- | --- | --- | --- | --- | --- | --- | --- | --- |

***Option 1: 9 out of 10*** *chance of Full Health, and* ***1 out of 10*** *chance of Health State B*

*or*

***Option 2:*** *Definitely Health State A*

***[If Option 2, go to part (j)]***

*(g) Which option do you prefer:*

| ***Option 1***  *7 out of 10 chance of Full Health*  3 out of 10 chance of Health State B   \|  \|  \|  \|  \|  \| \| --- \| --- \| --- \| --- \| --- \| \|  \|  \|  \|  \|  \| | ***Option 2***  *Definitely Health State A*   \|  \|  \|  \|  \|  \| \| --- \| --- \| --- \| --- \| --- \| \|  \|  \|  \|  \|  \| |
| --- | --- | --- | --- | --- | --- | --- | --- | --- | --- | --- | --- | --- | --- | --- | --- | --- | --- | --- | --- | --- | --- |

***Option 1: 7 out of 10*** *chance of Full Health, and* ***3 out of 10*** *chance of Health State B*

*or*

***Option 2:*** *Definitely Health State A*

***[If Option 2, go to part (i)]***

*(h) Which option do you prefer:*

| ***Option 1***  *6 out of 10 chance of Full Health*  4 out of 10 chance of Health State B   \|  \|  \|  \|  \|  \| \| --- \| --- \| --- \| --- \| --- \| \|  \|  \|  \|  \|  \| | ***Option 2***  *Definitely Health State A*   \|  \|  \|  \|  \|  \| \| --- \| --- \| --- \| --- \| --- \| \|  \|  \|  \|  \|  \| |
| --- | --- | --- | --- | --- | --- | --- | --- | --- | --- | --- | --- | --- | --- | --- | --- | --- | --- | --- | --- | --- | --- |

***Option 1: 6 out of 10*** *chance of Full Health, and* ***4 out of 10*** *chance of Health State B*

XX

*or*

***Option 2:*** *Definitely Health State A*

***[Now go to question 6.5]***

*(i) Which option do you prefer:*

| ***Option 1***  *8 out of 10 chance of Full Health*  2 out of 10 chance of Health State B   \|  \|  \|  \|  \|  \| \| --- \| --- \| --- \| --- \| --- \| \|  \|  \|  \|  \|  \| | ***Option 2***  *Definitely Health State A*   \|  \|  \|  \|  \|  \| \| --- \| --- \| --- \| --- \| --- \| \|  \|  \|  \|  \|  \| |
| --- | --- | --- | --- | --- | --- | --- | --- | --- | --- | --- | --- | --- | --- | --- | --- | --- | --- | --- | --- | --- | --- |

***Option 1: 8 out of 10*** *chance of Full Health, and* ***2 out of 10*** *chance of Health State B*

*or*

***Option 2:*** *Definitely Health State A*

***[Now go to question 6.5]***

*(j) You have indicated that you would not prefer Option 1 to Option 2 even if the chance of Health State B in Option 1 was as low as 1 out of 10. How low would the chance of Health State B in Option 1 (probably Full Health but a small chance of Health State B) need to be for you to prefer Option 1 to Option 2 (Definitely Health State A)?*

| ***Option 1***  *Probably Full Health*  Small chance of Health State B | ***Option 2***  *Definitely Health State A*   \|  \|  \|  \|  \|  \| \| --- \| --- \| --- \| --- \| --- \| \|  \|  \|  \|  \|  \| |
| --- | --- | --- | --- | --- | --- | --- | --- | --- | --- | --- | --- |

*Please indicate your answer by writing a number bigger than 10 in the box below; the number can be as big as you want, with as many digits as you need. (If you would never prefer Option 1, no matter how unlikely Health State B is, please write the letter “N” in the box instead of a number).*

*I would prefer Option 1 to Option 2, if the chance of Health State B in Option 1 was less than*

*1 out of*

*6: VERSION 2: This question is about views on risks to health. You have two options, 1 and 2. The options vary in terms of your health state over the next two weeks.*

*In option 1, you will definitely be in Health State A.*

*In option 2, there is a chance of being in Full Health, but also a chance of being in Health State B. So option 2 is like a lottery: you might be in Full Health, but you may also be in Health State B. You do not know beforehand which of these health states you will experience: Full Health or Health State B.*

*In the following questions, we will vary the chance of Full Health in Option 2 and, in each case, ask you to say whether you prefer Option 1 or Option 2 by marking an ‘X’ in the appropriate box.*

*(a) Which option do you prefer:*

| ***Option 1***  *Definitely Health State A*   \|  \|  \|  \|  \|  \| \| --- \| --- \| --- \| --- \| --- \| \|  \|  \|  \|  \|  \| | ***Option 2***  *5 out of 10 chance of Full Health*  5 out of 10 chance of Health State B   \|  \|  \|  \|  \|  \| \| --- \| --- \| --- \| --- \| --- \| \|  \|  \|  \|  \|  \| |
| --- | --- | --- | --- | --- | --- | --- | --- | --- | --- | --- | --- | --- | --- | --- | --- | --- | --- | --- | --- | --- | --- |

***Option 1:*** *Definitely Health State A*

*or*

***Option 2:*** ***5 out of 10*** *chance of Full Health, and* ***5 out of 10*** *chance of Health State B*

***[If Option 1, go to part (f)]***

*(b) Which option do you prefer:*

| ***Option 1***  *Definitely Health State A*   \|  \|  \|  \|  \|  \| \| --- \| --- \| --- \| --- \| --- \| \|  \|  \|  \|  \|  \| | ***Option 2***  *1 out of 10 chance of Full Health*  9 out of 10 chance of Health State B   \|  \|  \|  \|  \|  \| \| --- \| --- \| --- \| --- \| --- \| \|  \|  \|  \|  \|  \| |
| --- | --- | --- | --- | --- | --- | --- | --- | --- | --- | --- | --- | --- | --- | --- | --- | --- | --- | --- | --- | --- | --- |

***Option 1:*** *Definitely Health State A*

*or*

***Option 2:*** ***1 out of 10*** *chance of Full Health, and* ***9 out of 10*** *chance of Health State B*

***[If Option 2, go to question 6.5]***

*(c) Which option do you prefer:*

| ***Option 1***  *Definitely Health State A*   \|  \|  \|  \|  \|  \| \| --- \| --- \| --- \| --- \| --- \| \|  \|  \|  \|  \|  \| | ***Option 2***  *3 out of 10 chance of Full Health*  7 out of 10 chance of Health State B   \|  \|  \|  \|  \|  \| \| --- \| --- \| --- \| --- \| --- \| \|  \|  \|  \|  \|  \| |
| --- | --- | --- | --- | --- | --- | --- | --- | --- | --- | --- | --- | --- | --- | --- | --- | --- | --- | --- | --- | --- | --- |

***Option 1:*** *Definitely Health State A*

*or*

***Option 2:*** ***3 out of 10*** *chance of Full Health, and* ***7 out of 10*** *chance of Health State B*

***[If Option 1, go to part (e)]***

*(d) Which option do you prefer:*

| ***Option 1***  *Definitely Health State A*   \|  \|  \|  \|  \|  \| \| --- \| --- \| --- \| --- \| --- \| \|  \|  \|  \|  \|  \| | ***Option 2***  *2 out of 10 chance of Full Health*  8 out of 10 chance of Health State B   \|  \|  \|  \|  \|  \| \| --- \| --- \| --- \| --- \| --- \| \|  \|  \|  \|  \|  \| |
| --- | --- | --- | --- | --- | --- | --- | --- | --- | --- | --- | --- | --- | --- | --- | --- | --- | --- | --- | --- | --- | --- |

***Option 1:*** *Definitely Health State A*

*or*

***Option 2:*** ***2 out of 10*** *chance of Full Health, and* ***8 out of 10*** *chance of Health State B*

***[Now go to question 6.5]***

*(e) Which option do you prefer:*

| ***Option 1***  *Definitely Health State A*   \|  \|  \|  \|  \|  \| \| --- \| --- \| --- \| --- \| --- \| \|  \|  \|  \|  \|  \| | ***Option 2***  *4 out of 10 chance of Full Health*  6 out of 10 chance of Health State B   \|  \|  \|  \|  \|  \| \| --- \| --- \| --- \| --- \| --- \| \|  \|  \|  \|  \|  \| |
| --- | --- | --- | --- | --- | --- | --- | --- | --- | --- | --- | --- | --- | --- | --- | --- | --- | --- | --- | --- | --- | --- |

***Option 1:*** *Definitely Health State A*

*or*

***Option 2:*** ***4 out of 10*** *chance of Full Health, and* ***6 out of 10*** *chance of Health State B*

***[Now go to question 6.5]***

*(f) Which option do you prefer:*

| ***Option 2***  *Definitely Health State A*   \|  \|  \|  \|  \|  \| \| --- \| --- \| --- \| --- \| --- \| \|  \|  \|  \|  \|  \| | ***Option 1***  *9 out of 10 chance of Full Health*  1 out of 10 chance of Health State B   \|  \|  \|  \|  \|  \| \| --- \| --- \| --- \| --- \| --- \| \|  \|  \|  \|  \|  \| |
| --- | --- | --- | --- | --- | --- | --- | --- | --- | --- | --- | --- | --- | --- | --- | --- | --- | --- | --- | --- | --- | --- |

***Option 1:*** *Definitely Health State A*

*or*

***Option 2:*** ***9 out of 10*** *chance of Full Health, and* ***1 out of 10*** *chance of Health State B*

***[If Option 1, go to part (j)]***

*(g) Which option do you prefer:*

| ***Option 1***  *Definitely Health State A*   \|  \|  \|  \|  \|  \| \| --- \| --- \| --- \| --- \| --- \| \|  \|  \|  \|  \|  \| | ***Option 2***  *7 out of 10 chance of Full Health*  3 out of 10 chance of Health State B   \|  \|  \|  \|  \|  \| \| --- \| --- \| --- \| --- \| --- \| \|  \|  \|  \|  \|  \| |
| --- | --- | --- | --- | --- | --- | --- | --- | --- | --- | --- | --- | --- | --- | --- | --- | --- | --- | --- | --- | --- | --- |

***Option 1:*** *Definitely Health State A*

*or*

***Option 2:*** ***7 out of 10*** *chance of Full Health, and* ***3 out of 10*** *chance of Health State B*

***[If Option 1, go to part (i)]***

*(h) Which option do you prefer:*

| ***Option 1***  *Definitely Health State A*   \|  \|  \|  \|  \|  \| \| --- \| --- \| --- \| --- \| --- \| \|  \|  \|  \|  \|  \| | ***Option 2***  *6 out of 10 chance of Full Health*  4 out of 10 chance of Health State B   \|  \|  \|  \|  \|  \| \| --- \| --- \| --- \| --- \| --- \| \|  \|  \|  \|  \|  \| |
| --- | --- | --- | --- | --- | --- | --- | --- | --- | --- | --- | --- | --- | --- | --- | --- | --- | --- | --- | --- | --- | --- |

***Option 1:*** *Definitely Health State A*

XX

*or*

***Option 2:*** ***6 out of 10*** *chance of Full Health, and* ***4 out of 10*** *chance of Health State B*

***[Now go to question 6.5]***

*(i) Which option do you prefer:*

| ***Option 1***  *Definitely Health State A*   \|  \|  \|  \|  \|  \| \| --- \| --- \| --- \| --- \| --- \| \|  \|  \|  \|  \|  \| | ***Option 2***  *8 out of 10 chance of Full Health*  2 out of 10 chance of Health State B   \|  \|  \|  \|  \|  \| \| --- \| --- \| --- \| --- \| --- \| \|  \|  \|  \|  \|  \| |
| --- | --- | --- | --- | --- | --- | --- | --- | --- | --- | --- | --- | --- | --- | --- | --- | --- | --- | --- | --- | --- | --- |

***Option 1:*** *Definitely Health State A*

*or*

***Option 2:*** ***8 out of 10*** *chance of Full Health, and* ***2 out of 10*** *chance of Health State B*

***[Now go to question 6.5]***

*(j) You have indicated that you would not prefer Option 2 to Option 1 even if the chance of Health State B in Option 2 was as low as 1 out of 10. How low would the chance of Health State B in Option 2 (probably Full Health but a small chance of Health State B) need to be for you to prefer Option 2 to Option 1 (Definitely Health State A)?*

| ***Option 1***  *Definitely Health State A*   \|  \|  \|  \|  \|  \| \| --- \| --- \| --- \| --- \| --- \| \|  \|  \|  \|  \|  \| | ***Option 2***  *Probably Full Health*  Small chance of Health State B |
| --- | --- | --- | --- | --- | --- | --- | --- | --- | --- | --- | --- |

*Please indicate your answer by writing a number bigger than 10 in the box below; the number can be as big as you want, with as many digits as you need. (If you would never prefer Option 2, no matter how unlikely Health State B is, please write the letter “N” in the box instead of a number).*

*I would prefer Option 2 to Option 1, if the chance of Health State B in Option 2 was less than*

*1 out of*

*Q6.5 VERSION 1: Do you prefer bananas or oranges?*

*(a) Which option do you prefer:*

| ***Option 1: Bananas*** | ***Option 2: Oranges*** |
| --- | --- |

***Option 1: Bananas***

***Option 2:*** ***Oranges***

***Randomised into 2 versions. This question should exactly follow the randomisation at Q6***

*Q 6.5 VERSION 2: (a) Which option do you prefer:*

| ***Option 1: Oranges*** | ***Option 2: Bananas*** |
| --- | --- |

***Option 1: Oranges***

***Option 2: Bananas***

*7. Now imagine that you have been in* ***Health State A*** *for the last five days: You have*

- *a temperature,*
- *aching muscles,*
- *a headache,*
- *a dry chesty cough,*
- *a sore throat,*
- *and you feel weak.*

*(a) At this point, do you think you would go to see a GP about these symptoms?*

*Definitely*

*Probably*

*Probably not*

*Definitely not*

*Don’t know*

***[If ‘definitely not,’ go to part (c)]***

*(b) If you went to see a GP about these symptoms, do you think you would ask for antibiotics?*

*Definitely*

*Probably*

*Probably not*

*Definitely not*

*Don’t know*

*(c) Do you think antibiotics would be likely to help these symptoms?*

*Definitely*

*Probably*

*Probably not*

*Definitely not*

*Don’t know*

*(d) To the best of your knowledge, have you taken antibiotics for symptoms similar to these in the last 12 months?*

*Yes No*

*(e) To the best of your knowledge, have you taken antibiotics for any other health problem in the last 12 months?*

*Yes No*

***[If respondent answers ‘No’ in both parts (d) and (e), go to part (g)].***

*(f) To the best of your knowledge, during the last 12 months, on how many separate occasions have you been unwell and taken a course of antibiotics? Please indicate your answer by entering a number in the box below:*

*(g) If you were prescribed antibiotics for a health problem (not necessarily one described in this questionnaire) do you think you would take the full course?*

*Definitely*

*Probably*

*Probably not*

*Definitely not*

*Don’t know*

***[If respondent answered “no” to the first part of question 7 in Section 1 (i.e. if they have no dependent children), go to question 11]***

*8. This question is about the health of your child. (If you have more than one child, think of your youngest child).*

*Imagine that your child has been in* ***Health State A*** *for the last five days: He/she has*

- *a temperature,*
- *aching muscles,*
- *a headache,*
- *a dry chesty cough,*
- *a sore throat,*
- *and feels weak.*

*(a) At this point, do you think you would take your child to see a GP about these symptoms?*

*Definitely*

*Probably*

*Probably not*

*Definitely not*

*Don’t know*

***[If ‘definitely not,’ go to part (c)]***

*(b) If you did take your child to see a GP about these symptoms, do you think you would ask for antibiotics?*

*Definitely*

*Probably*

*Probably not*

*Definitely not*

*Don’t know*

*(c) Do you think antibiotics would be likely to help your child in this situation?*

*Definitely*

*Probably*

*Probably not*

*Definitely not*

*Don’t know*

*(d) To the best of your knowledge, has your child taken antibiotics for symptoms similar to thesein the last 12 months?*

*Yes No*

*(e) To the best of your knowledge, has your child taken antibiotics for any other health problem in the last 12 months?*

*Yes No*

***[If respondent answers ‘No’ in both parts (d) and (e), go to part (g)].***

*(f) To the best of your knowledge, during the last 12 months, on how many separate occasions has your child been unwell and taken a course of antibiotics? Please indicate your answer by entering a number in the box below:*

*(g) If your child were prescribed antibiotics for a health problem (not necessarily one described in this questionnaire) do you think you would make them take the full course?*

*Definitely*

*Probably*

*Probably not*

*Definitely not*

*Don’t know*

The next question contains information about antibiotic resistance, a topic which you may have seen in the news recently.

***The information at the start of Question 11 is randomised into 3 versions, targeting respective responses of N= 1000; N=1500; N=1500***

The next question contains information about antibiotic resistance, a topic which you may have seen in the news recently.

11 Version 1: Antibiotic resistance happens when an antibiotic no longer kills or controls growing bacteria. It is an increasingly serious threat to public health. Without antibiotics that work well, many routine treatments will become increasingly dangerous. Setting broken bones, and even basic operations, rely on access to antibiotics that work. Antibiotic resistance is believed to be caused by unnecessary use of antibiotics, and inappropriate use, such as not taking them as prescribed, skipping doses, or saving them for later use.

Version 2: Most people get cold or flu symptoms every year, and these usually get better on their own. Temperatures sometimes last for days, while coughs can last for weeks, and antibiotics generally don’t help. Antibiotics should not be taken for cold and flu symptoms. Taking antibiotics when they are not needed encourages bacteria to become resistant. This means antibiotics may not work for future serious illnesses that can only be cured by antibiotics. Most cold and flu symptoms are best treated at home by taking paracetamol or ibuprofen, and getting plenty of fluids and sleep.

Version 3: Most people get cold or flu symptoms every year, and these usually get better on their own. Temperatures sometimes last for days, while coughs can last for weeks, and antibiotics generally don’t help. Antibiotics should not be taken for cold and flu symptoms. Taking antibiotics encourages bacteria to become resistant. Some killer diseases are already resistant to several antibiotics. Antibiotic resistance is an increasingly serious threat to everyone’s health. Soon we will not be able to find antibiotics that can cure serious illnesses. Even worse, without antibiotics that work, even minor injuries and routine operations will become increasingly dangerous. You can also pass on resistant bugs to people you care about. Most cold and flu symptoms are best treated at home by taking paracetamol or ibuprofen, and getting plenty of fluids and sleep.

(a) To what extent is this information new to you?

*Very new*

*Somewhat new*

*Not very new*

*Not at all new*

*(b) How will this information affect whether you visit a doctor the next time you have symptoms like Health State A? (****Health State A****: You have*

- *a temperature,*
- *aching muscles,*
- *a headache,*
- *a dry chesty cough,*
- *a sore throat,*
- *and you feel weak ).*

*Much more likely to visit doctor*

*More likely to visit doctor*

*The information would not affect whether I visit doctor*

*Less likely to visit doctor*

*Much less likely to visit doctor*

*Don’t know*

*(c) How will this information affect the likelihood of you asking a doctor for antibiotics if you were to visit for these symptoms?*

*Much more likely to ask for antibiotics*

*More likely to ask for antibiotics*

*The information would not affect whether I ask for antibiotics*

*Less likely to ask for antibiotics*

*Much less likely to ask for antibiotics*

*Don’t know*

***[If respondent answered no to first part of question 7 in Section 1 (i.e. if they have no dependent children), go now to part (f).]***

*Parts (d) and (e) are about the health of your child. If you have more than one child, think of your youngest child.*

*(d) How will this information affect whether you take your child to visit a doctor the next time he/she has symptoms like Health State A? (****Health State A****: He/she has*

- *a temperature,*
- *aching muscles,*
- *a headache,*
- *a dry chesty cough,*
- *a sore throat,*
- *and feels weak).*

*Much more likely to take child to visit doctor*

*More likely to take child to visit doctor*

*The information would not affect whether I take child to visit doctor*

*Less likely to take child to visit doctor*

*Much less likely to take child to visit doctor*

*Don’t know*

*(e) How will this information affect the likelihood of you asking a doctor for antibiotics for your child if you were to visit for these symptoms?*

*Much more likely to ask for antibiotics*

*More likely to ask for antibiotics*

*The information would not affect whether I ask for antibiotics*

*Less likely to ask for antibiotics*

*Much less likely to ask for antibiotics*

*Don’t know*

*(f) To what extent does this information worry you?*

*Very much*

*Somewhat*

*Not very*

*Not at all*

*(g) To what extent is this information relevant to you?*

*Very relevant*

*Somewhat relevant*

*Not very relevant*

*Not at all relevant*

*(h) By not taking antibiotics for cold and flu symptoms, how much can you personally help reduce the threat of antibiotic resistance?*

*Very much*

*Somewhat*

*Not very much*

*Not at all*

*(i) If most people did not take antibiotics for cold and flu symptoms, how much would this help reduce the threat of antibiotic resistance?*

*Very much*

*Somewhat*

*Not very much*

*Not at all*

*(j) How confident are you that you can manage future* cold and flu symptoms at home, without taking any antibiotics?

*Very confident*

*Somewhat confident*

*Not very confident*

*Not at all confident*

Section 3

Some scientific studies have shown that certain attitudes towards health and healthy behaviour, are related to personality. To help us to learn more about this, we would like to finish the questionnaire by asking you some questions about how you see yourself.

How well do the following statements describe your personality?

1. (a) “I see myself as someone who is reserved.”

*Agree strongly*

*Agree a little*

*Neither agree nor disagree*

*Disagree a little*

*Disagree strongly*

(b) “I see myself as someone who is generally trusting.”

*Agree strongly*

*Agree a little*

*Neither agree nor disagree*

*Disagree a little*

*Disagree strongly*

(c) “I see myself as someone who tends to be lazy.”

*Agree strongly*

*Agree a little*

*Neither agree nor disagree*

*Disagree a little*

*Disagree strongly*

(d) “I see myself as someone who is relaxed, handles stress well.”

*Agree strongly*

*Agree a little*

*Neither agree nor disagree*

*Disagree a little*

*Disagree strongly*

(e) “I see myself as someone who has few artistic interests.”

*Agree strongly*

*Agree a little*

*Neither agree nor disagree*

*Disagree a little*

*Disagree strongly*

(f) “I see myself as someone who is outgoing, sociable.”

*Agree strongly*

*Agree a little*

*Neither agree nor disagree*

*Disagree a little*

*Disagree strongly*

(g) “I see myself as someone who tends to find fault with others.”

*Agree strongly*

*Agree a little*

*Neither agree nor disagree*

*Disagree a little*

*Disagree strongly*

(h) “I see myself as someone who does a thorough job.”

*Agree strongly*

*Agree a little*

*Neither agree nor disagree*

*Disagree a little*

*Disagree strongly*

(i) “I see myself as someone who gets nervous easily.”

*Agree strongly*

*Agree a little*

*Neither agree nor disagree*

*Disagree a little*

*Disagree strongly*

(j) “I see myself as someone who has an active imagination.”

*Agree strongly*

*Agree a little*

*Neither agree nor disagree*

*Disagree a little*

*Disagree strongly*

2. (a) How do you see yourself? Are you generally a person who is fully willing to take risks or do you try to avoid taking risks? *Please choose a box on the scale below, where 0 means “risk averse” and 10 means “fully prepared to take risks”:*

Not at all prepared to take risks

Fully prepared to take risks

(b) People can behave differently in different situations. How would you rate your willingness to take risks with your health? *Please choose a box on the scale below, where 0 means “risk averse” and 10 means “fully prepared to take risks”:*

Not at all prepared to take risks

Fully prepared to take risks

Finally, if you have any other comments, please enter them here.

**You have now reached the end of the questionnaire. Thank you very much for taking the time to complete it. The information you have provided will be extremely useful for our research. We would like to remind you that all the information you have given will be anonymised and stored securely.**

# Appendix 2. Additional Methods and Results

## Methods

|  |
| --- |
| *a, b are scores of Health States A and B that respondents rated respectively. Full health equals to 10 |

### Figure A1. Data selection process in the indirect approach

| $U_{B}=2$ |  |  |  |  |
| --- | --- | --- | --- | --- |
| $U_{F}=10$  ${p_{B}}^{*}=0.25$ |  | $E\left[ U\left( the gamble option \right) \right]$  $=0.25\times2+(1-0.25)\times10=8$ |  |  |
|  |  |  |  | $Risk premium=8-4=4$ |
| $U_{A}=4$ |  | $U\left( the certain option \right)=4$ |  |  |

### Figure A2. An example of risk premium calculations

## Descriptive statistics for risk measures in the indirect approach

### Table A1. Two-sample t test in estimating differences of risk indicators in two versions

| Variables | Observations | Mean | SD |
| --- | --- | --- | --- |
| Risk premium in Version 1 | 1,307 | 2.40 | 2.27 |
| Risk premium in Version 2 | 1,305 | 2.67 | 2.16 |
| Combined risk premium | 2,612 | 2.53 | 2.22 |
| t value | -3.10 |  |  |
| P value (two-tailed) | 0.002 |  |  |

### Table A2. Skewness/Kurtosis test for Normality

| Variable | Observations | P value (Skewness) | P value (Kurtosis) | P value (Normality) |
| --- | --- | --- | --- | --- |
| Risk premium in Version 1 | 1,307 | 0.0283 | 0.9525 | 0.091 |
| Risk premium in Version 2 | 1,305 | 0.0015 | 0.6273 | 0.007 |
| Adjusted risk premium | 2,612 | 0.0002 | 0.7873 | 0.001 |

## Empirical results: comparison between the indirect and direct approach

### Table A3. Associations of any risky behaviour with the direct and indirect risk indicators

|  | Dependent variable: Any risky behaviour | | | | | | | | |
| --- | --- | --- | --- | --- | --- | --- | --- | --- | --- |
|  | Indirect approach [model] | | | Direct approach (general) [model] | | | Direct approach (health) [model] | | |
|  | [1] | [2] | [3] | [1] | [2] | [3] | [1] | [2] | [3] |
| Risk preferences |  |  |  |  |  |  |  |  |  |
| The risk indicator | 0.029 | 0.033 | 0.040 | 0.180*** | 0.172*** | 0.132*** | 0.213*** | 0.207*** | 0.191*** |
|  | (0.026) | (0.027) | (0.028) | (0.027) | (0.029) | (0.031) | (0.028) | (0.030) | (0.031) |
| Socio-demographics |  |  |  |  |  |  |  |  |  |
| Age(years) | 0.031*** | 0.026** | 0.030*** | 0.035*** | 0.029*** | 0.032*** | 0.031*** | 0.025** | 0.027** |
|  | (0.009) | (0.010) | (0.011) | (0.009) | (0.010) | (0.011) | (0.009) | (0.011) | (0.011) |
| Age squared/100 | -0.027*** | -0.021* | -0.026** | -0.029*** | -0.022** | -0.026** | -0.023** | -0.017 | -0.020* |
|  | (0.010) | (0.011) | (0.011) | (0.010) | (0.011) | (0.011) | (0.010) | (0.011) | (0.011) |
| Male | 0.462*** | 0.439*** | 0.436*** | 0.405*** | 0.389*** | 0.401*** | 0.418*** | 0.396*** | 0.406*** |
|  | (0.052) | (0.056) | (0.058) | (0.053) | (0.056) | (0.059) | (0.053) | (0.056) | (0.058) |
| White | 0.346*** | 0.314*** | 0.313*** | 0.390*** | 0.355*** | 0.341*** | 0.362*** | 0.327*** | 0.328*** |
|  | (0.100) | (0.106) | (0.108) | (0.100) | (0.106) | (0.107) | (0.100) | (0.106) | (0.108) |
| Born in UK | 0.241** | 0.223** | 0.208** | 0.271*** | 0.251** | 0.231** | 0.227** | 0.208** | 0.192* |
|  | (0.096) | (0.103) | (0.103) | (0.096) | (0.104) | (0.104) | (0.096) | (0.103) | (0.103) |
| Christian |  | -0.114** | -0.136** |  | -0.112** | -0.129** |  | -0.109* | -0.134** |
|  |  | (0.056) | (0.057) |  | (0.056) | (0.057) |  | (0.056) | (0.057) |
| Higher education |  | -0.082 | -0.095* |  | -0.104* | -0.104* |  | -0.095* | -0.101* |
|  |  | (0.056) | (0.057) |  | (0.057) | (0.057) |  | (0.057) | (0.057) |
| Unemployed |  | -0.388*** | -0.390*** |  | -0.379*** | -0.386*** |  | -0.385*** | -0.383*** |
|  |  | (0.138) | (0.140) |  | (0.139) | (0.140) |  | (0.140) | (0.141) |
| Household income |  | 0.067*** | 0.055*** |  | 0.057*** | 0.051** |  | 0.067*** | 0.057*** |
|  |  | (0.021) | (0.021) |  | (0.021) | (0.021) |  | (0.020) | (0.021) |
| Sick or disabled |  | -0.205 | -0.195 |  | -0.140 | -0.153 |  | -0.126 | -0.123 |
|  |  | (0.136) | (0.138) |  | (0.135) | (0.137) |  | (0.137) | (0.138) |
| Married/partnered |  | 0.098 | 0.104* |  | 0.100* | 0.101* |  | 0.098 | 0.099 |
|  |  | (0.060) | (0.060) |  | (0.060) | (0.060) |  | (0.060) | (0.060) |
| Own self-related health(0-10) |  | -0.015 | -0.019 |  | -0.017 | -0.017 |  | -0.007 | -0.011 |
|  |  | (0.016) | (0.016) |  | (0.016) | (0.016) |  | (0.016) | (0.016) |
| Personality traits |  |  |  |  |  |  |  |  |  |
| Extraversion |  |  | 0.086*** |  |  | 0.071*** |  |  | 0.077*** |
|  |  |  | (0.015) |  |  | (0.015) |  |  | (0.015) |
| Agreeableness |  |  | -0.019 |  |  | -0.020 |  |  | -0.013 |
|  |  |  | (0.017) |  |  | (0.017) |  |  | (0.017) |
| Conscientiousness |  |  | -0.019 |  |  | -0.019 |  |  | 0.001 |
|  |  |  | (0.017) |  |  | (0.017) |  |  | (0.017) |
| Neuroticism |  |  | -0.013 |  |  | -0.001 |  |  | -0.006 |
|  |  |  | (0.014) |  |  | (0.015) |  |  | (0.015) |
| Openness |  |  | -0.000 |  |  | -0.008 |  |  | -0.003 |
|  |  |  | (0.016) |  |  | (0.016) |  |  | (0.016) |
| Estimators |  |  |  |  |  |  |  |  |  |
| Pseudo R2 | 0.047 | 0.059 | 0.072 | 0.060 | 0.069 | 0.077 | 0.064 | 0.074 | 0.084 |
| Log pseudo-likelihood | -1665 | -1509 | -1487 | -1643 | -1492 | -1479 | -1637 | -1485 | -1468 |
| AIC | 3344 | 3046 | 3012 | 3301 | 3011 | 2996 | 3287 | 2997 | 2974 |
| BIC | 3385 | 3127 | 3121 | 3342 | 3092 | 3106 | 3328 | 3078 | 3084 |
| *Observations* | 2,572 | 2,367 | 2,367 | 2,572 | 2,367 | 2,367 | 2,572 | 2,367 | 2,367 |
| Notes: Any risky behaviour is a binary variable which equals to 1 if the respondent has any of the three risky health behaviours and equals to 0 otherwise. Coefficients of standardised risk indicators were reported;***, **, * represent significance level at the 1%, 5% and 10% significance level, respectively; robust standard errors are shown in parentheses. | | | | | | | | | |

### Table A4. Associations of number of risky behaviours with the direct and indirect risk indicators

|  | Dependent variable: Number of risky behaviours | | | | | | | | |
| --- | --- | --- | --- | --- | --- | --- | --- | --- | --- |
|  | Indirect approach [model] | | | Direct approach (general) [model] | | | Direct approach (health) [model] | | |
|  | [1] | [2] | [3] | [1] | [2] | [3] | [1] | [2] | [3] |
| *Risk preferences* |  |  |  |  |  |  |  |  |  |
| The risk indicator | 0.027 | 0.030 | 0.034 | 0.164*** | 0.166*** | 0.124*** | 0.222*** | 0.219*** | 0.204*** |
|  | (0.023) | (0.024) | (0.024) | (0.025) | (0.026) | (0.028) | (0.026) | (0.027) | (0.027) |
| *Socio-demographics* |  |  |  |  |  |  |  |  |  |
| Age(years) | 0.034*** | 0.030*** | 0.034*** | 0.037*** | 0.033*** | 0.036*** | 0.033*** | 0.029*** | 0.030*** |
|  | (0.009) | (0.009) | (0.010) | (0.009) | (0.010) | (0.010) | (0.009) | (0.010) | (0.010) |
| Age squared/100 | -0.036*** | -0.032*** | -0.037*** | -0.037*** | -0.033*** | -0.037*** | -0.032*** | -0.028*** | -0.030*** |
|  | (0.009) | (0.010) | (0.010) | (0.009) | (0.010) | (0.010) | (0.009) | (0.010) | (0.010) |
| Male | 0.446*** | 0.432*** | 0.424*** | 0.393*** | 0.384*** | 0.392*** | 0.399*** | 0.387*** | 0.394*** |
|  | (0.047) | (0.050) | (0.051) | (0.048) | (0.051) | (0.052) | (0.048) | (0.051) | (0.052) |
| White | 0.271** | 0.252** | 0.259** | 0.309*** | 0.291*** | 0.284** | 0.287*** | 0.268** | 0.275** |
|  | (0.106) | (0.111) | (0.113) | (0.106) | (0.110) | (0.111) | (0.107) | (0.112) | (0.113) |
| Born in UK | 0.271*** | 0.249*** | 0.241** | 0.297*** | 0.277*** | 0.264*** | 0.259*** | 0.239** | 0.228** |
|  | (0.092) | (0.097) | (0.097) | (0.093) | (0.097) | (0.098) | (0.092) | (0.096) | (0.097) |
| Christian |  | -0.077 | -0.099** |  | -0.072 | -0.090* |  | -0.069 | -0.094* |
|  |  | (0.049) | (0.049) |  | (0.049) | (0.050) |  | (0.049) | (0.050) |
| Higher education |  | -0.116** | -0.121** |  | -0.135*** | -0.130*** |  | -0.127** | -0.128** |
|  |  | (0.049) | (0.050) |  | (0.050) | (0.050) |  | (0.050) | (0.050) |
| Unemployed |  | -0.369** | -0.370** |  | -0.356** | -0.364** |  | -0.363** | -0.358** |
|  |  | (0.143) | (0.144) |  | (0.143) | (0.144) |  | (0.143) | (0.144) |
| Household income |  | 0.038** | 0.027* |  | 0.028* | 0.023 |  | 0.035** | 0.028* |
|  |  | (0.016) | (0.016) |  | (0.016) | (0.016) |  | (0.016) | (0.016) |
| Sick or disabled |  | -0.237* | -0.234* |  | -0.173 | -0.192 |  | -0.151 | -0.155 |
|  |  | (0.135) | (0.139) |  | (0.135) | (0.138) |  | (0.137) | (0.140) |
| Married/partnered |  | 0.048 | 0.057 |  | 0.048 | 0.053 |  | 0.051 | 0.054 |
|  |  | (0.054) | (0.054) |  | (0.054) | (0.054) |  | (0.054) | (0.054) |
| Own self-related health(0-10) |  | -0.030** | -0.035** |  | -0.032** | -0.033** |  | -0.022 | -0.027* |
|  |  | (0.014) | (0.014) |  | (0.014) | (0.014) |  | (0.014) | (0.014) |
| *Personality traits* |  |  |  |  |  |  |  |  |  |
| Extraversion |  |  | 0.077*** |  |  | 0.062*** |  |  | 0.067*** |
|  |  |  | (0.013) |  |  | (0.013) |  |  | (0.013) |
| Agreeableness |  |  | 0.002 |  |  | 0.002 |  |  | 0.009 |
|  |  |  | (0.015) |  |  | (0.015) |  |  | (0.015) |
| Conscientiousness |  |  | -0.029* |  |  | -0.030** |  |  | -0.007 |
|  |  |  | (0.015) |  |  | (0.015) |  |  | (0.015) |
| Neuroticism |  |  | -0.018 |  |  | -0.006 |  |  | -0.011 |
|  |  |  | (0.013) |  |  | (0.013) |  |  | (0.013) |
| Openness |  |  | -0.001 |  |  | -0.008 |  |  | -0.003 |
|  |  |  | (0.014) |  |  | (0.014) |  |  | (0.014) |
| *Estimators* |  |  |  |  |  |  |  |  |  |
| Pseudo R^2^ | 0.030 | 0.036 | 0.047 | 0.039 | 0.044 | 0.050 | 0.046 | 0.051 | 0.059 |
| Log pseudo-likelihood | -2454 | -2262 | -2237 | -2432 | -2242 | -2228 | -2415 | -2227 | -2209 |
| AIC | 4927 | 4557 | 4516 | 4882 | 4515 | 4497 | 4848 | 4486 | 4459 |
| BIC | 4979 | 4649 | 4637 | 4934 | 4608 | 4618 | 4901 | 4579 | 4580 |
| *Observations* | 2,560 | 2,355 | 2,355 | 2,560 | 2,355 | 2,355 | 2,560 | 2,355 | 2,355 |
| Notes: Number of risky behaviours is a categorical variable which ranges from 0 to 3; 0 represents the respondent didn’t have risky behaviours and 3 represents the respondents have three risky health behaviours. Coefficients of standardised risk indicators were reported;***, **, * represent significance level at the 1%, 5% and 10% significance level, respectively; robust standard errors are shown in parentheses. | | | | | | | | | |

### Table A5. Associations of smoking status with the direct and indirect risk indicators

|  | Dependent variable: smoking status | | | | | | | | | | | | | | | | |
| --- | --- | --- | --- | --- | --- | --- | --- | --- | --- | --- | --- | --- | --- | --- | --- | --- | --- |
|  | Indirect approach [model] | | | | | Direct approach (general) [model] | | | | | | Direct approach (health) [model] | | | | | |
|  | [1] | | [2] | | [3] | [1] | | [2] | | [3] | | [1] | | [2] | | [3] |  |
| *Risk preferences* |  | |  | |  |  | |  | |  | |  | |  | |  |  |
| The risk indicator | 0.030 | | 0.030 | | 0.034 | 0.116*** | | 0.170*** | | 0.120*** | | 0.226*** | | 0.233*** | | 0.233*** |  |
|  | (0.027) | | (0.028) | | (0.029) | (0.030) | | (0.032) | | (0.035) | | (0.030) | | (0.031) | | (0.032) |  |
| *Socio-demographics* | |  | |  | | |  | |  | |  | |  | |  | |  |
| Age(years) | 0.054*** | | 0.053*** | | 0.054*** | 0.056*** | | 0.055*** | | 0.056*** | | 0.053*** | | 0.051*** | | 0.049*** |  |
|  | (0.010) | | (0.012) | | (0.012) | (0.010) | | (0.012) | | (0.012) | | (0.010) | | (0.012) | | (0.012) |  |
| Age squared/100 | -0.075*** | | -0.075*** | | -0.079*** | -0.076*** | | -0.076*** | | -0.079*** | | -0.071*** | | -0.071*** | | -0.071*** |  |
|  | (0.011) | | (0.013) | | (0.013) | (0.011) | | (0.013) | | (0.013) | | (0.011) | | (0.013) | | (0.013) |  |
| Male | 0.275*** | | 0.293*** | | 0.296*** | 0.238*** | | 0.245*** | | 0.266*** | | 0.224*** | | 0.241*** | | 0.259*** |  |
|  | (0.056) | | (0.059) | | (0.061) | (0.057) | | (0.06) | | (0.062) | | (0.056) | | (0.060) | | (0.062) |  |
| White | 0.210** | | 0.185 | | 0.206* | 0.240** | | 0.228** | | 0.234** | | 0.238** | | 0.218* | | 0.244** |  |
|  | (0.106) | | (0.113) | | (0.115) | (0.105) | | (0.112) | | (0.114) | | (0.107) | | (0.115) | | (0.117) |  |
| Born in UK | 0.178* | | 0.141 | | 0.123 | 0.192* | | 0.166 | | 0.145 | | 0.156 | | 0.119 | | 0.099 |  |
|  | (0.103) | | (0.110) | | (0.112) | (0.103) | | (0.111) | | (0.112) | | (0.103) | | (0.111) | | (0.112) |  |
| Christian |  | | 0.043 | | 0.012 |  | | 0.052 | | 0.023 | |  | | 0.055 | | 0.023 |  |
|  |  | | (0.059) | | (0.059) |  | | (0.059) | | (0.059) | |  | | (0.059) | | (0.060) |  |
| Higher education |  | | -0.309*** | | -0.312*** |  | | -0.327*** | | -0.319*** | |  | | -0.324*** | | -0.321*** |  |
|  |  | | (0.060) | | (0.061) |  | | (0.06) | | (0.061) | |  | | (0.060) | | (0.061) |  |
| Unemployed |  | | -0.013 | | 0.001 |  | | 0.005 | | 0.008 | |  | | 0.000 | | 0.022 |  |
|  |  | | (0.139) | | (0.137) |  | | (0.14) | | (0.138) | |  | | (0.139) | | (0.137) |  |
| Household income |  | | -0.016 | | -0.023 |  | | -0.026 | | -0.026 | |  | | -0.019 | | -0.022 |  |
|  |  | | (0.022) | | (0.022) |  | | (0.022) | | (0.022) | |  | | (0.022) | | (0.022) |  |
| Sick or disabled |  | | 0.014 | | 0.022 |  | | 0.088 | | 0.067 | |  | | 0.117 | | 0.127 |  |
|  |  | | (0.146) | | (0.153) |  | | (0.147) | | (0.152) | |  | | (0.149) | | (0.154) |  |
| Married/partnered |  | | -0.043 | | -0.041 |  | | -0.04 | | -0.04 | |  | | -0.039 | | -0.042 |  |
|  |  | | (0.065) | | (0.066) |  | | (0.066) | | (0.066) | |  | | (0.066) | | (0.066) |  |
| Own self-related health(0-10) |  | | -0.098*** | | -0.105*** |  | | -0.100*** | | -0.103*** | |  | | -0.091*** | | -0.097*** |  |
|  |  | | (0.017) | | (0.017) |  | | (0.017) | | (0.017) | |  | | (0.017) | | (0.017) |  |
| *Personality traits* |  | |  | |  |  | |  | |  | |  | |  | |  |  |
| Extraversion |  | |  | | 0.071*** |  | |  | | 0.057*** | |  | |  | | 0.059*** |  |
|  |  | |  | | (0.016) |  | |  | | (0.016) | |  | |  | | (0.016) |  |
| Agreeableness |  | |  | | 0.055*** |  | |  | | 0.054*** | |  | |  | | 0.064*** |  |
|  |  | |  | | (0.019) |  | |  | | (0.019) | |  | |  | | (0.019) |  |
| Conscientiousness |  | |  | | -0.019 |  | |  | | -0.02 | |  | |  | | 0.008 |  |
|  |  | |  | | (0.018) |  | |  | | (0.018) | |  | |  | | (0.019) |  |
| Neuroticism |  | |  | | -0.018 |  | |  | | -0.008 | |  | |  | | -0.012 |  |
|  |  | |  | | (0.016) |  | |  | | (0.017) | |  | |  | | (0.016) |  |
| Openness |  | |  | | 0.004 |  | |  | | -0.003 | |  | |  | | 0.001 |  |
|  |  | |  | | (0.017) |  | |  | | (0.017) | |  | |  | | (0.017) |  |
| *Estimators* |  | |  | |  |  | |  | |  | |  | |  | |  |  |
| Pseudo R^2^ | 0.037 | | 0.060 | | 0.074 | 0.041 | | 0.069 | | 0.077 | | 0.054 | | 0.077 | | 0.090 |  |
| Log pseudo-likelihood | -1742 | | -1591 | | -1568 | -1734 | | -1577 | | -1563 | | -1712 | | -1563 | | -1542 |  |
| AIC | 3500 | | 3212 | | 3177 | 3484 | | 3183 | | 3165 | | 3440 | | 3155 | | 3124 |  |
| BIC | 3546 | | 3299 | | 3292 | 3531 | | 3270 | | 3281 | | 3487 | | 3242 | | 3239 |  |
| *Observations* | 2,597 | | 2,387 | | 2,387 | 2,597 | | 2,387 | | 2,387 | | 2,597 | | 2,387 | | 2,387 |  |

Notes: The smoking status measures the frequency of smoking, where non-smokers, occasional smokers and regular smokers are represented by 1, 2 and 3 separately. The direct approach contains two risk indicators, one is the score asking people willingness to take risks in general domain, another one is the score in the health domain. Coefficients of standardised risk indicators were reported;***, **, * represent significance level at the 1%, 5% and 10% significance level, respectively; robust standard errors are shown in parentheses.

### Table A6. Associations of regular smoking with the direct and indirect risk indicators

|  | Dependent variable: regular smoker | | | | | | | | | | | | | | | |
| --- | --- | --- | --- | --- | --- | --- | --- | --- | --- | --- | --- | --- | --- | --- | --- | --- |
|  | Indirect approach [model] | | | | | Direct approach (general) [model] | | | | | Direct approach (health) [model] | | | | | |
|  | [1] | [2] | | [3] | | [1] | | [2] | [3] | | [1] | | [2] | | [3] |  |
| *Risk preferences* | |  | |  | |  | |  |  | |  | |  | |  |  |
| The risk indicator | 0.050* | 0.053* | | 0.060* | | 0.099*** | | 0.160*** | 0.113*** | | 0.229*** | | 0.245*** | | 0.257*** |  |
|  | (0.029) | (0.031) | | (0.031) | | (0.032) | | (0.035) | (0.038) | | (0.033) | | (0.035) | | (0.036) |  |
| *Socio-demographics* | | |  | |  | |  | | |  | |  | |  | |  |
| Age(years) | 0.074*** | 0.073*** | | 0.072*** | | 0.075*** | | 0.074*** | 0.073*** | | 0.073*** | | 0.072*** | | 0.067*** |  |
|  | (0.011) | (0.013) | | (0.013) | | (0.011) | | (0.013) | (0.013) | | (0.011) | | (0.013) | | (0.013) |  |
| Age squared/100 | -0.091*** | -0.091*** | | -0.092*** | | -0.091*** | | -0.091*** | -0.092*** | | -0.088*** | | -0.087*** | | -0.085*** |  |
|  | (0.012) | (0.014) | | (0.014) | | (0.012) | | (0.014) | (0.014) | | (0.012) | | (0.014) | | (0.014) |  |
| Male | 0.243*** | 0.261*** | | 0.272*** | | 0.212*** | | 0.216*** | 0.244*** | | 0.193*** | | 0.210*** | | 0.237*** |  |
|  | (0.060) | (0.064) | | (0.067) | | (0.061) | | (0.066) | (0.068) | | (0.061) | | (0.066) | | (0.068) |  |
| White | 0.165 | 0.176 | | 0.200 | | 0.194 | | 0.220* | 0.228* | | 0.185 | | 0.200 | | 0.234* |  |
|  | (0.122) | (0.132) | | (0.133) | | (0.121) | | (0.132) | (0.133) | | (0.124) | | (0.135) | | (0.137) |  |
| Born in UK | 0.288** | 0.228* | | 0.215 | | 0.297** | | 0.250* | 0.234* | | 0.262** | | 0.205 | | 0.190 |  |
|  | (0.124) | (0.132) | | (0.133) | | (0.124) | | (0.133) | (0.133) | | (0.124) | | (0.133) | | (0.135) |  |
| Christian |  | -0.012 | | -0.044 | |  | | -0.005 | -0.035 | |  | | -0.002 | | -0.036 |  |
|  |  | (0.065) | | (0.066) | |  | | (0.065) | (0.066) | |  | | (0.065) | | (0.066) |  |
| Higher education |  | -0.356*** | | -0.355*** | |  | | -0.368*** | -0.358*** | |  | | -0.372*** | | -0.365*** |  |
|  |  | (0.066) | | (0.068) | |  | | (0.067) | (0.068) | |  | | (0.067) | | (0.068) |  |
| Unemployed |  | 0.045 | | 0.070 | |  | | 0.059 | 0.074 | |  | | 0.055 | | 0.093 |  |
|  |  | (0.146) | | (0.145) | |  | | (0.148) | (0.147) | |  | | (0.147) | | (0.146) |  |
| Household income |  | -0.029 | | -0.038 | |  | | -0.038 | -0.040 | |  | | -0.035 | | -0.040 |  |
|  |  | (0.025) | | (0.025) | |  | | (0.025) | (0.025) | |  | | (0.025) | | (0.026) |  |
| Sick or disabled |  | 0.049 | | 0.060 | |  | | 0.124 | 0.108 | |  | | 0.163 | | 0.181 |  |
|  |  | (0.147) | | (0.152) | |  | | (0.148) | (0.152) | |  | | (0.150) | | (0.154) |  |
| Married/partnered |  | -0.055 | | -0.051 | |  | | -0.052 | -0.051 | |  | | -0.050 | | -0.052 |  |
|  |  | (0.071) | | (0.072) | |  | | (0.072) | (0.072) | |  | | (0.072) | | (0.072) |  |
| Own self-related health(0-10) |  | -0.102*** | | -0.109*** | |  | | -0.104*** | -0.107*** | |  | | -0.095*** | | -0.102*** |  |
|  |  | (0.018) | | (0.018) | |  | | (0.018) | (0.018) | |  | | (0.018) | | (0.018) |  |
| *Personality traits* | | | | | | | | | | | | | | | |  |
| Extraversion |  |  | | 0.065*** | |  | |  | 0.050*** | |  | |  | | 0.051*** |  |
|  |  |  | | (0.017) | |  | |  | (0.018) | |  | |  | | (0.017) |  |
| Agreeableness |  |  | | 0.050** | |  | |  | 0.051** | |  | |  | | 0.061*** |  |
|  |  |  | | (0.021) | |  | |  | (0.021) | |  | |  | | (0.021) |  |
| Conscientiousness |  |  | | -0.001 | |  | |  | -0.003 | |  | |  | | 0.029 |  |
|  |  |  | | (0.020) | |  | |  | (0.020) | |  | |  | | (0.021) |  |
| Neuroticism |  |  | | -0.016 | |  | |  | -0.007 | |  | |  | | -0.011 |  |
|  |  |  | | (0.017) | |  | |  | (0.018) | |  | |  | | (0.018) |  |
| Openness |  |  | | 0.002 | |  | |  | -0.005 | |  | |  | | -0.003 |  |
|  |  |  | | (0.019) | |  | |  | (0.019) | |  | |  | | (0.019) |  |
| *Estimators* |  |  | |  | |  | |  |  | |  | |  | |  |  |
| Pseudo R^2^ | 0.043 | 0.080 | | 0.094 | | 0.046 | | 0.088 | 0.097 | | 0.063 | | 0.102 | | 0.116 |  |
| Log pseudo-likelihood | -1171 | -1057 | | -1040 | | -1168 | | -1047 | -1037 | | -1146 | | -1031 | | -1015 |  |
| AIC | 2356 | 2141 | | 2118 | | 2349 | | 2122 | 2112 | | 2307 | | 2091 | | 2067 |  |
| BIC | 2397 | 2222 | | 2228 | | 2390 | | 2202 | 2222 | | 2348 | | 2171 | | 2177 |  |
| *Observations* | 2,597 | 2,387 | | 2,387 | | 2,597 | | 2,387 | 2,387 | | 2,597 | | 2,387 | | 2,387 |  |
| Notes: Regular smoker is a binary variable which equals to 1 if the respondent is a regular smoker and equals to 0 otherwise. Coefficients of standardised risk indicators were reported;***, **, * represent significance level at the 1%, 5% and 10% significance level, respectively; robust standard errors are shown in parentheses. | | | | | | | | | | | | | | | |  |

### Table A7. Associations of non-adherence to taking a full course of antibiotics with the direct and indirect risk indicators

|  | Dependent variable : Non-adherence to taking a full course of antibiotics | | | | | | | | | |
| --- | --- | --- | --- | --- | --- | --- | --- | --- | --- | --- |
|  | Indirect approach [model] | | | Direct approach (general) [model] | | | Direct approach (health) [model] | | | |
|  | [1] | [2] | [3] | [1] | [2] | [3] | [1] | [2] | [3] |  |
| *Risk preferences* |  |  |  |  |  |  |  |  |  |  |
| The risk indicator | 0.064** | 0.064** | 0.058* | 0.104*** | 0.122*** | 0.148*** | 0.252*** | 0.258*** | 0.234*** |  |
|  | (0.030) | (0.031) | (0.032) | (0.032) | (0.034) | (0.038) | (0.031) | (0.032) | (0.033) |  |
| *Socio-demographics* | |  |  |  |  |  |  |  |  |  |
| Age(years) | -0.028*** | -0.029*** | -0.021* | -0.028*** | -0.029** | -0.021* | -0.032*** | -0.034*** | -0.028** |  |
|  | (0.010) | (0.011) | (0.012) | (0.01) | (0.011) | (0.012) | (0.01) | (0.011) | (0.012) |  |
| Age squared/100 | 0.008 | 0.008 | 0.001 | 0.008 | 0.009 | 0.002 | 0.015 | 0.016 | 0.010 |  |
|  | (0.011) | (0.012) | (0.013) | (0.011) | (0.012) | (0.013) | (0.011) | (0.013) | (0.013) |  |
| Male | 0.364*** | 0.411*** | 0.359*** | 0.335*** | 0.380*** | 0.325*** | 0.308*** | 0.352*** | 0.319*** |  |
|  | (0.058) | (0.061) | (0.062) | (0.059) | (0.062) | (0.063) | (0.058) | (0.062) | (0.063) |  |
| White | -0.202** | -0.222** | -0.239** | -0.181* | -0.197* | -0.217** | -0.185* | -0.202** | -0.221** |  |
|  | (0.095) | (0.102) | (0.101) | (0.095) | (0.102) | (0.102) | (0.095) | (0.102) | (0.102) |  |
| Born in UK | -0.373*** | -0.439*** | -0.438*** | -0.365*** | -0.427*** | -0.418*** | -0.423*** | -0.490*** | -0.484*** |  |
|  | (0.088) | (0.093) | (0.094) | (0.088) | (0.093) | (0.093) | (0.089) | (0.094) | (0.094) |  |
| Christian |  | 0.015 | 0.041 |  | 0.020 | 0.052 |  | 0.033 | 0.057 |  |
|  |  | (0.060) | (0.061) |  | (0.060) | (0.061) |  | (0.061) | (0.061) |  |
| Higher education |  | -0.129** | -0.138** |  | -0.136** | -0.143** |  | -0.137** | -0.139** |  |
|  |  | (0.061) | (0.061) |  | (0.061) | (0.062) |  | (0.061) | (0.062) |  |
| Unemployed |  | -0.177 | -0.214 |  | -0.161 | -0.206 |  | -0.152 | -0.189 |  |
|  |  | (0.135) | (0.134) |  | (0.136) | (0.134) |  | (0.137) | (0.137) |  |
| Household income |  | -0.030 | -0.041* |  | -0.036* | -0.044** |  | -0.029 | -0.037* |  |
|  |  | (0.021) | (0.021) |  | (0.021) | (0.021) |  | (0.022) | (0.022) |  |
| Sick or disabled |  | -0.167 | -0.151 |  | -0.113 | -0.102 |  | -0.043 | -0.045 |  |
|  |  | (0.185) | (0.187) |  | (0.183) | (0.186) |  | (0.186) | (0.188) |  |
| Married/partnered |  | 0.040 | 0.046 |  | 0.041 | 0.047 |  | 0.039 | 0.043 |  |
|  |  | (0.066) | (0.067) |  | (0.066) | (0.067) |  | (0.067) | (0.068) |  |
| Own self-related health(0-10) |  | -0.002 | 0.002 |  | -0.004 | 0.004 |  | 0.010 | 0.013 |  |
|  |  | (0.018) | (0.018) |  | (0.018) | (0.019) |  | (0.018) | (0.019) |  |
| *Personality traits* | |  |  |  |  |  |  |  |  |  |
| Extraversion |  |  | 0.019 |  |  | 0.001 |  |  | 0.005 |  |
|  |  |  | (0.017) |  |  | (0.017) |  |  | (0.017) |  |
| Agreeableness |  |  | -0.078*** |  |  | -0.079*** |  |  | -0.072*** |  |
|  |  |  | (0.020) |  |  | (0.02) |  |  | (0.02) |  |
| Conscientiousness |  |  | -0.077*** |  |  | -0.079*** |  |  | -0.053*** |  |
|  |  |  | (0.019) |  |  | (0.019) |  |  | (0.019) |  |
| Neuroticism |  |  | -0.020 |  |  | -0.007 |  |  | -0.010 |  |
|  |  |  | (0.017) |  |  | (0.017) |  |  | (0.017) |  |
| Openness |  |  | -0.003 |  |  | -0.011 |  |  | -0.005 |  |
|  |  |  | (0.017) |  |  | (0.018) |  |  | (0.017) |  |
| *Estimators* |  |  |  |  |  |  |  |  |  |  |
| Pseudo R^2^ | 0.064 | 0.070 | 0.084 | 0.066 | 0.074 | 0.088 | 0.083 | 0.090 | 0.099 |  |
| Log pseudo-likelihood | -1618 | -1480 | -1459 | -1615 | -1475 | -1452 | -1586 | -1449 | -1435 |  |
| AIC | 3255 | 2993 | 2960 | 3248 | 2982 | 2945 | 3190 | 2930 | 2912 |  |
| BIC | 3308 | 3085 | 3081 | 3300 | 3074 | 3066 | 3242 | 3022 | 3033 |  |
| *Observations* | 2,560 | 2,355 | 2,355 | 2,560 | 2,355 | 2,355 | 2,560 | 2,355 | 2,355 |  |
| Notes: There are four degrees whether respondents would take the full course or not: definitely, probably, probably not and definitely not. These are  measured on a scale from 1 to 4. Coefficients of standardised risk indicators were reported;***, **, * represent significance level at the 1%, 5% and 10% significance level, respectively; robust standard errors are shown in parentheses. | | | | | | | | | |  |

## Empirical results: comparison between two versions in the indirect approach

### Table A8. Determinants of any risky behaviour in the indirect approach

|  | Dependent variable: Any risky behaviour | | | | | |
| --- | --- | --- | --- | --- | --- | --- |
|  | Version 1 [model] | | | Version 2 [model] | | |
|  | [1] | [2] | [3] | [1] | [2] | [3] |
| *Risk preferences* |  |  |  |  |  |  |
| Risk indicator | 0.051 | 0.059 | 0.060 | 0.006 | 0.003 | 0.014 |
|  | (0.037) | (0.039) | (0.040) | (0.037) | (0.039) | (0.040) |
| *Socio-demographics* |  |  |  |  |  |  |
| Age(years) | 0.027** | 0.020 | 0.026* | 0.035*** | 0.033** | 0.035** |
|  | (0.013) | (0.015) | (0.016) | (0.013) | (0.014) | (0.015) |
| Age squared/100 | -0.021 | -0.015 | -0.022 | -0.032** | -0.028* | -0.032** |
|  | (0.014) | (0.016) | (0.016) | (0.014) | (0.015) | (0.015) |
| Male | 0.433*** | 0.422*** | 0.420*** | 0.490*** | 0.456*** | 0.460*** |
|  | (0.074) | (0.079) | (0.082) | (0.075) | (0.078) | (0.082) |
| White | 0.362** | 0.309** | 0.315** | 0.332** | 0.317** | 0.332** |
|  | (0.141) | (0.153) | (0.155) | (0.141) | (0.148) | (0.151) |
| Born in UK | 0.306** | 0.326** | 0.285* | 0.183 | 0.137 | 0.137 |
|  | (0.138) | (0.152) | (0.150) | (0.134) | (0.141) | (0.142) |
| Christian |  | -0.063 | -0.077 |  | -0.172** | -0.206** |
|  |  | (0.080) | (0.081) |  | (0.079) | (0.081) |
| Higher education |  | -0.028 | -0.046 |  | -0.144* | -0.153* |
|  |  | (0.081) | (0.081) |  | (0.079) | (0.081) |
| Unemployed |  | -0.437** | -0.457** |  | -0.318 | -0.325 |
|  |  | (0.179) | (0.184) |  | (0.217) | (0.215) |
| Household income |  | 0.081*** | 0.073** |  | 0.058** | 0.046* |
|  |  | (0.031) | (0.032) |  | (0.027) | (0.028) |
| Sick or disabled |  | -0.273 | -0.292 |  | -0.126 | -0.074 |
|  |  | (0.186) | (0.191) |  | (0.201) | (0.203) |
| Married/partnered |  | 0.142 | 0.153* |  | 0.048 | 0.055 |
|  |  | (0.087) | (0.087) |  | (0.083) | (0.084) |
| Own self-related health(0-10) |  | -0.011 | -0.017 |  | -0.018 | -0.024 |
|  |  | (0.023) | (0.024) |  | (0.022) | (0.023) |
| *Personality traits* |  |  |  |  |  |  |
| Extraversion |  |  | 0.094*** |  |  | 0.080*** |
|  |  |  | (0.022) |  |  | (0.021) |
| Agreeableness |  |  | 0.025 |  |  | -0.057** |
|  |  |  | (0.024) |  |  | (0.024) |
| Conscientiousness |  |  | -0.063** |  |  | 0.023 |
|  |  |  | (0.024) |  |  | (0.024) |
| Neuroticism |  |  | -0.014 |  |  | -0.011 |
|  |  |  | (0.021) |  |  | (0.021) |
| Openness |  |  | -0.008 |  |  | 0.005 |
|  |  |  | (0.023) |  |  | (0.022) |
| *Estimators* |  |  |  |  |  |  |
| Pseudo R^2^ | 0.0473 | 0.0632 | 0.0821 | 0.0482 | 0.0582 | 0.0732 |
| Log pseudo-likelihood | 1679 | 1507 | 1487 | 1677 | 1559 | 1545 |
| AIC | 1715 | 1578 | 1583 | 1713 | 1631 | 1642 |
| BIC | -832.4 | -739.3 | -724.4 | -831.5 | -765.7 | -753.5 |
| *Observations* | 1,291 | 1,172 | 1,172 | 1,281 | 1,195 | 1,195 |
| Notes: Coefficients of standardised risk indicators were reported; ***, **, * represent significant level at the 1%, 5% and 10% significant level, respectively; robust standard errors are shown in parentheses. | | | | | | |

### Table A9. Determinants of number of risky behaviours in the indirect approach

|  | Dependent variable: Number of risky behaviours | | | | | |
| --- | --- | --- | --- | --- | --- | --- |
|  | Version 1 [model] | | | Version 2 [model] | | |
|  | [1] | [2] | [3] | [1] | [2] | [3] |
| *Risk preferences* |  |  |  |  |  |  |
| Risk indicator | 0.063** | 0.067** | 0.067** | -0.012 | -0.010 | -0.003 |
|  | (0.032) | (0.033) | (0.034) | (0.033) | (0.035) | (0.036) |
| *Socio-demographics* |  |  |  |  |  |  |
| Age(years) | 0.033*** | 0.028** | 0.034** | 0.035*** | 0.033** | 0.036*** |
|  | (0.012) | (0.014) | (0.014) | (0.012) | (0.013) | (0.013) |
| Age squared/100 | -0.035*** | -0.030** | -0.037** | -0.036*** | -0.035*** | -0.039*** |
|  | (0.012) | (0.014) | (0.014) | (0.012) | (0.013) | (0.014) |
| Male | 0.411*** | 0.394*** | 0.397*** | 0.482*** | 0.469*** | 0.459*** |
|  | (0.066) | (0.071) | (0.072) | (0.067) | (0.071) | (0.073) |
| White | 0.266* | 0.246 | 0.261 | 0.286** | 0.270* | 0.293** |
|  | (0.158) | (0.169) | (0.172) | (0.142) | (0.145) | (0.147) |
| Born in UK | 0.341** | 0.333** | 0.299** | 0.207* | 0.172 | 0.182 |
|  | (0.138) | (0.151) | (0.152) | (0.123) | (0.124) | (0.126) |
| Christian |  | -0.077 | -0.091 |  | -0.083 | -0.116 |
|  |  | (0.069) | (0.070) |  | (0.070) | (0.071) |
| Higher education |  | -0.068 | -0.075 |  | -0.164** | -0.173** |
|  |  | (0.070) | (0.070) |  | (0.070) | (0.072) |
| Unemployed |  | -0.379* | -0.397** |  | -0.354* | -0.354* |
|  |  | (0.195) | (0.197) |  | (0.206) | (0.205) |
| Household income |  | 0.057** | 0.051** |  | 0.024 | 0.012 |
|  |  | (0.023) | (0.024) |  | (0.022) | (0.022) |
| Sick or disabled |  | -0.325* | -0.341* |  | -0.133 | -0.089 |
|  |  | (0.187) | (0.196) |  | (0.192) | (0.196) |
| Married/partnered |  | 0.078 | 0.089 |  | 0.013 | 0.025 |
|  |  | (0.079) | (0.079) |  | (0.075) | (0.075) |
| Own self-related health(0-10) |  | -0.032 | -0.039* |  | -0.027 | -0.032 |
|  |  | (0.020) | (0.021) |  | (0.020) | (0.020) |
| *Personality traits* |  |  |  |  |  |  |
| Extraversion |  |  | 0.082*** |  |  | 0.072*** |
|  |  |  | (0.018) |  |  | (0.018) |
| Agreeableness |  |  | 0.041* |  |  | -0.032 |
|  |  |  | (0.022) |  |  | (0.021) |
| Conscientiousness |  |  | -0.063*** |  |  | 0.002 |
|  |  |  | (0.020) |  |  | (0.022) |
| Neuroticism |  |  | -0.014 |  |  | -0.022 |
|  |  |  | (0.018) |  |  | (0.018) |
| Openness |  |  | -0.010 |  |  | 0.007 |
|  |  |  | (0.019) |  |  | (0.020) |
| *Estimators* |  |  |  |  |  |  |
| Pseudo R^2^ | 0.0292 | 0.0367 | 0.0515 | 0.0331 | 0.0380 | 0.0488 |
| Log pseudo-likelihood | -1233 | -1119 | -1101 | -1216 | -1137 | -1124 |
| AIC | 2484 | 2269 | 2245 | 2451 | 2306 | 2290 |
| BIC | 2531 | 2350 | 2351 | 2497 | 2387 | 2397 |
| *Observations* | 1,284 | 1,165 | 1,165 | 1,276 | 1,190 | 1,190 |
| Notes: Coefficients of standardised risk indicators were reported; ***, **, * represent significant level at the 1%, 5% and 10% significant level, respectively; robust standard errors are shown in parentheses. | | | | | | |

### Table A10. Determinants of smoking status in the indirect approach

|  | Dependent variable: smoking status | | | | | |
| --- | --- | --- | --- | --- | --- | --- |
|  | Version 1 [model] | | | Version 2 [model] | | |
|  | [1] | [2] | [3] | [1] | [2] | [3] |
| *Risk preferences* |  |  |  |  |  |  |
| The risk indicator | 0.060 | 0.059 | 0.056 | -0.001 | -0.001 | 0.004 |
|  | (0.038) | (0.041) | (0.041) | (0.038) | (0.040) | (0.040) |
| *Socio-demographics* |  |  |  |  |  |  |
| Age(years) | 0.053*** | 0.053*** | 0.059*** | 0.057*** | 0.056*** | 0.053*** |
|  | (0.014) | (0.016) | (0.016) | (0.014) | (0.017) | (0.017) |
| Age squared/100 | -0.073*** | -0.074*** | -0.081*** | -0.079*** | -0.079*** | -0.080*** |
|  | (0.015) | (0.017) | (0.018) | (0.016) | (0.018) | (0.019) |
| Male | 0.275*** | 0.283*** | 0.294*** | 0.279*** | 0.307*** | 0.308*** |
|  | (0.078) | (0.083) | (0.085) | (0.079) | (0.085) | (0.088) |
| White | 0.336** | 0.328* | 0.341* | 0.096 | 0.057 | 0.106 |
|  | (0.159) | (0.171) | (0.176) | (0.143) | (0.150) | (0.151) |
| Born in UK | 0.116 | 0.095 | 0.053 | 0.242 | 0.186 | 0.183 |
|  | (0.142) | (0.155) | (0.156) | (0.150) | (0.157) | (0.159) |
| Christian |  | 0.079 | 0.063 |  | -0.000 | -0.056 |
|  |  | (0.082) | (0.083) |  | (0.086) | (0.087) |
| Higher education |  | -0.269*** | -0.282*** |  | -0.348*** | -0.349*** |
|  |  | (0.084) | (0.086) |  | (0.085) | (0.087) |
| Unemployed |  | 0.071 | 0.057 |  | -0.130 | -0.090 |
|  |  | (0.179) | (0.178) |  | (0.225) | (0.223) |
| Household income |  | -0.015 | -0.014 |  | -0.017 | -0.025 |
|  |  | (0.031) | (0.032) |  | (0.030) | (0.030) |
| Sick or disabled |  | -0.115 | -0.129 |  | 0.144 | 0.190 |
|  |  | (0.202) | (0.212) |  | (0.214) | (0.219) |
| Married/partnered |  | -0.052 | -0.046 |  | -0.043 | -0.050 |
|  |  | (0.094) | (0.094) |  | (0.093) | (0.094) |
| Own self-related health(0-10) |  | -0.092*** | -0.100*** |  | -0.105*** | -0.114*** |
|  |  | (0.024) | (0.024) |  | (0.023) | (0.024) |
| *Personality traits* |  |  |  |  |  |  |
| Extraversion |  |  | 0.076*** |  |  | 0.067*** |
|  |  |  | (0.023) |  |  | (0.023) |
| Agreeableness |  |  | 0.075*** |  |  | 0.040 |
|  |  |  | (0.027) |  |  | (0.027) |
| Conscientiousness |  |  | -0.070*** |  |  | 0.032 |
|  |  |  | (0.025) |  |  | (0.027) |
| Neuroticism |  |  | -0.010 |  |  | -0.028 |
|  |  |  | (0.022) |  |  | (0.024) |
| Openness |  |  | 0.009 |  |  | -0.001 |
|  |  |  | (0.024) |  |  | (0.024) |
| *Estimators* |  |  |  |  |  |  |
| Pseudo R^2^ | 0.038 | 0.056 | 0.073 | 0.038 | 0.069 | 0.084 |
| Log pseudo-likelihood | -886 | -805 | -791 | -854 | -783 | -770 |
| AIC | 1787 | 1640 | 1621 | 1725 | 1596 | 1580 |
| BIC | 1829 | 1716 | 1723 | 1766 | 1672 | 1681 |
| *Observations* | 1,302 | 1,180 | 1,180 | 1,295 | 1,207 | 1,207 |

Notes: Coefficients of standardised risk indicators were reported; ***, **, * represent significant level at the 1%, 5% and 10% significant level, respectively; robust standard errors are shown in parentheses.

### Table A11. Determinants of regular smokers in the indirect approach

|  | Dependent variable: regular smoker | | | | | |
| --- | --- | --- | --- | --- | --- | --- |
|  | Version 1 [model] | | | Version 2 [model] | | |
|  | [1] | [2] | [3] | [1] | [2] | [3] |
| *Risk preferences* |  |  |  |  |  |  |
| The risk indicator | 0.106** | 0.108** | 0.110** | -0.008 | -0.010 | -0.003 |
|  | (0.041) | (0.044) | (0.045) | (0.041) | (0.043) | (0.044) |
| *Socio-demographics* |  |  |  |  |  |  |
| Age(years) | 0.076*** | 0.069*** | 0.075*** | 0.074*** | 0.079*** | 0.073*** |
|  | (0.016) | (0.018) | (0.018) | (0.016) | (0.019) | (0.02) |
| Age squared/100 | -0.091*** | -0.084*** | -0.091*** | -0.094*** | -0.101*** | -0.097*** |
|  | (0.017) | (0.019) | (0.020) | (0.018) | (0.021) | (0.021) |
| Male | 0.243*** | 0.235*** | 0.257*** | 0.249*** | 0.290*** | 0.301*** |
|  | (0.086) | (0.091) | (0.094) | (0.085) | (0.092) | (0.095) |
| White | 0.145 | 0.164 | 0.168 | 0.184 | 0.183 | 0.242 |
|  | (0.175) | (0.191) | (0.195) | (0.170) | (0.182) | (0.182) |
| Born in UK | 0.377** | 0.341* | 0.332 | 0.214 | 0.141 | 0.131 |
|  | (0.185) | (0.204) | (0.203) | (0.166) | (0.175) | (0.177) |
| Christian |  | -0.012 | -0.031 |  | -0.014 | -0.070 |
|  |  | (0.091) | (0.092) |  | (0.093) | (0.095) |
| Higher education |  | -0.325*** | -0.332*** |  | -0.381*** | -0.383*** |
|  |  | (0.094) | (0.096) |  | (0.095) | (0.097) |
| Unemployed |  | 0.148 | 0.143 |  | -0.120 | -0.080 |
|  |  | (0.188) | (0.188) |  | (0.239) | (0.237) |
| Household income |  | -0.041 | -0.041 |  | -0.023 | -0.032 |
|  |  | (0.036) | (0.037) |  | (0.034) | (0.035) |
| Sick or disabled |  | -0.011 | -0.029 |  | 0.121 | 0.178 |
|  |  | (0.197) | (0.208) |  | (0.221) | (0.223) |
| Married/partnered |  | -0.003 | 0.009 |  | -0.118 | -0.129 |
|  |  | (0.104) | (0.104) |  | (0.099) | (0.100) |
| Own self-related health(0-10) |  | -0.099*** | -0.106*** |  | -0.104*** | -0.115*** |
|  |  | (0.025) | (0.026) |  | (0.025) | (0.025) |
| *Personality traits* |  |  |  |  |  |  |
| Extraversion |  |  | 0.075*** |  |  | 0.057** |
|  |  |  | (0.025) |  |  | (0.024) |
| Agreeableness |  |  | 0.081*** |  |  | 0.026 |
|  |  |  | (0.03) |  |  | (0.029) |
| Conscientiousness |  |  | -0.060** |  |  | 0.059* |
|  |  |  | (0.028) |  |  | (0.030) |
| Neuroticism |  |  | -0.004 |  |  | -0.030 |
|  |  |  | (0.024) |  |  | (0.025) |
| Openness |  |  | 0 |  |  | 0.002 |
|  |  |  | (0.027) |  |  | (0.027) |
| *Estimators* |  |  |  |  |  |  |
| Pseudo R^2^ | 0.045 | 0.076 | 0.096 | 0.044 | 0.089 | 0.108 |
| Log pseudo-likelihood | -584 | -524 | -513 | -585 | -529 | -518 |
| AIC | 1182 | 1077 | 1064 | 1183 | 1086 | 1074 |
| BIC | 1218 | 1148 | 1161 | 1220 | 1157 | 1171 |
| *Observations* | 1,302 | 1,180 | 1,180 | 1,295 | 1,207 | 1,207 |
| Notes: Coefficients of standardised risk indicators were reported; ***, **, * represent significant level at the 1%, 5% and 10% significant level, respectively; robust standard errors are shown in parentheses. | | | | | | |

### Table A12. Determinants of heavy consumption of alcohol in the indirect approach

|  |  | Dependent variable: frequency of drinking five or more alcoholic drinks |  |  |  |  |  |  |
| --- | --- | --- | --- | --- | --- | --- | --- | --- |
|  | | Version 1 [model] | | | | Version 2 [model] | | |
|  | | [1] | [2] | [3] | [1] | | [2] | [3] |
| *Risk preferences* | |  |  |  |  | |  |  |
| The risk indicator | | 0.080** | 0.090** | 0.080** | 0.004 | | -0.004 | 0.000 |
|  | | (0.035) | (0.037) | (0.038) | (0.035) | | (0.036) | (0.036) |
| *Socio-demographics* | |  |  |  |  | |  |  |
| Age(years) | | 0.017 | 0.022* | 0.030** | 0.030*** | | 0.037*** | 0.049*** |
|  | | (0.012) | (0.013) | (0.013) | (0.011) | | (0.012) | (0.013) |
| Age squared/100 | | -0.037*** | -0.042*** | -0.050*** | -0.048*** | | -0.054*** | -0.066*** |
|  | | (0.013) | (0.014) | (0.014) | (0.012) | | (0.013) | (0.013) |
| Male | | 0.423*** | 0.377*** | 0.365*** | 0.451*** | | 0.395*** | 0.385*** |
|  | | (0.067) | (0.071) | (0.074) | (0.068) | | (0.072) | (0.076) |
| White | | 0.167 | 0.158 | 0.151 | -0.264** | | -0.225* | -0.249* |
|  | | (0.153) | (0.174) | (0.173) | (0.130) | | (0.135) | (0.134) |
| Born in UK | | 0.397*** | 0.417*** | 0.408*** | 0.287** | | 0.313** | 0.372*** |
|  | | (0.137) | (0.152) | (0.149) | (0.130) | | (0.134) | (0.132) |
| Christian | |  | 0.004 | -0.009 |  | | -0.106 | -0.127* |
|  | |  | (0.071) | (0.071) |  | | (0.073) | (0.074) |
| Higher education | |  | -0.105 | -0.128* |  | | -0.025 | -0.014 |
|  | |  | (0.071) | (0.071) |  | | (0.070) | (0.071) |
| Unemployed | |  | -0.149 | -0.215* |  | | 0.139 | 0.109 |
|  | |  | (0.134) | (0.129) |  | | (0.213) | (0.213) |
| Household income | |  | 0.027 | 0.015 |  | | 0.067*** | 0.051** |
|  | |  | (0.025) | (0.025) |  | | (0.022) | (0.022) |
| Sick or disabled | |  | -0.274 | -0.321 |  | | -0.115 | -0.120 |
|  | |  | (0.219) | (0.227) |  | | (0.243) | (0.252) |
| Married/partnered | |  | -0.066 | -0.047 |  | | -0.135* | -0.104 |
|  | |  | (0.077) | (0.077) |  | | (0.075) | (0.076) |
| Own self-related health(0-10) | |  | -0.033 | -0.038* |  | | -0.051** | -0.043** |
|  | |  | (0.023) | (0.023) |  | | (0.020) | (0.021) |
| *Personality traits* | |  |  |  |  | |  |  |
| Extraversion | |  |  | 0.098*** |  | |  | 0.112*** |
|  | |  |  | (0.021) |  | |  | (0.02) |
| Agreeableness | |  |  | -0.003 |  | |  | -0.023 |
|  | |  |  | (0.022) |  | |  | (0.023) |
| Conscientiousness | |  |  | -0.092*** |  | |  | -0.082*** |
|  | |  |  | (0.023) |  | |  | (0.022) |
| Neuroticism | |  |  | -0.011 |  | |  | 0.008 |
|  | |  |  | (0.019) |  | |  | (0.020) |
| Openness | |  |  | 0.005 |  | |  | 0.004 |
|  | |  |  | (0.021) |  | |  | (0.020) |
| *Estimators* | |  |  |  |  | |  |  |
| Pseudo R^2^ | | 0.038 | 0.038 | 0.053 | 0.030 | | 0.036 | 0.053 |
| Log pseudo-likelihood | | -1499 | -1378 | -1357 | -1493 | | -1396 | -1371 |
| AIC | | 3019 | 2790 | 2757 | 3005 | | 2826 | 2787 |
| BIC | | 3069 | 2874 | 2866 | 3055 | | 2910 | 2896 |
| *Observations* | | 1,129 | 1,030 | 1,030 | 1,119 | | 1,048 | 1,048 |
| Notes: Coefficients of standardised risk indicators were reported; ***, **, * represent significant level at the 1%, 5% and 10% significant level, respectively; robust standard errors are shown in parentheses. | | | | | | | | |

### Table A13. Determinants of non-adherence to take a full course of antibiotics in the indirect approach

|  | Dependent variable: Non-adherence to take a full course of antibiotics | | | | | | |
| --- | --- | --- | --- | --- | --- | --- | --- |
|  | Version 1 [model] | | | Version 2 [model] | | | |
|  | [1] | [2] | [3] | | [1] | [2] | [3] |
| *Risk preferences* |  |  |  | |  |  |  |
| The risk indicator | 0.130*** | 0.138*** | 0.131*** | | -0.008 | -0.020 | -0.024 |
|  | (0.042) | (0.045) | (0.045) | | (0.041) | (0.044) | (0.045) |
| *Socio-demographics* |  |  |  | |  |  |  |
| Age(years) | -0.027* | -0.035** | -0.031* | | -0.029* | -0.02 | -0.007 |
|  | (0.014) | (0.015) | (0.016) | | (0.015) | (0.017) | (0.018) |
| Age squared/100 | 0.008 | 0.016 | 0.012 | | 0.008 | -0.003 | -0.015 |
|  | (0.015) | (0.017) | (0.017) | | (0.017) | (0.020) | (0.020) |
| Male | 0.418*** | 0.461*** | 0.433*** | | 0.304*** | 0.357*** | 0.289*** |
|  | (0.081) | (0.086) | (0.088) | | (0.082) | (0.087) | (0.089) |
| White | -0.341** | -0.401*** | -0.416*** | | -0.058 | -0.067 | -0.075 |
|  | (0.135) | (0.149) | (0.150) | | (0.133) | (0.143) | (0.141) |
| Born in UK | -0.269** | -0.329** | -0.345** | | -0.472*** | -0.528*** | -0.517*** |
|  | (0.132) | (0.143) | (0.143) | | (0.118) | (0.122) | (0.122) |
| Christian |  | 0.092 | 0.094 | |  | -0.057 | -0.001 |
|  |  | (0.085) | (0.085) | |  | (0.087) | (0.088) |
| Higher education |  | -0.132 | -0.146* | |  | -0.132 | -0.130 |
|  |  | (0.086) | (0.086) | |  | (0.087) | (0.089) |
| Unemployed |  | -0.113 | -0.140 | |  | -0.271 | -0.296 |
|  |  | (0.174) | (0.175) | |  | (0.223) | (0.218) |
| Household income |  | -0.030 | -0.034 | |  | -0.029 | -0.049* |
|  |  | (0.031) | (0.032) | |  | (0.028) | (0.028) |
| Sick or disabled |  | -0.091 | -0.081 | |  | -0.247 | -0.238 |
|  |  | (0.259) | (0.260) | |  | (0.262) | (0.266) |
| Married/partnered |  | 0.136 | 0.135 | |  | -0.083 | -0.060 |
|  |  | (0.095) | (0.095) | |  | (0.093) | (0.095) |
| Own self-related health(0-10) |  | 0.013 | 0.014 | |  | -0.013 | -0.009 |
|  |  | (0.026) | (0.027) | |  | (0.024) | (0.025) |
| *Personality traits* |  |  |  | |  |  |  |
| Extraversion |  |  | 0.028 | |  |  | 0.013 |
|  |  |  | (0.024) | |  |  | (0.024) |
| Agreeableness |  |  | -0.030 | |  |  | -0.134*** |
|  |  |  | (0.027) | |  |  | (0.029) |
| Conscientiousness |  |  | -0.054** | |  |  | -0.104*** |
|  |  |  | (0.026) | |  |  | (0.028) |
| Neuroticism |  |  | -0.010 | |  |  | -0.032 |
|  |  |  | (0.023) | |  |  | (0.024) |
| Openness |  |  | 0 | |  |  | -0.007 |
|  |  |  | (0.025) | |  |  | (0.025) |
| *Estimators* |  |  |  | |  |  |  |
| Pseudo R^2^ | 0.075 | 0.079 | 0.084 | | 0.059 | 0.072 | 0.101 |
| Log pseudo-likelihood | -814 | -742 | -738 | | -796 | -726 | -703 |
| AIC | 1647 | 1516 | 1518 | | 1610 | 1485 | 1449 |
| BIC | 1693 | 1596 | 1624 | | 1657 | 1566 | 1556 |
| *Observations* | 1,284 | 1,165 | 1,165 | | 1,276 | 1,190 | 1,190 |
| Notes: Coefficients of standardised risk indicators were reported; ***, **, * represent significant level at the 1%, 5% and 10% significant level, respectively; robust standard errors are shown in parentheses. | | | | | | | |

## Sensitivity analysis

### Table A14. Association between any risky behaviour and direct risk indicators in the full and restricted samples

|  | Dependent variable: any risky behaviour | | | |
| --- | --- | --- | --- | --- |
|  | General | | Health | |
|  | Full sample | Restricted sample | Full sample | Restricted sample |
| *Risk preferences* |  |  |  |  |
| Risk indicator | 0.093*** | 0.132*** | 0.180*** | 0.191*** |
|  | (0.025) | (0.031) | (0.024) | (0.031) |
| *Socio-demographics* |  |  |  |  |
| Age(years) | 0.030*** | 0.032*** | 0.027*** | 0.027** |
|  | (0.009) | (0.011) | (0.009) | (0.011) |
| Age squared/100 | -0.029*** | -0.026** | -0.025*** | -0.020* |
|  | (0.009) | (0.011) | (0.009) | (0.011) |
| Male | 0.414*** | 0.401*** | 0.405*** | 0.406*** |
|  | (0.047) | (0.059) | (0.047) | (0.058) |
| White | 0.152* | 0.341*** | 0.152* | 0.328*** |
|  | (0.081) | (0.107) | (0.082) | (0.108) |
| Born in UK | 0.314*** | 0.231** | 0.296*** | 0.192* |
|  | (0.080) | (0.104) | (0.080) | (0.103) |
| Christian | -0.081* | -0.129** | -0.085* | -0.134** |
|  | (0.046) | (0.057) | (0.047) | (0.057) |
| Higher education | -0.103** | -0.104* | -0.109** | -0.101* |
|  | (0.047) | (0.057) | (0.047) | (0.057) |
| Unemployed | -0.182* | -0.386*** | -0.184* | -0.383*** |
|  | (0.110) | (0.140) | (0.111) | (0.141) |
| Household income | 0.054*** | 0.051** | 0.057*** | 0.057*** |
|  | (0.017) | (0.021) | (0.017) | (0.021) |
| Sick or disabled | -0.148 | -0.153 | -0.125 | -0.123 |
|  | (0.114) | (0.137) | (0.115) | (0.138) |
| Married/partnered | 0.108** | 0.101* | 0.099** | 0.099 |
|  | (0.049) | (0.060) | (0.049) | (0.060) |
| Own self-related health(0-10) | -0.016 | -0.017 | -0.014 | -0.011 |
|  | (0.012) | (0.016) | (0.012) | (0.016) |
| *Personality traits* |  |  |  |  |
| Extraversion | 0.081*** | 0.071*** | 0.083*** | 0.077*** |
|  | (0.013) | (0.015) | (0.013) | (0.015) |
| Agreeableness | -0.016 | -0.020 | -0.012 | -0.013 |
|  | (0.014) | (0.017) | (0.014) | (0.017) |
| Conscientiousness | -0.032** | -0.019 | -0.013 | 0.001 |
|  | (0.014) | (0.017) | (0.014) | (0.017) |
| Neuroticism | -0.003 | -0.001 | -0.006 | -0.006 |
|  | (0.012) | (0.015) | (0.012) | (0.015) |
| Openness | -0.007 | -0.008 | -0.001 | -0.003 |
|  | (0.013) | (0.016) | (0.013) | (0.016) |
| *Observations* | 3,583 | 2,367 | 3,583 | 2,367 |
| Notes: Coefficients of standardised risk indicators were reported; ***, **, * represent significant level at the 1%, 5% and 10% significant level, respectively; robust standard errors are shown in parentheses. | | | | |

### Table A15. Association between number of risky health behaviours and direct risk indicators in the full and restricted samples

|  | Dependent variable: number of risky health behaviour | | | |
| --- | --- | --- | --- | --- |
|  | General | | Health | |
|  | Full sample | Restricted sample | Full sample | Restricted sample |
| *Risk preferences* | 0.097*** | 0.124*** | 0.199*** | 0.204*** |
| Risk indicator | (0.022) | (0.028) | (0.021) | (0.027) |
| *Socio-demographics* |  |  |  |  |
| Age(years) | 0.036*** | 0.036*** | 0.033*** | 0.030*** |
|  | (0.008) | (0.010) | (0.008) | (0.010) |
| Age squared/100 | -0.042*** | -0.037*** | -0.037*** | -0.030*** |
|  | (0.008) | (0.010) | (0.008) | (0.010) |
| Male | 0.383*** | 0.392*** | 0.372*** | 0.394*** |
|  | (0.042) | (0.052) | (0.041) | (0.052) |
| White | 0.123 | 0.284** | 0.120 | 0.275** |
|  | (0.079) | (0.111) | (0.080) | (0.113) |
| Born in UK | 0.315*** | 0.264*** | 0.297*** | 0.228** |
|  | (0.075) | (0.098) | (0.075) | (0.097) |
| Christian | -0.033 | -0.090* | -0.037 | -0.094* |
|  | (0.040) | (0.050) | (0.040) | (0.050) |
| Higher education | -0.130*** | -0.130*** | -0.136*** | -0.128** |
|  | (0.040) | (0.050) | (0.040) | (0.050) |
| Unemployed | -0.171 | -0.364** | -0.170 | -0.358** |
|  | (0.113) | (0.144) | (0.113) | (0.144) |
| Household income | 0.036*** | 0.023 | 0.037*** | 0.028* |
|  | (0.013) | (0.016) | (0.013) | (0.016) |
| Sick or disabled | -0.189* | -0.192 | -0.158 | -0.155 |
|  | (0.109) | (0.138) | (0.110) | (0.140) |
| Married/partnered | 0.060 | 0.053 | 0.052 | 0.054 |
|  | (0.043) | (0.054) | (0.044) | (0.054) |
| Own self-related health(0-10) | -0.028*** | -0.033** | -0.026** | -0.027* |
|  | (0.010) | (0.014) | (0.010) | (0.014) |
| *Personality traits* |  |  |  |  |
| Extraversion | 0.069*** | 0.062*** | 0.071*** | 0.067*** |
|  | (0.011) | (0.013) | (0.011) | (0.013) |
| Agreeableness | 0.004 | 0.002 | 0.009 | 0.009 |
|  | (0.013) | (0.015) | (0.013) | (0.015) |
| Conscientiousness | -0.043*** | -0.030** | -0.021* | -0.007 |
|  | (0.012) | (0.015) | (0.012) | (0.015) |
| Neuroticism | -0.014 | -0.006 | -0.018* | -0.011 |
|  | (0.011) | (0.013) | (0.011) | (0.013) |
| Openness | -0.006 | -0.008 | -0.000 | -0.003 |
|  | (0.011) | (0.014) | (0.011) | (0.014) |
| *Observations* | 3,558 | 2,355 | 3,558 | 2,355 |
| Notes: Coefficients of standardised risk indicators were reported; ***, **, * represent significant level at the 1%, 5% and 10% significant level, respectively; robust standard errors are shown in parentheses. | | | | |

### Table A16. Association between smoking status and direct risk indicators in the full and restricted samples

|  | Dependent variable: smoking status | | | |
| --- | --- | --- | --- | --- |
|  | General | | Health | |
|  | Full sample | Restricted sample | Full sample | Restricted sample |
| *Risk preferences* |  |  |  |  |
| The risk indicator | 0.115*** | 0.120*** | 0.253*** | 0.233*** |
|  | (0.027) | (0.035) | (0.025) | (0.032) |
| *Socio-demographics* |  |  |  |  |
| Age(years) | 0.054*** | 0.056*** | 0.051*** | 0.049*** |
|  | (0.009) | (0.012) | (0.009) | (0.012) |
| Age squared/100 | -0.081*** | -0.079*** | -0.075*** | -0.071*** |
|  | (0.010) | (0.013) | (0.01) | (0.013) |
| Male | 0.296*** | 0.266*** | 0.273*** | 0.259*** |
|  | (0.048) | (0.062) | (0.048) | (0.062) |
| White | 0.073 | 0.234** | 0.085 | 0.244** |
|  | (0.079) | (0.114) | (0.08) | (0.117) |
| Born in UK | 0.313*** | 0.145 | 0.281*** | 0.099 |
|  | (0.083) | (0.112) | (0.084) | (0.112) |
| Christian | 0.041 | 0.023 | 0.039 | 0.023 |
|  | (0.047) | (0.059) | (0.047) | (0.060) |
| Higher education | -0.253*** | -0.319*** | -0.264*** | -0.321*** |
|  | (0.048) | (0.061) | (0.048) | (0.061) |
| Unemployed | 0.046 | 0.008 | 0.054 | 0.022 |
|  | (0.108) | (0.138) | (0.107) | (0.137) |
| Household income | -0.015 | -0.026 | -0.014 | -0.022 |
|  | (0.017) | (0.022) | (0.017) | (0.022) |
| Sick or disabled | 0.034 | 0.067 | 0.087 | 0.127 |
|  | (0.122) | (0.152) | (0.124) | (0.154) |
| Married/partnered | 0.058 | -0.04 | 0.044 | -0.042 |
|  | (0.052) | (0.066) | (0.053) | (0.066) |
| Own self-rated health(0-10) | -0.072*** | -0.103*** | -0.071*** | -0.097*** |
|  | (0.012) | (0.017) | (0.012) | (0.017) |
| *Personality traits* |  |  |  |  |
| Extraversion | 0.062*** | 0.057*** | 0.062*** | 0.059*** |
|  | (0.013) | (0.016) | (0.013) | (0.016) |
| Agreeableness | 0.037** | 0.054*** | 0.045*** | 0.064*** |
|  | (0.015) | (0.019) | (0.015) | (0.019) |
| Conscientiousness | -0.046*** | -0.02 | -0.017 | 0.008 |
|  | (0.014) | (0.018) | (0.015) | (0.019) |
| Neuroticism | -0.007 | -0.008 | -0.012 | -0.012 |
|  | (0.013) | (0.017) | (0.013) | (0.016) |
| Openness | -0.013 | -0.003 | -0.006 | 0.001 |
|  | (0.014) | (0.017) | (0.014) | (0.017) |
| *Observations* | 3,616 | 2,387 | 3,616 | 2,387 |
| Notes: Coefficients of standardised risk indicators were reported; ***, **, * represent significant level at the 1%, 5% and 10% significant level, respectively; robust standard errors are shown in parentheses. | | | | |

### Table A17. Association between regular smoking and direct risk indicators in the full and restricted samples

|  | Dependent variable: regular smoker | | | |
| --- | --- | --- | --- | --- |
|  | General | | Health | |
|  | Full sample | Restricted sample | Full sample | Restricted sample |
| *Risk preferences* |  |  |  |  |
| The risk indicator | 0.114*** | 0.113*** | 0.268*** | 0.257*** |
|  | (0.029) | (0.038) | (0.027) | (0.036) |
| *Socio-demographics* |  |  |  |  |
| Age(years) | 0.064*** | 0.073*** | 0.061*** | 0.067*** |
|  | (0.010) | (0.013) | (0.010) | (0.013) |
| Age squared/100 | -0.088*** | -0.092*** | -0.083*** | -0.085*** |
|  | (0.011) | (0.014) | (0.011) | (0.014) |
| Male | 0.279*** | 0.244*** | 0.254*** | 0.237*** |
|  | (0.052) | (0.068) | (0.052) | (0.068) |
| White | 0.112 | 0.228* | 0.118 | 0.234* |
|  | (0.091) | (0.133) | (0.094) | (0.137) |
| Born in UK | 0.410*** | 0.234* | 0.379*** | 0.190 |
|  | (0.098) | (0.133) | (0.099) | (0.135) |
| Christian | 0.023 | -0.035 | 0.018 | -0.036 |
|  | (0.051) | (0.066) | (0.051) | (0.066) |
| Higher education | -0.249*** | -0.358*** | -0.265*** | -0.365*** |
|  | (0.052) | (0.068) | (0.052) | (0.068) |
| Unemployed | 0.122 | 0.074 | 0.129 | 0.093 |
|  | (0.112) | (0.147) | (0.111) | (0.146) |
| Household income | -0.018 | -0.040 | -0.020 | -0.040 |
|  | (0.018) | (0.025) | (0.018) | (0.026) |
| Sick or disabled | 0.102 | 0.108 | 0.160 | 0.181 |
|  | (0.122) | (0.152) | (0.124) | (0.154) |
| Married/partnered | 0.048 | -0.051 | 0.034 | -0.052 |
|  | (0.056) | (0.072) | (0.057) | (0.072) |
| Own self-rated health(0-10) | -0.072*** | -0.107*** | -0.072*** | -0.102*** |
|  | (0.013) | (0.018) | (0.013) | (0.018) |
| *Personality traits* |  |  |  |  |
| Extraversion | 0.049*** | 0.050*** | 0.049*** | 0.051*** |
|  | (0.014) | (0.018) | (0.014) | (0.017) |
| Agreeableness | 0.036** | 0.051** | 0.044*** | 0.061*** |
|  | (0.016) | (0.021) | (0.016) | -0.021 |
| Conscientiousness | -0.030* | -0.003 | 0.001 | 0.029 |
|  | (0.016) | (0.020) | (0.016) | (0.021) |
| Neuroticism | -0.012 | -0.007 | -0.018 | -0.011 |
|  | (0.014) | (0.018) | (0.014) | (0.018) |
| Openness | -0.008 | -0.005 | -0.001 | -0.003 |
|  | (0.015) | (0.019) | (0.016) | (0.019) |
| *Observations* | 3,616 | 2,387 | 3,616 | 2,387 |
| Notes: Coefficients of standardised risk indicators were reported; ***, **, * represent significant level at the 1%, 5% and 10% significant level, respectively; robust standard errors are shown in parentheses. | | | | |

### Table A18. Association between non-adherence to taking a full course of antibiotics and direct risk indicators in the full and restricted samples

|  | Dependent variable: Non-adherence to taking a full course of antibiotics | | | |
| --- | --- | --- | --- | --- |
|  | General | | Health | |
|  | Full sample | Restricted sample | Full sample | Restricted sample |
| *Risk preferences* |  |  |  |  |
| The risk indicator | 0.113*** | 0.148*** | 0.197*** | 0.234*** |
|  | (0.029) | (0.038) | (0.025) | (0.033) |
| *Socio-demographics* |  |  |  |  |
| Age(years) | -0.017* | -0.021* | -0.021** | -0.028** |
|  | (0.009) | (0.012) | (0.009) | (0.012) |
| Age squared/100 | -0.004 | 0.002 | 0.002 | 0.010 |
|  | (0.010) | (0.013) | (0.010) | (0.013) |
| Male | 0.275*** | 0.325*** | 0.257*** | 0.319*** |
|  | (0.049) | (0.063) | (0.049) | (0.063) |
| White | -0.322*** | -0.217** | -0.325*** | -0.221** |
|  | (0.074) | (0.102) | (0.075) | (0.102) |
| Born in UK | -0.301*** | -0.418*** | -0.339*** | -0.484*** |
|  | (0.074) | (0.093) | (0.074) | (0.094) |
| Christian | 0.009 | 0.052 | 0.007 | 0.057 |
|  | (0.048) | (0.061) | (0.048) | (0.061) |
| Higher education | -0.138*** | -0.143** | -0.141*** | -0.139** |
|  | (0.049) | (0.062) | (0.049) | (0.062) |
| Unemployed | -0.051 | -0.206 | -0.044 | -0.189 |
|  | (0.107) | (0.134) | (0.108) | (0.137) |
| Household income | -0.029* | -0.044** | -0.027 | -0.037* |
|  | (0.018) | (0.021) | (0.018) | (0.022) |
| Sick or disabled | -0.092 | -0.102 | -0.062 | -0.045 |
|  | (0.148) | (0.186) | (0.149) | (0.188) |
| Married/partnered | 0.009 | 0.047 | -0.002 | 0.043 |
|  | (0.053) | (0.067) | (0.054) | (0.068) |
| Own self-rated health(0-10) | -0.021* | 0.004 | -0.018 | 0.013 |
|  | (0.013) | (0.019) | (0.013) | (0.019) |
| *Personality traits* |  |  |  |  |
| Extraversion | 0.008 | 0.001 | 0.010 | 0.005 |
|  | (0.014) | (0.017) | (0.014) | (0.017) |
| Agreeableness | -0.086*** | -0.079*** | -0.082*** | -0.072*** |
|  | (0.015) | (0.02) | (0.015) | (0.02) |
| Conscientiousness | -0.086*** | -0.079*** | -0.065*** | -0.053*** |
|  | (0.015) | (0.019) | (0.015) | (0.019) |
| Neuroticism | -0.018 | -0.007 | -0.021 | -0.010 |
|  | (0.014) | (0.017) | (0.014) | (0.017) |
| Openness | -0.027* | -0.011 | -0.021 | -0.005 |
|  | (0.014) | (0.018) | (0.014) | (0.017) |
| *Observations* | 3,558 | 2,355 | 3,558 | 2,355 |
| Notes: Coefficients of standardised risk indicators were reported; ***, **, * represent significant level at the 1%, 5% and 10% significant level, respectively; robust standard errors are shown in parentheses. | | | | |

## Empirical results: comparison between different risk indicators using alternative models further controlling for health state A and B

### Figure A3. Comparison between different risk indicators using regression models for different health behaviours

## Empirical results: comparison between different risk indicators in the direct and indirect approach - marginal effects reported

### Table A19. Comparison between different risk indicators using regression models for different health behaviours-Marginal effects reported

|  | The direct approach | | | The indirect approach | | | | |
| --- | --- | --- | --- | --- | --- | --- | --- | --- |
|  | General | Health | | | Overall | Version1 | | Version2 |
| Any risky behaviour |  |  | | |  |  | |  |
| Yes | 0.051*** | 0.074*** | | | 0.016 | 0.023 | | 0.005 |
|  | (0.012) | (0.012) | | | (0.011) | (0.015) | | (0.015) |
| *Observations* | 2,367 | 2,367 | | | 2,367 | 1,172 | | 1,195 |
| Number of risky behaviours |  |  | | |  |  | |  |
| None | -0.048*** | -0.079*** | | | -0.013 | -0.026** | | 0.001 |
|  | (0.011) | (0.010) | | | (0.009) | (0.013) | | (0.014) |
| One | 0.025*** | 0.042*** | | | 0.007 | 0.014** | | -0.001 |
|  | (0.006) | (0.006) | | | (0.005) | (0.007) | | (0.007) |
| Two | 0.022*** | 0.035*** | | | 0.006 | 0.011** | | -0.001 |
|  | (0.005) | (0.005) | | | (0.004) | (0.006) | | (0.007) |
| Three | 0.001** | 0.002*** | | | 0.000 | 0.001* | | -0.000 |
|  | (0.000) | (0.001) | | | (0.000) | (0.001) | | (0.000) |
| *Observations* | 2,355 | 2,355 | | | 2,355 | 1,165 | | 1,190 |
| Smoking status |  |  | | |  |  | |  |
| Non-smoker | -0.036*** | -0.070*** | | | -0.010 | -0.017 | | -0.001 |
|  | (0.011) | (0.010) | | | (0.009) | (0.013) | | (0.012) |
| Occasional smoker | 0.007*** | 0.014*** | | | 0.002 | 0.003 | | 0.000 |
|  | (0.002) | (0.002) | | | (0.002) | (0.003) | | (0.002) |
| Regular smoker | 0.029*** | 0.056*** | | | 0.008 | 0.014 | | 0.001 |
|  | (0.009) | (0.008) | | | (0.007) | (0.010) | | (0.010) |
| *Observations* | 2,387 | 2,387 | | | 2,387 | 1,180 | | 1,207 |
| Regular smoker |  |  | | |  |  | |  |
| Yes | 0.028*** | 0.061*** | | | 0.015* | 0.027** | | -0.001 |
|  | (0.009) | (0.008) | | | (0.008) | (0.011) | | (0.011) |
| *Observations* | 2,387 | 2,387 | | | 2,387 | 1,180 | | 1,207 |
| Frequency of drinking five or more alcoholic drinks | | | | | | | | |
| Never | -0.044*** | -0.052*** | | | -0.016* | -0.030** | | -0.000 |
|  | (0.011) | (0.010) | | | (0.010) | (0.014) | | (0.013) |
| Less than once a month | 0.004** | 0.004*** | | | 0.001 | 0.003 | | 0.000 |
|  | (0.001) | (0.002) | | | (0.001) | (0.002) | | (0.001) |
| 1 to 3 times a month | 0.016*** | 0.019*** | | | 0.006* | 0.010** | | 0.000 |
|  | (0.004) | (0.004) | | | (0.003) | (0.005) | | (0.005) |
| Once or twice a week | 0.017*** | 0.020*** | | | 0.006* | 0.011** | | 0.000 |
|  | (0.004) | (0.004) | | | (0.004) | (0.006) | | (0.005) |
| Three or more times a week | 0.008*** | 0.009*** | | | 0.003* | 0.005** | | 0.000 |
|  | (0.002) | (0.002) | | | (0.002) | (0.002) | | (0.002) |
| *Observations* | 2,078 | 2,078 | | | 2,078 | 1,030 | | 1,048 |
| Non-adherence taking a full course of antibiotics | | |  | | | |  | |
| Definitely not | -0.041*** | -0.064*** | | | -0.016* | -0.038*** | | 0.006 |
|  | (0.010) | (0.009) | | | (0.009) | (0.013) | | (0.012) |
| Probably not | 0.029*** | 0.046*** | | | 0.011* | 0.028*** | | -0.004 |
|  | (0.008) | (0.007) | | | (0.006) | (0.010) | | (0.008) |
| Probably | 0.009*** | 0.014*** | | | 0.004* | 0.007*** | | -0.002 |
|  | (0.002) | (0.002) | | | (0.002) | (0.003) | | (0.003) |
| Definitely | 0.002*** | 0.003*** | | | 0.001 | 0.002** | | -0.000 |
|  | (0.001) | (0.001) | | | (0.001) | (0.001) | | (0.001) |
| *Observations* | 2,355 | 2,355 | | | 2,355 | 1,165 | | 1,190 |
| Notes: Marginal effects of standardised risk indicators were reported; ***, **, * represent significant level at the 1%, 5% and 10% significant level, respectively; robust standard errors are shown in parentheses; ordered probit model was conducted for number of risky behaviours, smoking status, frequency of five alcoholic drinks and non-adherence taking a full course of antibiotics; probit model was conducted for any risky behaviour and regular smoker. | | | | | | | | |
